# Autophagy and mitophagy at the synapse and beyond: implications for learning, memory and neurological disorders

**DOI:** 10.1080/15548627.2025.2581217

**Published:** 2025-11-23

**Authors:** Jiayi Lu, Damian N. Di Florio, Patricia Boya, Sandra Maday, Wolfdieter Springer, Charleen T. Chu

**Affiliations:** a Department of Pathology/Division of Neuropathology, University of Pittsburgh School of Medicine, Pittsburgh, PA, USA; b Department of Neuroscience, Mayo Clinic, Jacksonville, FL, USA; c Department of Neuroscience and Movement Sciences, University of Fribourg, Fribourg, Switzerland; d Department of Neuroscience, Perelman School of Medicine, University of Pennsylvania, Philadelphia, PA, USA

**Keywords:** Biomarkers, dementia, dendritic spines, mitochondria, neurodegenerative diseases, neurodevelopmental disorders, Parkinson disease, synaptic plasticity

## Abstract

The human brain is one of the most metabolically active tissues in the body, due in large part to the activity of trillions of synaptic connections. Under normal conditions, macroautophagy/autophagy at the synapse plays a crucial role in synaptic pruning and plasticity, which occurs physiologically in the absence of disease- or aging-related stressors. Disruption of autophagy has profound effects on neuron development, structure, function, and survival. Neurons are dependent upon maintaining high-quality mitochondria, and alterations in selective mitochondrial autophagy (mitophagy) are heavily implicated in both genetic and environmental etiologies of neurodegenerative diseases. The unique spatial and functional demands of neurons result in differences in the regulation of metabolic, autophagic, mitophagic and biosynthetic processes compared to other cell types. Here, we review recent advances in autophagy and mitophagy research with an emphasis on studies involving primary neurons *in vitro* and *in vivo*, glial cells, and iPSC-differentiated neurons. The synaptic functions of genes whose mutations implicate autophagic or mitophagic dysfunction in hereditary neurodegenerative and neurodevelopmental diseases are summarized. Finally, we discuss the diagnostic and therapeutic potentials of autophagy-related pathways.

**Abbreviations**: AD: Alzheimer disease; ALS: amyotrophic lateral sclerosis; APP: amyloid beta precursor protein; ASD: autism-spectrum disorder; BDNF: brain-derived neurotrophic factor; BPAN: β-propeller protein associated neurodegeneration; CR: caloric restriction; ΔN111: deleted N-terminal region 111 residues; DLG4/PSD95: discs large MAGUK scaffold protein 4; ER: endoplasmic reticulum; FTD: frontotemporal dementia; HD: Huntington disease; LIR: LC3-interacting region; LRRK2: leucine rich repeat kinase 2; LTD: long-term depression; LTP: long-term potentiation; MAP1LC3/LC3: microtubule associated protein 1 light chain 3; OMM: outer mitochondrial membrane; PD: Parkinson spectrum diseases; PGRN: progranulin; PINK1: PTEN induced kinase 1; PRKA/PKA: protein kinase cAMP-activated; PtdIns3P: phosphatidylinositol-3-phosphate; p-S65-Ub: ubiquitin phosphorylated at serine 65; PTM: post-translational modification; TREM2: triggering receptor expressed on myeloid cells 2

The neuron faces unique challenges in maintaining cellular quality over the lifetime. Although we now know that there are sites of adult neurogenesis in the human brain [1], the vast majority of neurons are irreplaceable, surviving on the order of 100 years in long-lived individuals. Autophagy, the process by which damaged or unneeded cellular constituents are targeted to the lysosome for degradation, plays a key role in cellular quality control, regulating differentiation, cell survival and renewal of eukaryotic cells. Given that neurons are highly dependent on mitochondrial respiration, maintaining high quality mitochondria through selective mitochondrial autophagy (mitophagy) is crucial. Disruptions in both autophagy and mitophagy have been strongly implicated in neurodegenerative and neurodevelopmental diseases.

The morphological structure of the neuron presents special challenges in quality control. Projection neurons extend long, thin axons that may be over a meter in length to communicate with downstream neurons, muscle cells and other targets. Neurons also maintain a complex, highly branched dendritic arbor poised to receive signals from other neurons and sensory structures at contact sites called synapses. To support healthy synaptic communication, both presynaptic and postsynaptic sides of the synapse contain specialized membranes and organelles that support rapid and repeated electrochemical activity. Many of these processes require tight control over intracellular calcium, and disruptions in mitochondrial calcium homeostasis are implicated in acute and chronic neurodegeneration (reviewed in [2]). Quality control of effete or damaged mitochondria and other peri-synaptic components often involves autophagic degradation, which must be balanced by their biosynthetic replacement at vast molecular distances from the nucleus [3].

This review covers the regulation of autophagy in distinct subcellular compartments of the neuron and the emerging roles of autophagy and mitophagy-linked proteins in synaptic function, plasticity, and neurological diseases. New insights concerning the role of autophagy-related processes in axons and the impact of autophagy- and mitophagy-linked proteins on dendritic spine development, plasticity and learning behaviors are highlighted in the first major section. The second section presents a comprehensive review of studies implicating autophagy and mitophagy dysregulation in neurodegenerative and neurodevelopmental diseases. Classic knockout mouse studies reveal an important role for the autophagy proteins ATG5 and ATG7 in neuronal development, proteostasis and neuronal survival [4,5]. Multiple subsequent studies reveal that more subtle disruptions of autophagy and mitophagy, which may occur due to aging, genetic risk factors, or other disease processes, show profound effects on neuron structure and function. Current diagnostic and therapeutic approaches based on our understanding of neuronal autophagy and mitophagy in health and disease are summarized at the end of this section.

## Autophagy and mitophagy in neuron health and function

In addition to its roles in maintaining neuron health and survival, protein degradation at the synapse is crucial in regulating neuronal function itself. It has been known for some time that inhibiting proteasomal activity in the brain disrupts synaptogenesis [6], maintenance of long-term potentiation [7], and both formation and extinction of memories in mice [8–10]. Likewise, emerging studies underscore critical roles for autophagy and/or mitophagy-linked proteins at the synapse. These include synaptic pruning [11,12], regulating the density and morphology of dendritic spines [13], and behavioral flexibility in learning paradigms [14]. To effectively contextualize this exciting new literature, it is necessary to briefly review major mechanisms involved in autophagy and mitophagy, and to highlight key facets of the structural architecture underlying neuronal function.

### Introduction to autophagy, mitophagy and neuronal architecture

#### Major steps and mediators in autophagy

Autophagy, or self-eating, consists of several pathways that result in delivery of cellular constituents to the lysosome for degradation. Macroautophagy has been observed in all eukaryotic cells studied, including both single cell and multicellular organisms. It involves intracellular membrane extensions to form new organelles called autophagosomes that envelope intracellular cargoes. Autophagosome fusion with endocytic-lysosomal structures serve to deliver additional cargoes and degradative enzymes. Chaperone-mediated autophagy is observed in avian and mammalian cells, consisting of direct import of proteins with a KDEL-like motif into the lysosome in a process dependent upon HSPA8/Hsc70 chaperones and a specific isoform of the LAMP2 protein, LAMP2A. Microautophagy is a less understood process involving lysosomal membrane invaginations that sample nearby proteins. Unless otherwise stated, the term “autophagy” refers to macroautophagy in subsequent sections of this review.

**Figure 1.**
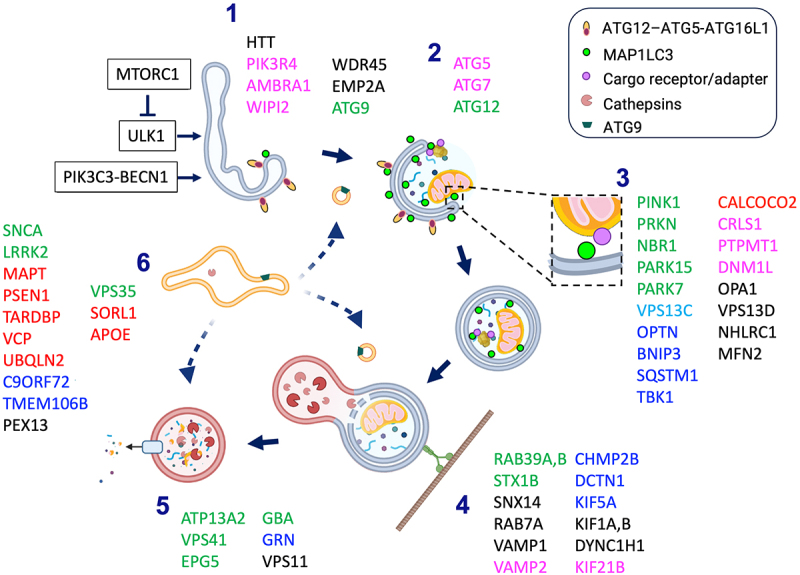
Major steps in autophagy and genes linked to neurological diseases. (1) *Initiation*. Shifts in the balance of MTORC1 vs. autophagy-inducing signals result in phosphorylation of phosphatidylinositols, recruitment of ATG12–ATG5-ATG16L1 and ATG9-containing lipid vesicles to initiate autophagy. (2) *Autophagosome formation*. Continued covalent deposition of MAP1LC3 is accompanied by membrane extension and closure to form the double membrane autophagosome. (3) *Cargo targeting*. As the autophagosome forms, molecular interactions between MAP1LC3, or other Atg8-family members, and cargo surface molecules mediate selective autophagy. These may involve direct MAP1LC3 interactions with LIR receptors, membrane components such as mitochondrial cardiolipin, or indirect interactions mediated by bi-functional ubiquitin- and MAP1LC3-interacting proteins such as SQSTM1 and OPTN. (4) *Trafficking and Maturation*. Autophagosomes mature through fusions with other vesicular organelles, resulting in acidification and delivery of lysosomal hydrolases, as they are transported along microtubules. (5) *Degradation*. The final step in autophagy involves cargo degradation within lysosomes, and the release of degradation products for re-utilization. (6) *Indirect regulation*. The retromer system facilitates sorting and recycling of ATG9-containing membranes and the delivery of lysosomal hydrolases. Other genes elicit indirect effects on autophagy via changes in protein aggregation, cell signaling, intracellular transport or lysosomal acidification. Gene products implicated in the Parkinson disease spectrum (green), ALS-FTD spectrum (blue), Alzheimer disease (red), neurodevelopmental disorders (pink) or other diseases including those implicated in more than one category (black) are shown at the steps they have been proposed to affect. Created using BioRender and Microsoft Powerpoint 16.77.1. Chu, C. (2025) https://BioRender.com/wq3e8ot.

The major steps of autophagy are shown in Figure 1. Cellular integration of signals that promote or suppress autophagy are often tied to the nutritive status of the cell. In particular, the MTOR (mechanistic target of rapamycin kinase) complex 1 (MTORC1) suppresses autophagy by inhibiting ULK1 (unc-51 like autophagy activating kinase 1). ULK1 is also regulated by AMPK, a key energy sensor in the cell, creating an AMPK-MTORC1-ULK1 regulatory triangle that integrates nutrient and metabolic inputs for autophagy induction [15]. A series of ubiquitin-like conjugation steps regulate classic autophagosome formation, although noncanonical pathways exist. In the canonical pathway, ULK1 recruits and activates BECN1/Beclin 1 and the class III phosphatidylinositol 3-kinase (PIK3C3) to modify membranes such as those associated with the endoplasmic reticulum, resulting in deposition of ATG16L1 that attracts membrane localization of ATG12–ATG5, the first ubiquitin-like conjugation. The ATG12–ATG5-ATG16L1 complex in turn promotes the conjugation of MAP1LC3/LC3 (microtubule associated protein 1 light chain 3), homologous to yeast Atg8, to phosphatidylethanolamine. This step is necessary for expansion of phagophore membranes and their curvature and fusion to form a double-membrane early autophagosome. Trafficking and fusion steps result in maturation of the autophagosome into degradative lysosomes. Completion of autophagy depends upon appropriate lysosomal acidification and enzymatic activity. The retromer endosomal trafficking system also affects autophagy by trafficking essential lysosomal enzymes via the endosome-to-Golgi retrieval of M6PR (mannose-6-phosphate receptor, cation dependent), and by regulating recycling of ATG9-containing vesicles for autophagosome biogenesis. Autophagic processes have been heavily studied, and the reader is referred to several excellent review articles for more details [16–18].

It is important to note that the canonical pathway of mammalian autophagy induction was defined primary through studies of nutrient deprivation, in analogy to extensive work in yeast. Non-canonical pathways for initiating autophagosome formation represent additional emerging areas. For example, BECN1-independent autophagy/mitophagy was initially reported in the context of neuronal autophagy induced in response to mitochondrial injury [19]. In contrast to canonical extension of MAP1LC3-bearing membranes around the cargo from a single nucleation point [20], a live-imaging study in hepatocytes showed simultaneous recruitment of MAP1LC3 puncta to multiple spots on mitochondria damaged by photoirradiation, which subsequently fuse [21]. While the orientation of the recruited MAP1LC3 is unknown, possible mechanisms for direct recruitment of MAP1LC3 to the surface of damaged mitochondria include cardiolipin externalization for cargo recognition [22] and/or formation of phosphatidylethanolamine on the outer mitochondrial membrane [23]. The initiation of BECN1-independent autophagy remains poorly understood, but oxidative stress and mitogen-activated protein kinases have been implicated [19,24,25].

#### Introduction to mitophagy

The brain is one of the most metabolically active tissues in the body, weighing in at only 2% of body mass, but consuming 20% of body energy [26]. In rodents, it has been estimated that the axonal compartment consumes 47% of brain energy, while the post-synaptic compartment consumes 34% [27]. In primates, the cortex is much thicker, reflecting increased complexities of signal processing, with functional MRI studies revealing that 75% of brain energy usage is post-synaptic [28]. Given the much greater efficiency of oxidative phosphorylation in extracting ATP from glucose, it is not surprising that neurons rely predominantly on mitochondrial respiration to sustain health and function [29,30]. As discussed in Part II, deficits in mitochondrial quality control have been linked to numerous neurological and neurodegenerative disorders.

Since the earliest descriptions of “focal cytoplasmic degradation” in the pathology literature [31], which predate the term “autophagy,” it has been recognized that the autophagic system is able to selectively degrade mitochondria in response to differentiation and injury cues. Mechanisms of selective mitophagy can be divided into three major categories (Table 1), based on the interactions that mediate contact between the mitochondrion and the phagophore. During development or in response to chronic stress, mitophagy is mediated by transmembrane mitophagy receptors that are transcriptionally upregulated during differentiation or hypoxia [34,35]. These receptors exhibit MAP1LC3/LC3-interacting regions (LIRs), or Atg8-interacting motifs (AIMs), that are cytosolically exposed. In addition, the protein products of two genes linked to recessive Parkinson disease (PD), *PINK1* (PTEN induced kinase 1) and *PRKN* (parkin RBR E3 ubiquitin protein ligase), mediate the ubiquitination of depolarized mitochondria for clearance [42]. Briefly, severe mitochondrial depolarization stabilizes PINK1 on the outer mitochondrial membrane (OMM) where it can undergo autophosphorylation, acting to phosphorylate ubiquitin at serine 65 (p-S65-Ub) as well as activating PRKN via phosphorylation of its serine 65 residue [38]; PRKN translocates to the mitochondrion and functions as an E3-ubiquitin ligase to tag OMM proteins with ubiquitin chains; PINK1 may also phosphorylate these chains. This mechanism involves bifunctional receptors with both ubiquitin binding and LIR domains [43], in analogy to mechanisms involved in the autophagic clearance of protein aggregates. Mitochondria may also be ubiquitinated by the mitochondrial ubiquitin ligase MARCHF5/MARCH5 [39]. In neurons and other cells, impaired mitochondrial respiration triggers enzymatic translocation of cardiolipin, a phospholipid essential for proper respiratory complex function, to the mitochondrial surface, where it interacts directly with MAP1LC3 to mediate mitophagy [22]. Interestingly, electron tomography studies show that these different mechanisms are reflected in the measured distances between the mitochondrial surface and the growing autophagic membrane, with receptor-mediated mitophagy, and presumably cardiolipin-mediated mitophagy, showing a tighter relationship than ubiquitin-mediated mitophagy [44].

**Table 1.** Summary of mitophagy mechanisms.

	Receptor-mediated		Ubiquitin-mediated		Lipid-mediated	
Cargo recognition	BNIP3L/NIX (BCL2 interacting protein 3 like)	FUNDC1 [32]	Ubiquitin/p-S65-Ub	Ubiquitin	Cardiolipin [22]	C18-ceramide [33]
Classic stimuli	Reticulocyte maturation, lens differentiation [34,35]	Hypoxia	CCCP/FCCP, mt-KillerRed, antimycin A	USP14 inhibitor	Rotenone, 6-OHDA, staurosporine	C18-ceramide; ceramide synthase 1 over-expression; Na selenite, cis-platin
Key regulators	HIF1 [36]	PGAM5, ULK1 [37]	PINK1, PRKN [38]	MARCHF5/MITOL [39]	PLSCR3, NME4 [22,40]	
Mechanism	Transcriptionally upregulated BNIP3L binds Atg8-family proteins (MAP1LC3B, GABARAPL2/GATE-16) via its LIR domain.	Dephosphorylation of FUNDC1 by PGAM5 at S13 unmasks its LIR domain. Phosphorylation at S17 by ULK1 promotes cargo recognition.	Mitochondrial depolarization impairs PINK1 import. PINK1 phosphorylates Ub and PRKN, leading to mitochondrial ubiquitination. Bifunctional adaptor proteins link ubiquitin to MAP1LC3	Activation of MARCHF5 leads to mitochondrial ubiquitination. Bifunctional adaptor proteins (OPTN, CALCOCO2, NBR1, SQSTM1, TAX1BP1) link ubiquitin to MAP1LC3	Cardiolipin is redistributed from the IMM, where it interacts with respiratory complexes, to the OMM. Cardiolipin binds MAP1LC3 via R10, R11 with additional hydrophobic interactions	Ceramide inhibits mitochondrial oxygen consumption and binds MAP1LC3 via F52 and I35
Regulatory switches	These BH3 proteins also function in apoptosis. Phosphorylation of BNIP3 promotes mitophagy vs. apoptosis decision		PINK1 regulates complex I, mitochondrial Ca^2+^ and dendritic complexity through distinct targets/mechanisms. Mitochondrial membrane potential collapse regulates mitophagy vs. other functions.		Cardiolipin-rich microdomains mediate optimal respiratory complex assembly. Cardiolipin peroxidation may regulate apoptosis vs. mitophagy decision	
Inhibitory mechanism		SRC and CSNK2/CK2 mediated FUNDC1 phosphorylation; BCL2L1/Bcl-XL inhibits PGAM5	Deubiquitinating enzymes	USP14		
Crosstalk	HIF1 also upregulates BNIP3, a BH3 protein that liberates BECN1	Degradation of the protein product of the long splice isoform of *BCL2L1/Bcl-xL* liberates BECN1	Degradation of MFNs and RHOT/miro facilitate mitochondrial sequestration		CL fatty acyl chain remodeling by TAFAZZIN/TAZ is involved in mitophagosome formation [41]	Results in mitophagic cell death
	BNIP3L/Nix may promote mitochondrial PRKN, act to rescue PRKN- and PINK1- patient fibroblasts	Pgam5 may regulate Pink1 functions in *Drosophila*			PHBs (prohibitins) are recruited to cardiolipin-rich microdomains and may play a role in both pathways	
Neurobiology	Not yet reported in neurons.	Not yet reported in neurons	Occurs in neurons, but to a lesser extent than in glycolytic cells. Can be triggered by focused mitochondrial damage in axons. OPTN is required in neurons.	Reported in iNeurons	Reported in cortical neurons. Also observed in lung epithelial and HeLa cells.	Not yet reported in neurons

Depending upon the nature of the stimulus, cargo or cell type, triggers for selective autophagy may recruit and activate the autophagy-initiating machinery through different pathways. Mitophagy induced by mitochondrial photoirradiation or complex I inhibition are mediated by mitogen-activated protein kinases, and do not require BECN1 [19,21]. Transmembrane mitophagy receptors such as BNIP3/NIX can directly recruit WIPI-ATG13 complexes, whereas FUNDC1 exclusively recruits ULK1 [45]. In depolarization-induced mitophagy, PRKN is not necessary for autophagy induction; rather BNIP3L/NIX inhibits MTOR signaling to activate canonical mitophagy [46]. PINK1-PRKN-dependent and a subset of PINK1-PRKN-independent mitophagy mechanisms are summarized in Table 1, and reviewed in depth elsewhere [17,47].

#### Basics of neuron structure and function

**Figure 2.**
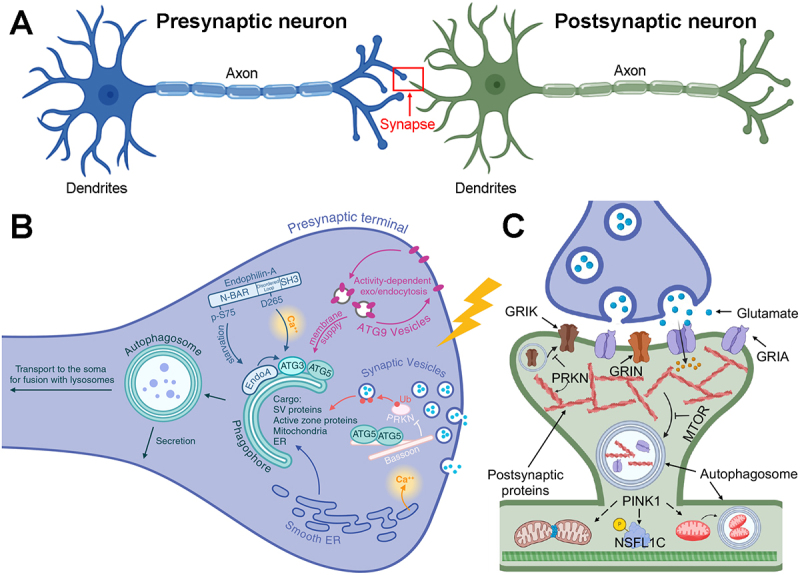
Autophagy in pre- and postsynaptic structures and synaptic plasticity. (A) Schematic representation of presynaptic and postsynaptic neurons. Multiple dendrites and typically a single axon project from the cell body/soma of each neuron. The red box indicates a synapse formed from contact between the presynaptic terminal of the blue neuron with spines (not shown) projecting from dendrites of the postsynaptic neuron (green). (B) Proteins enriched in presynaptic terminals regulate autophagosome formation in a manner that can be coordinated with synaptic activity. For example, availability of ATG9, which supplies membrane for the nascent autophagosome, is coupled with the synaptic vesicle cycle. Autophagosomes engulf a diverse array of substrates which can impact the synaptic vesicle pool and neurotransmission. Autophagosomes can then be routed to the soma for lysosomal degradation or secreted from the presynaptic terminal. (Ub; ubiquitin). (C) Autophagy plays a crucial role in dendritic spine elimination and modulates synaptic plasticity by sequestering and degrading postsynaptic receptor subunits and cytoskeletal proteins. Glutamate receptors that respond to kainate (GRIK), NMDA (GRIN) and AMPA (GRIA) are shown. PRKN interacts with postsynaptic proteins and regulates degradation of glutamate receptor subunits, inhibiting GRIK/kainate receptor-dependent excitotoxicity. PINK1 cooperates with PRKN to regulate mitophagy. PINK1 also regulates spine maturation and synaptic plasticity through non-mitophagy mechanisms involving mitochondrial fission and promoting phosphorylation of NSFL1C/p47. Figure assembled using Adobe Photoshop 22.4.3 and BioRender. Figure 2A,C: Created in BioRender. Lu, J and Chu, C. (2025) https://BioRender.com/mef6gn9; Figure 2B: Created in BioRender. Maday, S. (2025) https://BioRender.com/3zc2syo.

Neurons are highly polarized cells, with each compartment serving distinct functions (Figure 2). The neuronal soma consists of compact, rounded or pyramidal cytoplasm surrounding the nucleus. The soma is responsible for orchestrating the synthesis and turnover of all neuronal constituents. Depending upon the type of neuron, a variable number of elongated, hair-like cytoplasmic processes project from the contours of the soma. These result in morphologies ranging from pseudounipolar neurons to the much more common multipolar neurons. A pseudounipolar neuron exhibits a single process extending from the soma that splits to form axonal and dendritic processes that mediate outgoing and incoming signals, respectively. A multipolar neuron typically has a single long axon that projects up to a meter, with branches terminating in synaptic boutons or axon terminals from which neurotransmitters are released to affect the target neuron, muscle or gland cell (Figure 2A). In addition, the multipolar neuron supports an elaborate dendritic arbor originating from a variable number of primary dendrites. The dendritic arbor receives and integrates incoming signals from numerous synaptic contacts with the axon terminals of other neurons. In cortical pyramidal neurons, there is typically a thicker apical dendrite with numerous branches that extend toward the cortical surface, and a variable number of basal dendrites, which also branch extensively. In excitatory circuits, each dendritic branch or shaft is tightly packed with postsynaptic dendritic spines, which form synapses with presynaptic axonal swellings called boutons.

The entry and exit of proteins, nucleic acids, and organelles (such as synaptic vesicles and mitochondria) from the soma into the axon is regulated by molecular interactions in the axon initial segment [48]. Likewise, although the soma and dendritic compartments are often lumped together, specific mechanisms exist to promote or exclude cargos from entering the dendritic arbor. While most studies have concentrated on axonal trafficking, emerging knowledge suggests that distinct mechanisms govern dendritic transport [49].

A key question is whether autophagosomes in different neuronal compartments exhibit unique characteristics and regulatory mechanisms. Recent work indicates that during inhibitory avoidance learning, proteins involved in autophagy and lysosomal degradation are significant upregulated, primarily in neurons throughout the hippocampus and its subregions [50]. Notably, the autophagy and lysosomal proteins BECN1, MAP1LC3B and LAMP1 were elevated in both the soma and processes of neurons, whereas SQSTM1 increased only in the soma [50]. Additionally, SQSTM1 and LAMP1 showed higher levels in astrocytes and microglia [50]. The differential increase of these proteins in different cell types and compartments suggests a specific and compartmentalized regulation of autophagy-lysosomal activity in the brain during learning. Additional insights into the compartmentalization of autophagy within neurons have been provided by recent studies in primary neurons [51,52]. Autophagosomes originating in the axon quickly begin to mature, as indicated by the presence of the late endosome/lysosome marker LAMP1, and exhibit greater mobility compared to those derived from the somatodendritic compartment [52]. Interestingly, synaptic activity regulates the dynamics of autophagic organelles within neurons, particularly in dendrites as compared with axons [51]. Increased synaptic activity reduces the motility of autophagic organelles in dendrites and stimulates their maturation into degradative compartments, suggesting that these organelles are retained near active synaptic regions for localized degradation [51]. These findings suggest that the functional and structural compartmentalization observed in neurons may create molecularly distinct pools of autophagosomes, each tailored to meet specific functional demands. This compartmentalization contributes to the overall regulation of neuronal health and synaptic function.

### Fundamental pathway for autophagy in axons

In the axon, autophagy captures cargo in the distal axon and presynaptic sites for delivery to the soma for degradation. Live-cell imaging, including of fluorescently-tagged MAP1LC3, reveals a concentration of autophagosomes formed in axonal growth cones and presynaptic sites [53–57]. Following formation, autophagosomes undergo directed dynein-mediated transport to the soma [53,54,58–60]. The journey back to the soma is initiated by fusion with LAMP1-positive organelles that originate from the soma and may act to supply components to facilitate organelle maturation *en route* (reviewed in [61]). Delivery to the soma ensures full maturation into degradative autolysosomes given that the soma is rich with proteolytically-active and mature lysosomes [62–65]. In fact, neutralizing lysosomal function to block degradation results in an accumulation of autophagosomes largely in the soma [52].

Much of the mechanisms driving this retrograde pathway for axonal autophagy has been elucidated in primary neurons [61]. While these models may not fully capture the complexity of the neuronal microenvironment *in vivo*, including contributions from glial cells, systemic factors, and aging, *in vitro* models remain invaluable for foundational discoveries, particularly in relation to intrinsic neuron-autonomous mechanisms, and can guide investigations within more complex physiological conditions. Indeed, this axonal autophagy pathway is conserved in intact nervous systems *in vivo* across various model systems [66–69]. A recent *in vivo* study used two-photon imaging in the rat central nervous system to show robust retrograde transport of autophagosomes in the optic nerve [70]. Thus, a retrograde pathway for axonal autophagy represents a fundamental pathway for axonal homeostasis. Interestingly, several studies find that these retrograde axonal autophagosomes are distinct from organelles carrying newly-endocytosed cargo [69,71]. Both axonal autophagosomes and newly-formed endosomes undergo retrograde transport from the distal axon to the soma, but as separate organelle populations that differ in their degree of acidification and maturation state [71]. Segregating these pathways might serve to protect endocytic cargoes that carry signaling information from precocious autophagic degradation [71].

#### Coupling of presynaptic autophagy with synaptic activity

Proper synaptic function demands active remodeling of the proteome in response to activity-based cues. Moreover, synaptic activity can create heavy demands on the local proteome, which can lead to damage. Thus, maintaining proteostasis at the synapse requires robust quality control pathways. Indeed, synaptic activity can stimulate the formation of autophagosomes in presynaptic terminals [57,66,72,73]. Moreover, reactive oxygen species (ROS)-induced damage to synaptic vesicle proteins is sufficient to rapidly stimulate autophagosome production in the presynaptic terminal [74]. These findings suggest a critical role for autophagy in regulating the synaptic proteome in response to activity or local damage.

But how is activity-based information decoded to generate an autophagosome? Local formation of autophagosomes in the distal axon involves core autophagy proteins conserved from yeast [54,75]. Interestingly, this process is regulated by proteins enriched in presynaptic terminals, including molecules involved in synaptic vesicle endocytosis [57,73], synaptic vesicle trafficking [76], and active zone proteins [77,78]. In *Drosophila*, Endophilin-A (EndoA; human homolog SH3GL2/endophilin A1), a BAR-domain protein required for synaptic vesicle endocytosis, may distinguish different signals that induce autophagy in presynaptic terminals, such as activity-induced calcium influx from metabolic stress [57] (Figure 2B). EndoA generates highly curved membranes that recruit the autophagy protein ATG3 to drive autophagosome formation [57]. This process is stimulated by phosphorylation of serine 75 in the amphipathic helix H1 of EndoA, which based on experiments with mammalian SH3GL2/endophilin A1 [79], is predicted to promote a shallower insertion of EndoA into membranes to generate curvature [57]. In fact, a non-phosphorylatable S75A mutant of EndoA blocks autophagosome formation induced by starvation [57]. A follow-up study identifies a residue (D265) in the disordered loop between the BAR and SH3 domains of EndoA that regulates EndoA flexibility and autophagosome formation in response to calcium influx, but not starvation; this mechanism is distinct from phosphorylation of helix H1 [73]. Thus, starvation and activity-induced calcium influx can promote autophagosome formation in presynaptic terminals via distinct mechanisms. Future studies will need to elucidate how different autophagy stimuli are coordinated with cargo selection.

The rate of autophagosome formation in the presynaptic terminal may be defined by the synaptic vesicle cycle in controlling the available pool of ATG9 (Figure 2B). ATG9 is a transmembrane lipid scramblase that drives autophagosome membrane expansion [80,81] (Figure 1B). It does so by flipping phospholipids, derived from a membrane source by ATG2 [82], between outer and inner leaflets of the phagophore membrane [80,81]. Indeed, depleting the pool of ATG9 in presynaptic terminals reduces autophagosome biogenesis in the axon [55,83,84]. ATG9-positive vesicles are distinct from synaptic vesicles [78,85,86], but they undergo cycles of endo/exocytosis that are coupled to synaptic activity and depend on the machinery that mediates synaptic vesicle cycling [e.g., the *C. elegans* DYN-1/dynamin 1/DNM1 homolog, UNC-26/synaptojanin 1/SYNJ1 homolog, and UNC-57/endophilin A/ SH3GL (1-3) homolog] [72]. Additionally, the *C. elegans* active zone protein CLA-1/clarinet may bridge exocytic active zones with endocytic periactive zones to regulate ATG9 sorting at synapses [78]. In this way, the neuron may coordinate autophagosome formation in presynaptic terminals with its activity state.

#### Functions for presynaptic autophagy

During development of the nervous system, neurons need to extend their axons to reach their correct targets to form functional synaptic connections [87]. Loss of autophagy genes can lead to defects in axon outgrowth and guidance, causing dysgenesis of interhemispheric tracts (bundles of axons that connect left and right hemispheres) in the murine brain [55,88–91]. In fact, a common pathology in congenital disorders of autophagy is a thinning of the corpus callosum [92]. Interestingly, BDNF (brain-derived neurotrophic factor), a growth factor in the central nervous system that supports neuronal development and survival, is an important regulator of neuronal autophagy [93–96]. BDNF can stimulate autophagy in axons [93,94]. Loss of autophagy in developing *Drosophila* photoreceptors leads to the formation of supernumerary synapses with aberrant neuronal partners [97]. In this model, autophagy may function to destabilize unfavored axonal filopodia (structures that survey synaptic partners) by degrading factors that promote assembly of the active zone (e.g., Liprin- and RhoGAP100F/Syd-1) [97]. Thus, autophagy functions early in synaptogenesis to restrict inappropriate synaptic connections. In another neuronal subtype in the fly visual system, the dorsal cluster neurons, autophagy may need to be suppressed during later stages of synaptogenesis to prevent aberrant degradation of active zone proteins and loss of mature synapses [98]. In short, the roles for autophagy in the axon are dynamic and depend on neuronal subtype and context, and involve the degradation of different cargoes. Further evidence of neuron subtype-specific roles for autophagy has also been revealed in *C. elegans* deficient for *atg9* [55]. This study showed that autophagy is required for early stages in axonal outgrowth of nociceptive sensory PVD neurons and for the organization of presynaptic terminals in AIY interneurons. In total, compelling emerging evidence indicates key roles for autophagy in establishing proper connectivity of the nervous system.

Knockout of key autophagy genes can also lead to alterations in synaptic function. Indeed, deletion of *Atg5* in excitatory neurons in the cortex and hippocampus increases presynaptic neurotransmission [99]. What is the mechanism? Several groups find that neurons deficient for key autophagy genes (e.g., *Atg5* or *Atg7*) exhibit an accumulation of tubular endoplasmic reticulum (ER), but only in axons and not in dendrites [99,100]. This accumulation of ER can lead to elevated calcium release from the ER via ryanodine receptors, triggering secretion of synaptic vesicles [99]. Thus, a key function for axonal autophagy is to maintain axonal ER and synaptic calcium homeostasis to prevent aberrant neurotransmission (Figure 2B).

Autophagy may also constrain presynaptic neurotransmission by regulating the synaptic vesicle pool. Pharmacological stimulation of autophagy can dampen the synaptic vesicle pool size [101,102]. Conversely, loss of autophagy elicits an increase in synaptic vesicle release probability [102] and evoked neurotransmitter release [101].

Are synaptic vesicles or their components substrates for autophagy? Synaptic vesicle proteins are present in a subset of autophagosomes as assessed using immunofluorescence [102]. Another study finds that deletion of BSN/Bassoon, a presynaptic scaffolding protein of the active zone that also functions as a negative regulator of autophagy by sequestering ATG5 [77], increases the colocalization of synaptic vesicle proteins with autophagosomes and decreases the synaptic vesicle pool size [103] (Figure 2B). Bassoon negatively regulates PRKN-dependent ubiquitination of synaptic vesicle proteins, thereby controlling their rate of clearance by autophagy [103]. Insights gleaned from proteomic analysis of autophagic organelles isolated from mouse brain have identified presynaptic content that includes synaptic vesicle proteins, proteins involved in synaptic vesicle trafficking, and components of the active zone [104,105]. Interestingly, synaptic cargos are enriched in adult and aged mice [104]. Autophagy can also selectively degrade individual synaptic vesicle proteins that are focally damaged [74], rather than degrading the entire synaptic vesicle. Failure to remove damaged synaptic vesicle proteins by autophagy dampened excitatory postsynaptic current amplitudes [74], suggesting important roles for autophagy in protecting the integrity of the synaptic vesicle proteome for proper neurotransmission. However, other reports in autophagy-deficient neurons do not find alterations in synaptic vesicle number [100] or bulk levels of synaptic vesicle proteins [99,106]. Future studies are needed to elucidate how different autophagy stimuli are coordinated with cargo selection.

An intriguing possibility is whether autophagosomes might be secreted from presynaptic terminals. Proteins that affect synaptic development versus activity-induced synaptic remodeling of boutons at the neuromuscular junction were identified using a comparative RNAi screen in *Drosophila* [107]. Knockdown of core autophagy proteins involved in autophagosome formation (e.g., Atg1 or Atg8) disrupted both synapse formation and activity-induced synaptic remodeling [107]. Interestingly, proteins that selectively disrupted synapse development were highly enriched in the lysosomal pathway and included lysosomal degradative enzymes and lysosomal membrane proteins [107]. By contrast, proteins that selectively disrupted activity-induced synaptic remodeling were enriched for modulators of autophagy activation rather than degradation [107]. In fact, neuronal activity stimulated the production of autophagosomes in synaptic boutons but decreased their colocalization with lysosomes [107], suggesting a detour from the canonical fate of degradation. Proteins that disrupted activity-induced synaptic remodeling also included Snap29 [107], a SNARE implicated in secretory autophagy [108,109]. Presynaptic knockdown of the machinery involved in secretory autophagy (e.g., Sec22 and Snap29) impaired activity-based synaptic remodeling but not synaptic development [107]. Inhibiting autophagosome secretion prevented activity-induced increases in bouton size and postsynaptic AMPA receptor GluR (human homolog GRIA) levels, altering synaptic plasticity [107]. These data suggest that autophagosomes may have distinct fates, degradative versus secretory, and synaptic activity may drive autophagosomes toward the secretory route. This outcome, however, may be dependent on the type and duration of the activity stimulus as more sustained activity seems to drive degradative functions, as discussed above.

What might be the functional advantage of secreting autophagosomes? Secretory autophagosomes may provide membrane to enable synapse enlargement in response to activity. It has been proposed that cellular stress stimulates secretory autophagy to release factors that promote maturation of BDNF [110]. Secretory autophagy may also provide an alternative mechanism to dispose of cellular waste, particularly in the context of lysosomal dysfunction [111–113]. It will be interesting to further resolve the function of secretory autophagosomes at synapses moving forward.

### Postsynaptic autophagy and dendritic spines

#### Autophagy and spine pruning

The regulation of synaptic growth and plasticity is critical for the proper development of neural circuits that govern behavior and their ability to adapt to experiences and environmental changes in processes related to learning and memory. Postnatal synaptic development is a dynamic process that involves both the formation and the elimination or pruning of synapses [114,115]. These dynamic changes are crucial for the selection and maturation of synapses and neural circuits. While relatively little is known about the underlying mechanisms by which neuronal autophagy influences synaptic structure, earlier studies in *Drosophila* demonstrate that a developmental loss of autophagy reduces the size of the neuromuscular junction. Conversely, enhancing neuronal autophagy by overexpression of Atg1, a homolog of ULK1, results in increased numbers of synaptic sites [116]. However, in the mammalian brain, autophagy is inversely correlated with synaptic numbers. One study suggests that autophagy is essential for developmental pruning of dendritic spines, a process that appears to be impaired in individuals with autism spectrum disorder (ASD) [11]. The authors observed an elevated density of dendritic spines, increased levels of DLG4/PSD95 (discs large MAGUK scaffold protein 4), and reduced developmental pruning of these spines in layer V pyramidal neurons within the temporal lobe of postmortem ASD brains. Interestingly, dendritic spine pruning deficits in ASD correlates with hyperactivated MTOR and impaired autophagy, with a statistically significant increase in phosphorylated MTOR in the brains of ASD patients compared to age-matched controls [11]. Consistently, ASD patients showed significantly lower levels of MAP1LC3-II, an autophagosome marker, throughout childhood and adolescence [11]. Given that MTOR activation suppresses autophagy, this heightened MTOR activity suggests a reduction in autophagy levels [117]. Additionally, transgenic mice with hyperactivated MTOR displayed ASD-like behaviors and impaired spine pruning in cortical projection neurons, which were corrected by administering the MTOR inhibitor rapamycin [11]. Collectively, these studies emphasize a critical role for autophagy in synaptic pruning and suggest that disruptions in autophagy may be linked to ASD phenotypes.

The mitophagy protein PRKN, which is associated with autosomal recessive PD, is also involved in regulating post-synaptic pruning [12] (Figure 2C). Knockdown of PRKN resulting in a proliferation of glutamatergic synapses and increased vulnerability to excitotoxic injuries, indicating that one physiologic role for PRKN involves downregulating excitatory synapses. Interestingly, PRKN interacts with the post-synaptic density and regulates the degradation of glutamate receptor subunits (Figure 2C) [118,119]. It also promotes degradation of SYT11 (synaptotagmin 11), a putative PD risk factor [120]. SYT11 not only regulates the presynaptic vesicle recycling pathway, but also regulates anterograde and retrograde trafficking of distinct vesicles and their distributions throughout the soma, axon and dendrites of neurons. PRKN interacts with SH3GL/endophilin-A in a phosphorylation-sensitive manner to ubiquitinate synaptic protein complexes [121]. However, the relative roles of proteasomal versus autophagic degradation in relation to synaptic regulation downstream of PRKN would need to be experimentally determined.

#### Autophagy and synaptic plasticity

Synaptic plasticity is defined as the activity-dependent strengthening or weakening of synaptic transmission over time, primarily through modifications to postsynaptic receptors on neurons. These include glutamate receptors that bind N-methyl-D-aspartate (NMDA, composed of GRIN/GluN subunits), alpha-amino-3-hydroxy-5-methyl-4-isoxazolepropionic acid (AMPA, composed of GRIA/GluA subunits), and kainate (composed of GRIK/GluR subunits) (Figure 2C). The ability of autophagy to degrade postsynaptic scaffolding proteins provides another potential mechanism by which it can regulate synapse structure and influence the efficacy of synaptic transmission (Figure 2C). During synaptogenesis, when presynaptic terminals engage with the postsynaptic cell, the postsynaptic cell upregulates local protein synthesis, accompanied by clustering and anchoring of neurotransmitter receptors and scaffolding molecules at the postsynaptic membranes [122–124]. Following patterned neuronal activity, postsynaptic proteins are either incorporated into or removed from the synapse, leading to enduring changes in synaptic strength [123,124].

Previous research involving *C. elegans* show that autophagy also facilitates the degradation of inhibitory receptors from postsynaptic and non-synaptic regions. The neurotransmitter gamma-aminobutyric acid (GABA) mediates inhibitory neurotransmission through ligation of fast-acting inotropic GABAA receptors and G-protein coupled GABAB receptors. In muscle cells, the clustering of postsynaptic GABAA receptors relies on presynaptic input [125]. GABAA receptors on postsynaptic spines that fail to receive input from GABAergic terminals are internalized for autophagic degradation [126]. Notably, this autophagic degradation is selective to GABAA receptors, with acetylcholine receptors in the same cells being spared [126]. These data indicate that transmembrane postsynaptic receptors can be selectively degraded via autophagy in an endocytosis-dependent manner.

In addition to involvement in developmental synaptogenesis, autophagy pathways are triggered by synaptic activity to selectively degrade postsynaptic proteins, thereby modulating the long-term synaptic remodeling necessary for learning and memory. Either high potassium stimulation or chemically induced long-term depression (LTD) briefly increases the number of autophagosomes and the levels of autophagy-related proteins in dendritic shafts and spines of rat hippocampal neurons [127]. Furthermore, the levels of the AMPA receptor subunit GRIA1/GluR1 decreased following chemical LTD, with this degradation being partially reduced by autophagy inhibitors [127]. These findings indicate that postsynaptic autophagy, regulated by neuronal activity, is at least partially responsible for the receptor degradation required for maintenance of LTD. Later research provides further evidence that autophagy modulates synaptic biology by directly degrading synaptic proteins [95]. In the absence of autophagy in cortical pyramidal neurons, levels of three prominent scaffold proteins that are essential for dendritic spine remodeling are elevated: DLG4/PSD95, PICK1, and SHANK3 [95]. By isolating and purifying autophagosomes from the mouse brain, this study showed that synaptic proteins are present inside autophagosomes and serve as direct substrates of autophagy [95].

#### Mitochondria, PINK1, and post-synaptic biology

Synapses serve as crucial communication sites between neurons and require significant energy to support essential molecular and cellular processes [128]. Mitochondria are vital organelles at these sites, providing the energy to establish synaptic circuitry and promote synaptic plasticity [128,129]. Mitophagy is a form of selective autophagy involved in the removal of damaged mitochondria [47,130]. PINK1 is a serine/threonine kinase that has been extensively explored for its roles in regulating mitochondrial respiration, fission-fusion, mitophagy, and transport [47,130–132]. Interestingly, significant cognitive impairments are frequently observed among human PINK1 mutation carriers [133–136], and PINK1 plays an important role in promoting dendritic branching, synaptic spine density *in vitro* and *in vivo*, and spine maturation [13,137].

When mitochondria are depolarized, PINK1 accumulates on the outer mitochondrial membrane, and upon autophosphorylation, recruits the E3 ubiquitin ligase PRKN [47,130]. PRKN ubiquitinates outer mitochondrial membrane proteins, tagging the mitochondria for degradation [47,130]. Several earlier studies [138,139] suggest that deficient mitophagy may contribute to synaptic dysfunction. However, more recent studies show that mice lacking PINK1 or PRKN do not spontaneously develop any overt phenotype, and the basal levels of mitophagy in the brain remain unchanged despite the absence of these proteins [140]. In contrast, PINK1 and PRKN are essential for hypoxia-induced mitophagy in larval wing disc tissues [141], indicating tissue/cell type specificity. Primary neurons exhibit diminished PRKN-mediated mitophagy responses compared to proliferative tumor cells [142,143]. Moreover, both *in vitro* and *in vivo* studies reveal that neurons engage in PINK1-PRKN-independent mitophagy (e.g., the cardiolipin and MARCHF5 pathways) [22,39,140,144] (Table 1). A developmental absence of the autophagy protein ATG5 causes only a transient delay in the acquisition of normal hippocampal spine densities [145]. In contrast, *pink1* knockout elicits persisting spine deficits in both embryonic and adult mouse neurons at 6 months of age [13], implicating additional mechanisms. There are also mitophagy-independent pathways to remove mitochondria from axons [146]. Given these observations, and incomplete knowledge concerning the spectrum of mitophagy pathways engaged in primary neurons, the roles of specific mitophagy pathways in regulating post-synaptic health and function remain unclear.

What other mechanisms might be engaged by PINK1 and/or PRKN in support of synaptic health and function? PINK1 and PRKN work together not only in mitophagy, but also in regulating mitochondrial biogenesis [147–149]. Both proteins regulate mitochondrial dynamics, albeit through different mechanisms [150–152]. PRKN localizes to the postsynaptic density, where it mediates degradation of receptors [118,119] (Figure 2C). It also regulates vesicular transport or recycling by suppressing SYT11 expression [120]. PINK1 regulates post-synaptic mitochondrial calcium fluxes [153] and participates in a cytosolic pathway involving NSFL1C/p47 to promote dendritogenesis [137] and the maturation of dendritic mushroom spines [13].

Mitochondria undergo dynamic morphological changes and actively traffic within neurons. Changes in mitochondrial morphology and distribution regulate energy supply, calcium homeostasis, and other fundamental aspects of neuron structure and physiology [129]. Dendritic mitochondrial dynamics contribute to the induction of GRIN/NMDA receptor-dependent long-term potentiation (LTP) [154], the maintenance of long-term plasticity [155], and the facilitation of plasticity-induced local protein translation [156]. In particular, a burst of mitochondrial fission is required for LTP [154]. Interestingly, PINK1 can phosphorylate DNM1L/Drp1 at S616 to promote mitochondrial fission [157]. While it may be tempting to conclude that PINK1 supports synaptic development and plasticity by promoting mitochondrial fission (Figure 2C) [150,158,159], in mammalian neurons, PINK1 also acts to promote mitochondrial elongation [151]. Moreover, recruitment of endogenous PRKA/PKA to mitochondria rescues multiple PINK1-deficient phenotypes in primary neurons through phosphorylation of DNM1L/Drp1 at a site that suppresses fission (S656-rat; S637-human) [160]. Fission related to mitophagy or cell death is regulated differently from fission related to mitochondrial proliferation [161]. The regulation of dynamic mitochondrial fusion-fission cycles in the setting of synaptic activity, and the potential role of PINK1, represent areas for future study.

In axons, PINK1 phosphorylates Miro1, an outer mitochondrial membrane-associated Rho GTPase involved in mitochondrial transport. Depending upon the site of PINK1-linked phosphorylation, RHOT1/MIRO1 is either stabilized or undergoes PRKN-dependent proteasomal degradation. Stabilization is mediated by PINK1-linked phosphorylation at threonine 298/299, while degradation is mediated by phosphorylation of serine 156 [162,163]. Given the essential role of mitochondria in providing energy and buffering calcium at excitatory synapses [164,165], PINK1 may stabilize mitochondria in specific areas with high energy requirements to support synaptic development, while also acting to promote mitochondrial transport into dendrites [132,166].

Another mechanism by which PINK1 regulates synaptic mitochondria is through its ability to regulate mitochondrial calcium handling. Synaptic activity causes massive cytosolic calcium fluxes, buffered by rapid mitochondrial calcium uptake through the mitochondrial calcium uniporter, followed by a gradual release back to the cytosol. The proper release of mitochondrial calcium is essential for some forms of synaptic potentiation [167], and deficient release has been implicated in mitochondrial calcium overload [2,168]. An early study showed that PINK1 loss of function impairs the release of mitochondrial calcium to the cytosol [169]. Subsequently, a novel phosphorylation site of the mitochondrial calcium antiporter SLC8B1/NCLX was discovered, which is regulated by PINK1 via PRKA/PKA [153]. An SLC8B1^S258D^ phosphomimic completely rescues defective mitochondrial calcium release in dopaminergic neurons from *pink1* knockout mice and protected them from dopamine-induced cell death, indicating the importance of proper mitochondrial calcium handling in neurons [153].

Mitochondrial calcium overload triggers the selective loss of post-synaptic mitochondria via mitophagy during excitatory stress elicited by mutations in LRRK2 (leucine rich repeat kinase 2) [170,171]. Enhancing mitochondrial calcium egress by mimicking the PINK1-regulated SLC8B1/NCLX phosphorylation at serine 258 confers protection against postsynaptic degeneration in this model. While the exact role of calcium-regulated mitophagy and altered mitochondrial dynamics remain to be fully elucidated in relation to excitatory neurotransmission [2], it is clear that enhancing PINK1 expression improves structural and functional parameters implicated in learning and memory [137,172–174].

PINK1 primarily localizes with mitochondria, but is also released into the cytosol following processing by mitochondrial peptidases for signaling or clearance [151,175,176]. Recently, cytosolic PINK1 was found to mediate mitophagy-independent signaling pathways important for maintaining the dendritic arbor [166,177]. The loss of endogenous PINK1 leads to dendritic simplification and synaptic deficits in cortical, hippocampal and midbrain neurons [13,150,166]. Conversely, upregulation of PINK1 promotes dendritic morphogenesis and synaptic maturation [137,174]. To determine which subcellular pools of PINK1 are responsible for promoting neurite extension, cells were transfected with two distinct PINK1 constructs: one that targeted PINK1 to the outer mitochondrial membrane (OMM-PINK1) and another that restricted PINK1 to the cytosol (ΔN111-PINK1) [166]. Both constructs prevented cell death due to the loss of endogenous PINK1. However, transfection with the cytosolic form (ΔN111-PINK1), but not the mitochondrial form (OMM-PINK1), significantly promoted dendritic growth in primary neurons [166].

How does cytosolic PINK1 regulate dendritic remodeling? Recent studies indicate that PINK1 interacts with valosin-containing protein (VCP) [137,178]. In the cytosol, this interaction scaffolds the activation of PRKA/PKA to phosphorylate NSFL1C/p47, a cofactor of valosin-containing protein that regulates membrane dynamics, at serine 176 [137]. A NSFL1C/p47^S176D^ phosphomimic effectively restores normal dendritic complexity and spine densities in neurons lacking PINK1 expression (Figure 2C) [13,137]. Interestingly, the yeast homolog of NSFL1C/p47, Shp1, is essential for autophagosome biogenesis, and co-expression of NSFL1C/p47 can ameliorate the autophagic blockage caused by VCP mutations [179,180]. The potential role of PINK1-regulated NSFL1C/p47 phosphorylation on autophagy, and the relative importance of autophagic versus non-autophagic mechanisms in mediating the effects of PINK1 on spine structure and function remain to be defined through future studies.

### Synaptic regulation by autophagy and mitophagy pathways

#### Neuronal autophagy and learning behaviors

As discussed above, autophagosomes are actively assembled at synapses and along neuronal processes in hippocampal neurons [181–183]. Further, mitophagy-linked proteins such as PINK1 and PRKN regulate several aspects of post-synaptic structure and function. The well-documented age-related decline in hippocampal-dependent memory is correlated with a reduction of autophagy proteins in the brain, wherein ATG5, ATG7, and BECN1 are downregulated [184–186]. These observations implicate a role for autophagy in learning and memory. What then is the impact of autophagy modulation on specific learning behaviors?

The involvement of autophagy in memory formation, retrieval, and learning has been extensively documented in several recent studies [187–189]. Pharmacological inhibition of autophagy by infusing spautin-1 in the hippocampus before water maze training impairs long-term memory retention in mice. Conversely, activation of autophagy with the Tat-Beclin1 activator peptide enhanced long-term memory [189]. Subsequently, inhibiting autophagy by viral-mediated knockdown of autophagy proteins RB1CC1/FIP200, BECN1, or ATG12, or hippocampal administration of spautin-1, impaired novel object recognition and contextual fear conditioning. In contrast, administration of the autophagy activator Tat-Beclin1 enhanced retention of both types of memories [188]. Intriguingly, autophagy activity declines in the hippocampus with aging. Fully restoring autophagy levels is both necessary and sufficient to reverse associative and spatial memory deficits in aged mice [188].

The role of autophagy in brain function has also been investigated through cell-type-specific knockouts of autophagy genes. Mice with conditional knockout of *Atg7* in the two main types of spiny projection neurons in the striatum illustrate a role for autophagy in uniquely regulating neuronal activity in different cell types involved in action selection and reinforcement learning [190]. Autophagy is essential for maintaining normal dendritic structure and synaptic input in spiny projection neurons of the direct pathway within the basal ganglia [190]. Interestingly, the loss of ATG7 in spiny projection neurons of the indirect pathway did not alter dendritic complexity, spine density, or excitatory inputs, but caused intrinsic hyperexcitability due to reduced Kir2 function [190]. Despite the differing cellular effects observed in neurons of the direct and indirect pathways, the loss of ATG7 in either neuron type leads to deficits in striatal-based behaviors [190]. This suggests that autophagy is crucial for both types of neurons in maintaining normal brain function and behavior, albeit through distinct mechanisms.

While autophagy clearly plays a role in memory formation, emerging evidence suggests that autophagy is not essential or beneficial for all aspects of learning and memory. For example, autophagic vesicles form locally in dendrites following stimuli that produce long-term depression (LTD) [14], a major form of long-lasting synaptic plasticity that supports cognitive function. These vesicles degrade GRIA/AMPAR subunits and other critical postsynaptic proteins. Unexpectedly, mice with conditional autophagy deficiency in excitatory neurons exhibited enhanced cognitive flexibility, outperforming controls in related behavioral tasks [14]. Similarly, while reduced autophagy impairs certain cognitive functions, preserved locomotion indicates that not all neural circuits are equally affected [191]. It is possible that too little or too much autophagy at the synapse, or the nature of the cargo being degraded, affects the outcome of autophagy modulation, just as the impact of autophagy on neuronal cell death or survival is context dependent [192]. The relationship of synaptic autophagy to synaptic plasticity, and the mechanisms by which autophagy is regulated in the context of learning and memory remain incompletely understood.

#### ATG9 and its role in synaptic function

ATG9 is an essential protein involved in autophagy, the only known mammalian ATG protein to have a transmembrane domain. ATG9 participates in the formation and transport of vesicles that are crucial for the assembly of the autophagy machinery. These vesicles serve as platforms for autophagosome formation, providing the necessary lipids and proteins for the expansion of the phagophore, the precursor to the autophagosome [193]. In mammals two homologs of ATG9 are expressed: ATG9A and ATG9B. *Atg9A*-deficient mice die during embryonic development [194] or within one day of birth with a similar phenotype as *Atg5-, Atg7-*, and *Atg16L1*-deficient mice [195]. Conditional knockout (CKO) specifically targeting the central nervous system (CNS), causes axon-specific lesions like those observed in *atg7-*CKO mice [91]. Additionally, *atg9a-*CKO mice showed growth retardation, indicating the critical role of ATG9A in neuronal health and development [91].

ATG9 vesicles are generated from the secretory pathway and navigate through various cellular compartments, including the ER, Golgi, trans-Golgi network, plasma membrane, and endosomal compartments. Using cells deficient for both ATG2A and B to generate a pre-ATG2 compartment that is enriched for ATG9 and MAP1LC3B-II, researchers find that upon autophagy induction, ATG9 vesicles are recruited to the phagophore assembly site (PAS) to serve as seed vesicles to initiate autophagosome biogenesis [81]. ATG9 possesses a lipid scramblase activity, which together with the lipid transferase ATG2 facilitates the exchange of phospholipids between the inner and outer leaflets of membranes [80,196]. This activity is vital for the expansion and maturation of the phagophore, as it helps in lipid mobilization from lipid droplets to phagophore membranes [197].

Much less is known about how ATG9 regulates autophagosome formation in neuronal cells [198]. ATG9-containing vesicles originate from the trans-Golgi network located in the soma and must travel to distal axons where autophagosome biogenesis is prominent. These vesicles are transported anterogradely along axons by kinesin motors, such as UNC-104/KIF1A, which are responsible for moving cargo toward the plus ends of microtubules [199]. Once at the synapse, ATG9-containing vesicles undergo activity-dependent exo-endocytosis, a process that is regulated by proteins such as UNC-13/Munc13 and UNC-26/Synaptojanin [72]. ATG9 couples synaptic exo-endocytosis to autophagy, thus linking synaptic autophagy to the activity state of the neuron. The authors propose that disruptions in endocytosis or autophagy could impair the clearance of damaged synaptic components, ultimately leading to synaptic dysfunction [72].

Very recent evidence using super-resolution microscopy and proteomic analyses reveal that ATG9 vesicles possess a unique protein composition, with low levels of traditional trafficking proteins such as coat proteins, tethering factors, and SNAREs [85,86]. In *C. elegans* neurons, the protein CLA-1/clarinet, particularly its long isoform (CLA-1 L), plays a crucial role in the active zone, the specialized, electron-dense region on the presynaptic terminal where synaptic vesicles dock, prime, and fuse to release neurotransmitters. CLA-1 regulates the sorting and trafficking of the autophagy protein ATG9 at synapses. This regulation is essential for presynaptic autophagy, helping to maintain synaptic protein homeostasis [78]. CLA-1 facilitates the sorting of ATG9-containing vesicles, ensuring they are properly trafficked to synapses where they can participate in autophagosome formation. This process is particularly important during increased neuronal activity, as it helps manage the demand for enhanced autophagy flux. Additionally, CLA-1 interacts with endocytic scaffolding proteins, linking exocytosis at the active zone with endocytosis at the periactive zone. This coordination is vital for the efficient cycling of synaptic components and the maintenance of synaptic function [78].

Overall, ATG9-containing vesicles are specialized for lipid delivery in autophagy, operating in parallel to, but distinct from, synaptic vesicle cycles. Their distinct composition suggests that ATG9 vesicles do not directly participate in the typical synaptic vesicle cycle but rather function as lipid shuttles distinct from synaptic vesicles. ATG9 vesicles scavenge lipids from intracellular compartments to support autophagosome formation, a process crucial for maintaining synaptic protein homeostasis and supporting neuronal function. The vesicles likely facilitate direct lipid transfer at synaptic sites, compensating for the limited ATG2-mediated lipid transfer seen in non-neuronal cells [198]. This specialized adaptation highlights the critical role of ATG9 in neuronal autophagy and synaptic maintenance. These studies also emphasize the heterogeneity in membrane composition of ATG9-containing vesicles in different cell types and cell compartments.

#### Glia/microglial autophagy, mitophagy and synaptic regulation

Microglia, the resident immune cells of the central nervous system, are involved in the surveillance and maintenance of synaptic environments. Recent evidence demonstrates loss of Atg9 in fly glial cells leads to reduced autophagic activity and accumulation of glial protein aggregates, accompanied by progressive dopaminergic neuron loss and locomotion deficits as seen in PD [200]. GAK (cyclin G associated kinase) is a multifunctional protein kinase involved in various cellular processes, including clathrin-mediated endocytosis and cell cycle regulation. In the context of PD, GAK has been identified as a genetic risk factor, with studies suggesting that its dysfunction may contribute to the pathogenesis of the disease. GAK is thought to influence PD by affecting the endocytic pathway within glia and potentially modulating the aggregation and clearance of SNCA/alpha-synuclein, a protein that forms toxic aggregates in the brains of individuals with PD. In the absence of GAK, the number and size of the autophagosomes and autophagosomal precursors increase in adult fly glia and mouse microglia. The Drosophila GAK homolog Aux contributes to PD-like symptoms including dopaminergic neurodegeneration and locomotor function in flies [201].

Autophagy in microglia is essential for synaptic pruning, a process critical for the refinement of neural circuits during development [202]. Impaired autophagy in microglia leads to defective synaptic pruning, resulting in increased dendritic spine density and synaptic dysfunction [203]. Microglia lacking the autophagy-related gene ATG7 exhibit impaired synaptic degradation and an increased number of immature synapses, which are associated with neurodevelopmental disorders like ASD [203]. Conditional knockout of *Atg5* in microglia inhibited postnatal neurogenesis in the dentate gyrus of the hippocampus, but not in the subventricular zone of 5×FAD mice, although the link with synaptic pruning was not studied [204]. Other studies suggest that inhibition of autophagy impairs the phagocytic capacity of microglia. For example, Beclin-1-mediated phagocytic dysfunction was found to be associated with impaired recycling of phagocytic receptors [205].

TREM2 (triggering receptor expressed on myeloid cells 2) is an immunomodulatory transmembrane receptor primarily expressed on microglia. Deficiency in TREM2 leads to an energy crisis in microglia, causing mitochondrial damage and triggering autophagy through activation of AMPK and impairment of phosphoinositide 3-kinase/PI3K-AKT-MTOR signaling pathways [206]. The increase in autophagy is thought to be a compensatory mechanism to manage cellular stress and maintain homeostasis. Although the direct link between TREM2-mediated autophagy and synaptic activity is not fully elucidated, the regulation of microglial autophagy by TREM2 can influence synaptic pruning and maintenance. Indeed, TREM2 is essential for microglia-mediated synaptic refinement during the early stages of brain development. The absence of TREM2 results in impaired synapse elimination, which is accompanied by enhanced excitatory neurotransmission and reduced long-range functional connectivity. This impairment is linked to behavioral changes such as repetitive behavior and altered sociability in animal models, which are also associated with neurodevelopmental disorders in humans such as ASD [207].

Autophagy deficiency in microglia is closely linked to increased inflammation, which can exacerbate neurodegenerative processes. A very recent study has identified a novel microglial population in the aging mouse brain that exhibits cytoprotective properties. This microglial subpopulation is characterized by active autophagy and plays a crucial role in protecting against neuroinflammatory damage, such as that observed in multiple sclerosis models. The protective effect of these microglia is autophagy-dependent, as demonstrated by increased neural and glial cell death when the autophagy gene *Ulk1* is specifically deleted in microglia [208]. Autophagy impairment in microglia is associated with the activation of inflammasomes, such as the NLRP3 inflammasome, which further amplifies the inflammatory response. For instance, the loss of autophagy-related genes like *Atg7* in microglia leads to increased production of inflammatory mediators and exacerbates neuroinflammation in models of neurodegenerative diseases like PD and AD [209,210]. A number of studies have demonstrated a beneficial role of mitophagy in suppressing microglia-mediated neuroinflammation by reducing mitochondrial ROS generation and inhibiting the NLRP3 inflammasome and CGAS-STING1 pathway [210]. Recent studies have demonstrated beneficial effects of mitophagy-inducing drugs such as the postbiotic urolithin A on reducing amyloid-β and MAPT/tau pathologies, and reversing cognitive deficits [211,212]. In aged animals, treatment with urolithin A alleviates age-associated neurological decline, enhancing mitophagy, improving synaptic connectivity, and reducing neuroinflammation [213]. This study highlights the potential of targeting mitophagy to modulate inflammation and improve healthspan during aging, offering insights into the mechanisms underlying age-related neuroinflammatory diseases.

Astrocytes also play a significant role in synaptic regulation, and astrocyte autophagy is implicated in various neurodegenerative and neuroinflammatory conditions. Autophagy in astrocytes is crucial for maintaining cellular homeostasis and supporting neuronal function by clearing damaged proteins and organelles. Furthermore, astrocyte autophagy is implicated in the regulation of synaptic plasticity and neuroinflammation. In major depressive disorder, autophagy-related pathways are significantly inhibited in astrocytes, which may contribute to the pathology of the disorder [214]. Enhancing autophagy in astrocytes has been suggested as a potential therapeutic strategy for alleviating symptoms of major depressive disorder by promoting synaptic health and reducing inflammation [214]. By maintaining synaptic integrity and function, astrocyte autophagy supports cognitive processes and neural circuit stability, underscoring its importance in brain health and disease [215].

## Autophagy and mitophagy dysregulation in neurodegenerative and neurodevelopmental disorders

### Neurodegenerative diseases

Neurodegenerative diseases in the aging population impose a significant healthcare burden. PD, PD with dementia, Alzheimer disease (AD), and frontotemporal dementia (FTD) are all characterized by dendritic shrinkage, impairments in synaptic function, and disturbances in mitochondrial homeostasis [187,216,217]. As critical junctions for neuronal communication, synapses depend upon mitochondrial bioenergetics and tightly regulated protein interactions to facilitate neurotransmitter release and reception. Moreover, neuronal synapses are typically positioned at a distance from the cell bodies, where new proteins are synthesized and most lysosomes are located. As a result, synapses are particularly vulnerable to disturbances in protein homeostasis and require orchestrated degradation mechanisms to eliminate malfunctioning proteins and mitochondria.

The majority of neurodegenerative disorders are distinctive *proteinopathies* characterized by increased production and/or retention of aberrant or misfolded proteins in the form of characteristic aggregates. Most of these diseases, including PD, AD, Huntington disease (HD), and amyotrophic lateral sclerosis (ALS) demonstrate pathologies that implicate disruption of canonical autophagy processing [218]. During neurodegeneration, protein aggregates form and are not effectively cleared by the autophagy-lysosome system – further aggravating cellular pathology, especially among individuals with gene mutations predicted to affect canonical autophagy function. Indeed, an array of genes and their protein products play key roles in the orchestration of autophagy and are mutated in the context of neurodegenerative disorders (Figure 1), many of which have already been reviewed [219–221]. The following section provides updates and categorizes these mutations as those that primarily affect autophagy initiation, cargo targeting, autophagosome maturation, trafficking or lysosomal degradation (though many may fit into more than one category). Neurodevelopmental and neurodegenerative disease mutations that affect cargo targeting (Table 1) for selective *mitophagy*, as well as other mutations that indirectly impact autophagy or mitophagy processes are included.

Table 2 summarizes an extensive (yet not comprehensive) list of proteins that are mutated or show altered expression in various neurodegenerative diseases, and which appear to play primary or secondary roles in autophagy. Mutated genes that are implicated in hereditary neurological disorders are also shown in Figure 1 in relation to the major steps of autophagy that they may regulate or modulate. While synaptic loss is a frequent manifestation of neurodegenerative diseases, most of these studies do not directly address the potential impact of these mutations on autophagy in or near synaptic structures. The final subsection (Disease-associated mutations and synaptic autophagy) highlights several mechanistic studies that specifically implicate neurodegenerative disease-associated genes in the emerging area of synaptic autophagy.

**Table 2.** Genes implicated in neurological diseases and their effects on autophagy/mitophagy.

	Gene	Disease(s)	Effects
Autophagy Initiation	*HTT*	HD^*a*^	Disrupted ULK1 interaction, impairs initiation of autophagy [222]
	*ATG9*	PD	Impaired initiation and autophagosome maturation [223]
	*VPS15*	NDi	Defective PtdIns3K complex formation, blunts PtdIns3P generation [224]
	*EPM2A*	LD	Reduced PIK3C3 complex activation, reduced PtdIns3P levels, and impaired autophagy initiation [225,226]
Autophagosome Formation	*WDR45*	BPAN	Dysregulated autophagy (increased or decreased) depending on if mutation is LOF or GOF [227]
	*ATG5*	PD, CP	Aberrant ATG12–ATG5-ATG16L1 complex formation (*upreg*) or assembly (LOF), impaired autophagosome formation [228]
	*ATG7*	HD, PD	Decreased expression disrupts ATG12–ATG5-ATG16L1 complex formation, impaired autophagosome formation [229]
	*ATG12*	PD	Higher or lower expression enhances or disrupts ATG12–ATG5-ATG16L1 complex formation [230]
Cargo-targeting	*BNIP3*	ALS	Lower expression decreases substrate targeting and autophagosome maturation [231]
	*NHLRC1*	LD	Reduce or impaired ubiquitin ligase activity impairs cargo-targeting/substrate-labeling [225,232]
	*NBR1*	PD	Forms aggregates in Lewy bodies impairing its ability to serve as an autophagy receptor; reduced pexophagy and aggrephagy [233]
	*SQSTM1/p62*	ALS	Impaired substrate targeting and autophagosome formation [234–236]
	*TAX1BP1*		Implicated in proteinopathies, not mutated. Loss results in impaired autophagosome tethering via NBR1 [237,238]
	*RB1CC1/FIP200*	PD	Implicated in PD, not mutated, Loss would result in impaired and reduced autophagosome tethering via NBR1 [237,238]
	*TBK1*	ALS	Lost kinase activity toward SQSTM1/p62 and OPTN, impaired autophagosome nucleation and maturation [220,234,239]
Mitophagy	*PINK1*	PD	Decreased expression or reduced kinase activity blunts production of p-S65-Ub and reduces activation of PRKN, impairing depolarization-induced mitophagy signaling [38]; loss of PINK1 may increase mitophagy through other pathways [151,240]
	*PRKN*	PD	Impaired ability to interact with ubiquitin or decreased expression reduces p-S65-Ub chain building and conjugation to OMM proteins, impaired mitophagy signaling [38]
	*OPTN*	ALS	Impaired substrate targeting, incomplete autophagosome formation [241,242]
	*CALCOCO2/NDP52*	*MS*^*b*^, AD	Mutations affecting MAP1LC3 interaction impair mitophagy; gain of function variant improves mitophagy and clear MAPT/Tau aggregates in AD [243,244]
	*CRLS1*	NDe	Reduced cardiolipin synthesis impairs mitophagy [22,245,246]
	*PTPMT1*	NDe	Impaired cardiolipin precursor processing, reduces cardiolipin and impairs mitophagy [247–249]
	*FBXO7/PARK15*	PD	Reduced PRKN translocation upon mitochondrial stress, less MFN2 ubiquitination, impaired mitochondrial turnover [250–252]
	*DNM1L/DRP1*	NDe	Decreased or increased GTPase activity and/or oligomerization impairs and enhances mitophagy, respectively [253]
	*OPA1*	ADOA	Decreased mitochondrial fusion dysregulates mitophagy [254,255]
	*MFN2*	CMTD	Decreased mitochondrial fusion dysregulates mitophagy [254,255]
	*VPS13C*	PD, ALS, PaD	Impaired and dysregulated lipid transfer at ER-mitochondrial contact sites, increased mitophagy and lysosomal turnover [256–258]
	*VPS13D*	HKMD	Impaired lipid transfer and/or mitochondrial fission dynamics, non-clearance of dysfunctional mitochondria [259–261]
	*PARK7/DJ-1*	PD	Impaired OPTN recruitment to tether OMM to MAP1LC3-II, accumulation of dysfunctional mitochondria, halted mitophagy [262–264]
Autophagosome Maturation	*CHMP2B*	ALS	Abnormal autophagosome buildup and impaired degradation/processing [265–267]
	*RAB7*	CMTD	Lost interaction with cargo and motor protein impairs autophagosome transport to lysosome [268]
	*RAB39*	PD	Reduced HOPS complex recruitment and impaired autophagosome-lysosome fusion [269,270]
	*VAMP1*	MyS	Impaired SNARE complex function, reduced autophagosome turnover [271,272]
	*VAMP2*	NDe	Impaired SNARE complex function, reduced autophagosome turnover [273]
	*STX1B*	PD	Impaired SNARE complex function, reduced autophagosome turnover [274–276]
Autophagosome Transport	*SNX14*	AD, PD, DS	Impaired autophagosome-lysosome fusion and accumulation of autophagosomes [277–284]
	*DCTN1*	ALS, PS	Impaired trafficking to lysosome and limited degradation, accumulation of autophagosomes [285–287]
	*DYNC1H1*	CMT, SMA	Impaired motor function reducing transport of cargo to lysosome, impaired trafficking and degradation [288,289]
	*KIF1A*	KAND, FTD-PD	Impaired transport/cargo trafficking leading to buildup of autophagic vesicles [290–292]
	*KIF1B*	CMTD	Impaired transport/cargo trafficking leading to buildup of autophagic vesicles [290,293]
	*KIF5A*	ALS	Impaired transport/cargo trafficking leading to buildup of autophagic vesicles [290,294]
	*KIF21B*	NDe	Impaired transport/cargo trafficking leading to buildup of autophagic vesicles [290,295]
Lysosomal Degradation	*EPG5*	VS, PD	Impaired autophagic flux and accumulation of autophagosomes [296,297]
	*VPS11*	GL	Impaired HOPS complex formation and reduced STX17 interaction impairing autophagic flux [298–301]
	*VPS41*	PD	Impaired HOPS complex formation and reduced STX17 interaction impairing autophagic flux [300–302]
	*ATP13A2*	PD	Increased lysosomal pH impairing hydrolase activity and lysosomal degradation [303], impairment of mitochondrial quality control [304]
	*GRN*	NCL, FTD-ALS	Increased lysosomal pH leading to accumulation of dysfunction lysosomes and impaired degradation [305–307]
	*GBA*	GD, PD	Loss of lysosomal hydrolase activity and impaired autophagic flux [308]
Protein Aggregation and Autophagy Impairment	*MAPT*	ALS, PSP, PiD	Disrupted microtubule stabilization and dysregulated ESCRT-III impairing autophagosome maturation and degradation [309,310]
	*C9orf72*	ALS	Reduced activation of Rab GTPases and impaired initiation [311]
	*SNCA*	DLB, PD	Blocked LAMP2A chaperone mediated autophagy, impaired lysosomal degradation/autophagosome-lysosome fusion [312,313]
	*PSEN1*	AD	Impaired cleavage of amyloid results in formation of insoluble aggregates, reduces MAPK/ERK-CREB activation causes reduces expression of TFEB and leads to lower expression of TFEB regulated lysosomal genes, impairing degradation [314]. Poor glycosylation of V-ATPase ATP6V0A1 subunits leads to their degradation, impairing lysosome acidification [315]
	*TMEM106B*	ALS	Lost stabilization of V-ATPase, disrupting lysosome acidification and impairing lysosome-mediated degradation [316]
	*TARDBP/TDP-43*	ALS	Dysregulation of TFEB (aberrant activation) and related genes, downregulation of DCTN, impaired autophagosome-lysosome fusion [317]
Retromer-linked	*VPS35*	PD	Impaired WASH complex recruitment impairing autophagosome formation; impaired lysosomal degradation [318,319]
	*APOE*	AD, PD	APOE4 variant dysregulated transport/processing of amyloid impaired during retrograde transport, formation of aggregates [320]
	*SORL1*	AD, PD	Downregulation or mutations reducing endosomal recycling of APP (and other cargo) leads to enhanced amyloid pathology [321–323]
Indirect Effects on Autophagy and Mitophagy	*LRRK2*	PD	Increased (aberrant) kinase activity alters autophagy initiation and impairs maturation, suppresses CCCP-induced mitophagy and increases postsynaptic autophagy/mitophagy [170,171,324–327]
	*UBQLN2*	ALS	Reduced MTORC activation and lysosomal acidification, impaired autophagy and mitophagy [234,328]
	*PEX13*	ZSS	Impaired protein transport import during peroxisomal biogenesis, leads to upregulation of peroxophagy [329–332] which impairs selective autophagy
	*VCP*	FTD, ALS, PDB	Impaired PtdIns3K complex assembly reduces PtdIns3P production, accumulation of autophagosomes [179,333–335]

^*a*^Abbreviations: AD, Alzheimer disease; ALS, amyotrophic lateral sclerosis; BPAN, β-propeller protein associated neurodegeneration; CP, cerebral palsy; CMT; Charcot-Marie-Tooth disease; DS, Down syndrome; FTD, frontotemporal dementia; GD, Gaucher disease; GL, genetic leukoencephalopathy; HD, Huntington disease; HKMD, hyperkinetic movement disorder; KAND; KIF1-associated neurological disorder; LD; Lafora disease; MS^*b*^, multiple sclerosis; MyS, myasthenic syndrome; NCL, neuronal ceroid lipofuscinosis; NDe, neurodevelopmental defects; NDi, neurodevelopmental disease; ADOA, autosomal dominant optic atrophy; PD, Parkinson disease; PDB, Paget disease of the bone; PS, Perry syndrome; SMA; spinal muscular atrophy; VS, Vici syndrome; ZSS, Zellweger syndrome spectrum disorders.

^*b*^MS is not a true neurodegenerative disease; instead, the pathology is driven by chronic inflammation that over time can result in neurodegeneration.

#### Mutations affecting autophagy initiation

*HTT*, the gene implicated in HD, pathogenically undergoes a polyQ/glutamate expansion in exon 1, resulting in a CAG trinucleotide repeat expansion that disrupts protein structure and canonical function [336]. Wild-type HTT (huntingtin) binds the C terminus of ULK1, an autophagy initiating kinase, and competes with MTORC1 to promote autophagy [337,338]. The polyQ/glutamate tract expansion present in patients with HD disrupts ULK1 interactions leading to impaired autophagic function [222]. Disruption of canonical HTT function due to polyQ expansion has also been reported to impair selective mitophagy [222]. Despite its well-known role in aggregation, variation in polyQ length may confer neuronal protection via changes in transcriptional programming; these authors propose that changes in polyQ expansion size potentially confer neuronal and evolutionary fitness [339].

ATG9 is known to localize to sites adjacent to phagophore formation in the early stages of autophagy and is believed to promote expansion of the autophagosome among other roles [196,340,341]. ATG9A plays a critical role in the mobilization of lipid droplets to the autophagosome and mitochondria, a *feed forward* signal in autophagy processing [197]. Upstream of its lipid scramblase activity, ATG9A is also a component of and plays a role in the formation and maturation of the ULK1 complex. Recent X-ray crystallography studies have defined the ternary structure of the ATG9S carboxyl-terminal tail bound to ATG13-ATG101 in the ULK1 complex [223]. Disruption of this interaction impairs downstream mitophagy activity [223]. The role(s) of ATG9 in the nervous system are emerging as discussed above, yet still understudied [198].

PIK3R4/VPS15 is a core component of the class III phosphatidylinositol 3-kinase (PtdIns3K) complex required for phosphatidylinositol-3-phosphate (PtdIns3P) generation in the early stages of autophagosome formation [220]. Loss of function mutations associated with neurodevelopmental disorders lead to defects in the assembly of the PtdIns3K complex and blunt subsequent generation of PtdIns3P, inhibiting autophagy and leading to the accumulation of autophagic substrates in mice and humans. In humans, mutant PIK3R4/VPS15 is associated with epilepsy and atrophy in the cortical region of the brain [224]. When ULK1-mediated phosphorylation of PIK3R4/VPS15, a pro-autophagy signal, is disrupted by targeted mutagenesis experimentally, unphosphorylated VPS15 leads to inhibition of autophagy [342]. However, whether disease-linked mutations in VPS15 impair autophagy signaling due to non-canonical mechanisms or due to a lack of phosphorylation by ULK1 has not been shown in the context of disease pathogenesis.

EPM2A/Laforin and NHLRC1/malin/EPM2B form a functional ubiquitin ligase complex that polyubiquitinates key components of the autophagic class III phosphoinositide 3-kinase complex (e.g., BECN1, PIK3C3/VPS34) to enhance PI3KC3 activity and facilitate autophagy initiation [225]. Mutations in the *EPM2A* gene, a glucan phosphatase, are linked to Lafora disease and disrupt autophagy initiation. Loss of functional EPM2A/laforin reduces PtdIns3P levels and results in defective PI3KC3 complex stabilization and impaired initiation of autophagy [225]. EPM2A/Laforin deficiency has also been implicated in autophagy dysfunction through an MTOR-dependent pathway, exacerbating the accumulation of autophagic substrates and cellular stress [226].

#### Mutations affecting autophagosome formation

WIPI (WD repeat domain, phosphoinositide interacting) proteins play an essential role in the early stages of autophagosome maturation. Mutations in WDR45/WIPI4 are associated with β-propeller protein associated neurodegeneration (BPAN) [343]. WDR45 plays a role in neural development and is expressed in excitatory synapses of mouse brains. Acute knockdown of WDR45 causes dendritic and synaptic abnormalities during corticogenesis which is rescued when the gene is re-introduced [343]. Loss of WDR45 impairs autophagy and BPAN-associated mutations alter autophagy activity, which is either increased or decreased dependent on the specific mutant expressed [227]. In the context of neurodegeneration, it was recently shown in cell culture and zebrafish models that loss of WDR45 induces ferroptosis independent of autophagy. Without WDR45, ATG2A localizes to ER-mitochondrial contact sites to enhance mitochondrial import of phosphatidylserine, a lipid prone to peroxidation, allowing for ferroptotic cell death [344].

Several mutations in the ATG class of proteins alter transcriptional signaling or protein function and affect autophagosome formation dynamics. In one study, a heterozygous variant of ATG5 in a single female PD patient was identified and demonstrated to enhance the transcriptional activity of the ATG5 promoter [228]. The enhanced transcriptional activity may act to alter autophagic signaling by enhancing aberrant (in this context) formation of the ATG12–ATG5-ATG16L1 complex. Variants of ATG7 in neurodegenerative diseases reduce autophagic activity [229]. Childhood ataxia or cerebral palsy-associated mutations in ATG5 ablate conjugation to ATG12 and/or protein expression, impairing autophagosome formation and autophagic flux [345,346]. Polymorphisms in the *ATG7* promoter region reduce transcription and are associated with earlier onset of HD [347,348] and sporadic PD [349]. These variants elicit reduced formation of the ATG12–ATG5-ATG16L1 complex, which is canonically accomplished by ATG7 activity [229]. PD-associated mutations upstream of exon 1 modulate *ATG12* transcription, likely due to structural genomic differences that alter promoter interactions with different transcription factors [230]. Although inferences could be made regarding how these mutations might elicit transcriptional changes to alter autophagy, additional *in vitro* and *in vivo* evidence are needed to clarify the roles that these mutations may play in regulating autophagy in the context of disease pathogenesis.

#### Mutations affecting cargo-targeting and mitophagy

##### Cargo-targeting for autophagy

Substrates and sub-cellular structures slated for selective autophagic degradation require autophagy receptors to facilitate their enclosure within the autophagosome membrane. In ubiquitin-dependent pathways, substrates for autophagy are labeled with ubiquitin, and autophagy receptors interact with ubiquitin on substrates to link them to the MAP1LC3-II-bearing autophagosome membrane as it forms [350,351]. Ubiquitin-independent autophagy receptors directly interact with substrates or compartment membranes to facilitate phagophore enclosure, such as tethering the outer mitochondrial membrane to MAP1LC3-II in the case of cardiolipin or BNIP3L. While there are no known mutations in *BNIP3L* linked to neurodegenerative diseases, it is upregulated in the cerebrospinal fluid of patients with ALS [231].

NHLRC1/malin is an ubiquitin ligase that forms a complex with EPM2A to initiate autophagy via ubiquitination of the PI3KC3 complex. *NHLRC1* is mutated in the context of Lafora disease. It also serves a functional role in labeling cargo with ubiquitin to facilitate ubiquitin-dependent autophagy, targeting various substrates that include BECN1, PIK3R4/VPS15, PIK3C3/VPS34, ATG14 and UVRAG [225,232]. Interaction of SQSTM1 with the complex enhances recognition and interaction with MAP1LC3-II [232]. Loss of function mutations in *NHLRC1* should thus impair cargo-targeting for autophagy in Lafora disease, but direct evidence of this has not been established.

NBR1 and SQSTM1 are autophagy receptors that facilitate the removal of ubiquitinated cargo [352]. SQSTM1 forms oligomers that link ubiquitin on autophagy substrates to MAP1LC3-II, and NBR1 terminates SQSTM1 polymerization by also binding ubiquitin chains on cargo. TAX1BP1 and RB1CC1/FIP200 interact with NBR1 to further facilitate autophagosome cargo binding [237,238]. Although SQSTM1 and NBR1 can facilitate mitophagy with the help of other adaptor proteins [38], they are more known for their roles in aggrephagy and pexophagy [353,354]. Loss of SQSTM1 disrupts normal NBR1 and RB1CC1/FIP200 function upstream; loss of TAX1BP1 disrupts downstream NBR1 function – in either case leading to impaired clearance of protein aggregates. *SQSTM1* is mutated in ALS and loss of SQSTM1 function (by mutation or genetic KO) increases aggregative proteinopathies in various models of neurodegenerative disease [355], similar to loss of TAX1BP1 [356,357]. Although NBR1 is not mutated in disease, it is found in glial cell protein inclusion bodies in PD [358]. TBK1 (TANK binding kinase 1) enhances autophagosome formation by interaction with and phosphorylation of OPTN or SQSTM1. ALS linked mutations in *TBK1* lead to loss of kinase activity, impairing autophagosome nucleation and maturation [220,234,239].

Although most studies of mutant HTT with polyQ expansion focus on its role in autophagy initiation, it may also play a role in cargo loading in the forming autophagosome. One study of human HD patient brain tissues and cells transfected with mutant polyQ expansions found that autophagosomes form and are turned over at a similar rate to wild-type cells; however, autophagosomes failed to properly sequester autophagic substrates, which persist in the cytosol [359]. This work suggests that more than one mechanism could contribute to autophagy impairment in HD.

##### Mitochondrial cargo targeting for mitophagy

Mitochondrial autophagy, or *mitophagy*, is the process by which damaged mitochondria are selectively turned over by lysosomal degradation. Mitophagy is accomplished broadly by mechanisms that are either independent or dependent on ubiquitin signaling [38,47] (Table 1). In the most studied pathway, the ubiquitin kinase and ligase pair, PINK1 and PRKN, work together to build chains of ubiquitin conjugated to OMM protein substrates – unique to these chains is that ubiquitin subunits are phosphorylated at serine 65 residues (p-S65-Ub), a post-translational modification (PTM) resulting from PINK1 kinase activity [360]. Mutations in the different key functional domains of PINK1 and PRKN have the potential to affect this process at various stages.

Systematic evaluation of PD-linked coding variants in *PINK1* and *PRKN* reveal disruption of normal protein expression, PINK1 autophosphorylation, PRKN translocation to mitochondria, PRKN phosphorylation by PINK1, the generation of p-S65-Ub, and mitophagic flux [361–364]. Other studies with PRKN variants describe disruption of its ability to interact with, handle, and transfer ubiquitin [365,366]. One PINK1 heterozygous mutation increases the risk of developing PD [367]. Some variants or PTMs in PINK1 or PRKN can also enhance mitophagy [365,368,369]. Studying the role of PINK1 and PRKN variants in the context of neurodegeneration *in vivo* has remained a challenge for the field, since germline knockout of either or both in mice does not result in neurodegenerative phenotypes without additional injury or insult, such as SNCA/α-synuclein overexpression [370].

Mitophagy receptors are bifunctional proteins that bind ubiquitin and MAP1LC3-II (*e.g*., OPTN) or OMM molecules capable of directly interacting with MAP1LC3-II (e.g., BNIP3, cardiolipin), acting to tether mitochondria to expanding autophagosomes. The ALS-associated mutations in OPTN not only affect cargo-targeting, but also impair downstream autophagosome and lysosomal fusion due to aberrantly formed autophagosomes [241,242]. Another autophagy receptor involved in mitophagy, CALCOCO2/NDP52, is implicated in human disease [243]. CALCOCO2/NDP52 is mutated in multiple sclerosis, a neuroinflammatory disorder, where impaired mitophagy is observed [244]. Interestingly, a more active variant of NDP52 was identified as a protective factor in AD, possibly due to its ability to clear pathological tau accumulation [371].

CRLS1 (cardiolipin synthase 1) plays critical roles in maintaining mitochondrial function, health and mitophagy signaling by synthesizing cardiolipin, a phospholipid that stabilizes respiratory complexes and signals for removal of dysfunctional mitochondria via mitophagy [22,254]. Mutations in CRLS1 are associated with developmental encephalopathy, optic atrophy, and hearing loss [245]. Autophagic disruptions among mutant carriers result from reduced cardiolipin production, impairing mitochondrial turnover [245,246]. PTPMT1 is a protein tyrosine phosphatase that facilitates cardiolipin biosynthesis via dephosphorylation of phosphatidylglycerophosphate, a precursor in the synthesis of cardiolipin [247,248]. Mutations in *PTPMT1*, similar to *CRLS1*, are linked to neurodevelopmental syndromes where patients present with cerebellar ataxia, optic atrophy, epilepsy, and bulbar dysfunction; specifically, bi-allelic variants have impaired production of cardiolipin [249].

FBXO7/PARK15 belongs to the F-box family of proteins that serve as substrate recognition subunits in multi-subunit E3 enzyme complexes. Mutations in FBXO7 result in severe autosomal recessive juvenile-onset PD that present with protein aggregation and reduced mitophagy [250]. FBXO7 has been studied for its roles in PINK1-PRKN mediated mitophagy where it may support PRKN translocation and MFN (mitofusin) ubiquitination [251], but recent evidence demonstrates a role in proteasomal degradation [252]. Specifically, FBXO7 expression is inversely correlated with PINK1 expression, and a recent study shows its involvement in proteasomal PINK1 degradation [174]. While there are clear pathogenic links to PD, FBXO7 is dispensable for chemically induced mitophagy (antimycin + oligomycin treatment) [372]. These contradictory findings suggest that understanding both physiological and pathological roles may depend upon identification of specific substrates targeted by FBXO7 under different contexts.

Mitochondrial transport and fission-fusion dynamics allow for the sorting and separation of damaged and healthy mitochondrial components. DNM1L/DRP1 is a GTPase that mediates mitochondrial fission, separating damaged mitochondrial components from those that are salvageable, followed by autophagosome membrane enclosure around the damaged fission product [38]. Mutations in DNM1L are not linked to a particular neurodegenerative disorder; rather, patients with mutations in DNM1L display heterogeneous phenotypes of neurological defects. Mutations in DNM1L can enhance or impair downstream mitophagy processing, depending on whether the mutation results in increased or decreased function [253]. OPA1 and MFN2 are proteins that regulate mitochondrial fusion of the inner or outer-mitochondrial membranes, and are associated with optic atrophy and Charcot-Marie Tooth disease, respectively [255]. Upon initiation of mitophagy, MFN2 is ubiquitinated by PRKN, leading to proteasomal degradation, and BNIP3 inhibits OPA1 [254]; this suggests that loss-of-function (LOF) mutations in OPA1 could dysregulate mitophagy.

VPS13D belongs to the family of VPS proteins which are required for the transfer of lipids across adjacent subcellular membranes [220,373]. VPS13D also plays an important role in mitophagy by the regulation of mitochondrial fission and ubiquitin mitochondrial localization [259,374]. One *Drosophila* study observed similar autophagy and mitophagy defects in PINK1- and Vps13D-deficient flies, but not in *park* (parkin)-deficient flies [259]. Two studies identified loss of function mutations in families with movement disorders wherein patient tissues exhibit defects in energy production and abnormal mitochondrial morphology [260,261]. Affected patients demonstrated developmental, cognitive, and motor function impairment which progress to ataxia, dystonia, and eventually loss of ambulatory capabilities [375]. Similar to VPS13D, VPS13C functions as a lipid transporter at ER-mitochondrial contact sites and loss of function mutations cause early-onset PD [256]. When VPS13C function is lost, mitochondrial function is perturbed, and cells accumulate lysosomes and mitophagosomes [257,258].

PARK7/DJ-1 is a multifunctional protein first identified among patients with PD where loss-of-function mutations cause disease [262]. The canonical roles of PARK7 include antioxidant activity, but it has also been implicated in regulating mitophagy downstream of PINK1 and PRKN; specifically, PARK7/DJ-1 translocates to depolarized mitochondria to facilitate OPTN recruitment [262,263]. Mutations in PARK7/DJ-1 impair mitochondrial turnover by disrupting OMM-protein-to-autophagosome-tethering, leading to accumulation of dysfunctional mitochondria and neuroinflammation [263,264]. Loss of PARK7/DJ-1 in rodent brains elicit accumulation of PTEN phosphatase which antagonizes AKT, with effects on mitophagy [376].

#### Autophagosome maturation and transport

##### Maturation

The endosomal sorting complex required for transport-III (ESCRT-III) complex is a key component of the broader ESCRT machinery that orchestrates autophagosome membrane remodeling during autophagosome maturation. The ESCRT-III complex facilitates both the closure of the autophagosome membrane and its subsequent fusion with lysosomes to ensure proper cargo degradation [265,377]. CHMP2B, a component of the ESCRT-III, plays important roles in maintaining normal autophagy via autophagosome membrane enclosure of substrates and lysosomal fusion. Mutations in CHMP2B, have been linked to neurodegenerative conditions such as ALS and FTD [266,267]. These mutations lead to defective autophagosome maturation and buildup of autophagic vesicles. As a consequence, toxic protein aggregates accumulate in neurons, including those containing ubiquitinated proteins and SQSTM1/p62 [378]. Additionally, CHMP2B mutations are associated with aberrant lysosomal morphology, altered regulation of transmembrane receptors, and dendritic retraction, all of which further contribute to neuronal dysfunction and cell death [265–267].

The Ras-associated binding (RAB) GTPases comprise a family of proteins that play an important role in the regulation and transport of intracellular-membranous cargo. In neurons, RAB regulation of vesicular trafficking includes neurotransmitter release, axonal anterograde and retrograde transport, dendritic branching and other morphological features, and neuronal migration during development [379]. Membrane trafficking defects are a common feature in neurodegeneration, cementing the importance of RABs [379,380]. RAB39 and RAB7 regulate autophagosome maturation by facilitating autophagosome-lysosome fusion. Their mutations are linked to PD and Charcot-Marie Tooth disease, respectively [379,380]. RAB39 plays a role in the recruitment of the HOPS complex (*discussed below*) which is critical in the late stages of autophagy [269,270]. RAB7 facilitates autophagosome transport by interaction with motor-protein adaptors for anterograde and retrograde transport [268]. Several other RAB proteins are implicated in neurodegenerative disorders where their expression or activity is modulated by up or downstream effectors [379,380].

SNARE (soluble NSF attachment protein receptor) proteins/complexes facilitate the tethering and fusion of the autophagosome membrane to the lysosome membrane [381]. The physiological roles of VAMP1 (vesicle associated membrane protein 1) and VAMP2 center on their participation in the SNARE complex, a key mediator of synaptic vesicle fusion with the presynaptic membrane [273,382]. Through interaction with STX1A (syntaxin 1A) and SNAP25, VAMPs play an integral part in facilitating neurotransmitter release by ensuring precise vesicle fusion events [273,382]. Beyond their canonical function in synaptic transmission, VAMP1 and VAMP2 may contribute to autophagy by affecting SNARE function and membrane fusion. Limited evidence suggests that VAMP1 may regulate autophagy induction via interaction with BECN1 [383,384]. Clinical correlations underscore the importance of these proteins in neuromuscular and neurological health. Mutations in VAMP1 have been linked to autosomal recessive congenital myasthenic syndrome and autosomal dominant spastic ataxia 1, both characterized by compromised neuromuscular junction transmission causing hypotonia, developmental delay, and spasticity [271,272]. Mutations in VAMP2 are associated with a distinct neurodevelopmental disorder marked by axial hypotonia, intellectual disability, and autistic features, with more severe cases manifesting central visual impairment, hyperkinetic movement disorders, and epilepsy [273]. STX1B is another SNARE protein. Mutations in STX1B are primarily associated with epilepsy and are associated with impaired vesicular trafficking, though the direct effects on autophagy have not been studied [274,275].

##### Transport

Sorting nexins (SNX) are a family of proteins that play major roles in the transport of intracellular constituents as well as their fusion to the lysosome during autophagy. A shared feature of proteins in this family is the Px domain, which allows for SNX interaction with various membrane phospholipids [385,386]. SNX14 is a sorting nexin that facilitates autophagosome-lysosomal fusion during autophagy [277] via a Px domain that interacts with PtdIns(3,5)*P*_2_ on the membrane of lysosomes [387]. Some evidence links members of the SNX family to AD, PD, and Down syndrome [277]; however, mutations in SNX14 are more strongly linked to cerebellar ataxia [388], cerebellar atrophy [278], and spinocerebellar ataxia 20 [279–283]. Disease-linked mutations result in lysosomal-autophagosome dysfunction due to impaired membrane fusion causing accumulation of autophagosomes [277,278,284].

DCTN1 is a subunit of the dynactin complex that functions as an adaptor to link vesicular or autophagosome cargo to the dynein motor complex. Transport of autophagosomes or vesicles to the lysosome is essential for autophagic degradation, and loss of function mutations are linked to Perry Syndrome and ALS [285]. Impaired lysosomal degradation and accumulation of autophagosomes occurs when DCTN1 function is impaired [286], likely also leading to mitochondrial dysfunction due to dysregulated transport and turnover. Protein aggregates of mutant DCTN1 can also accumulate, further limiting its function [287]. *DYNC1H1* encodes the dynein heavy chain of the dynein motor protein which facilitates retrograde transport of cargos; mutations are associated with spinal muscular atrophy and Charcot-Marie-Tooth disease with variable clinical features depending on the mutation [288], and which lead to impaired trafficking of mitochondrial cargo, and impaired autophagic degradation [289].

The kinesin family of motor proteins function as molecular motors that facilitate the transport of autophagic vesicles, among other intracellular cargo [290]. Mutations in KIF1A (resulting in KIF1-associated neurological disorder/KAND) [291], KIF1B (resulting in Charcot-Marie-Tooth disease) [293], KIF5A (resulting in ALS) [294], and KIF21B (causing neurodevelopmental disorders) [295] result in various neurological disorders where intracellular transport defects are evident. A double mutation in KIF1A elicits brain deposition of MAPT/tau and TARDBP/TDP-43 (transactive response DNA-binding protein 43), accompanied by spasticity and parkinsonism [292]. Loss of KIF1A, KIF1B, or KIF5A results in impaired autophagy and the accumulation of autophagic vesicles [290]. Though mutations or loss of KIF21B have not been directly studied in the context of autophagy, known deficits in intracellular trafficking would likely impair normal autophagic processes [295].

### Mutations affecting lysosomal degradation

EPG5 (ectopic P-granules 5 autophagy tethering factor) is implicated in autophagosome maturation and lysosomal degradation. EPG5 localizes to late endosomes and interacts with GABARAP proteins (a branch of the mammalian Atg8 family) and RAB7 to facilitate formation of the STX17-SNAP29-VAMP7/VAMP8 trans-SNARE complex, which allows for lysosome-autophagosome fusion [296,389]. The disease-linked EPG5 mutation (G336R) was not shown to alter its interaction with GABARAPs, but affected an EPG5-MAP1LC3-II interaction required for recruitment to the mitochondria during PINK1-PRKN mitophagy [389]. Mutations in EPG5 are primarily associated with Vici syndrome, a multi-organ system disorder with phenotypes including cardiomyopathy, hypopigmentation, cataracts, immunodeficiency, and callosal agenesis (i.e. malformation of the corpus callosum) [297,390]. Mutations in EPG5 among patients with Vici Syndrome lead to impaired autophagic flux and accumulation of autophagosomes due to impaired lysosomal-autophagosome fusion [296,297]. Various single nucleotide polymorphisms in EPG5 have also been linked to a higher risk of PD and earlier onset of pathology [391].

Vacuolar protein sorting (VPS) proteins are essential to the formation of the homotypic fusion and vacuole protein sorting (HOPS) complex (composed of VPS11, VPS16, VPS18, VPS33A, VPS39 and VPS41), which primarily functions to facilitate the fusion of autophagosomes with lysosomes in the late stages of autophagy via interaction with STX17 (syntaxin 17) [392,393]. Mutations in VPS11 result in autosomal recessive leukoencephalopathy, or white matter disease, and are associated with hypomyelination and impaired autophagic flux [298,299]. Loss of Vps11 in zebrafish results in phenotypic similarities to human genetic leukoencephalopathy with visual and sensorimotor defects [394]. Homozygous mutations in VPS41 have also been linked to neurodevelopmental dysregulation (i.e. cognitive impairment, ataxia, nystagmus, and cerebellar atrophy) [395]. VPS41 function has been linked to PD-related pathology in experimental models of disease [302]. While dysregulation of the HOPS complex (due to mutations in VPS proteins) may contribute to impaired lysosomal activity during neurodegenerative diseases, genetic links to leukoencephalopathies may be more strongly or specifically associated with genes other than *VPS11* [300,301].

Crucial to lysosomal activity is the function of lysosomal hydrolases that depend on the maintenance of an acidic, low pH environment. The activity of hydrolases, like GBA/glucocerebrosidase, require the normal function of lysosomal ATPases such as ATP13A2, which pumps protons into the lysosome and potassium out in a process dependent on ATP hydrolysis [303]. Mutations in ATP13A2 are associated with an autosomal recessive early-onset parkinsonian disorder called Kufor-Rakeb syndrome [396,397], in which normal ATPase activity is disrupted, leading to lysosomal dysfunction and aberrant SNCA/α-synuclein accumulation [303]. The decreased autophagic flux caused by deficient ATP13A2 activity results in impaired mitochondrial quality control with accumulation of fragmented, ROS-producing mitochondria [304]. ATP13A2 also functions as a polyamine transporter, and when dysregulated via mutations may cause cytotoxic increases in intracellular polyamine content [398].

The *GRN* gene encodes subunits of multimeric progranulin (PGRN), which also plays roles in the regulation of lysosomes by maintaining pH and promoting lysosomal function. Mutations in GRN result in reduced lysosomal acidification in neurons and cause lysosomal storage abnormalities observed in the FTD-ALS spectrum and neuronal ceroid lipofuscinosis [305–307].

Mutations in the lysosomal hydrolase glucocerebrosidase (GBA) are associated with PD in the context of lysosomal dysfunction [399,400]. It is thought that disease-associated SNCA/α-synuclein aggregation further aggravates dysfunction induced by GBA mutation by further limiting its trafficking to the lysosome [308]. It was recently demonstrated that reduced GBA protein levels disrupt canonical lysosomal membrane structure and dynamics, further impairing lysosomal function [401].

### Mutations causing protein aggregation and impaired autophagy

Several genes with mutations in neurodegenerative diseases form insoluble protein aggregates commonly observed in histopathological evaluations of patient tissue, which are not resolved by normal autophagic processing. In addition to the stress imparted on the cellular recycling system as uncleared aggregates, their normal functions are also disrupted. Impaired autophagic clearance is thought to be a primary contributor to the accumulation of protein aggregates in neurodegeneration and protein aggregation may intensify autophagic dysfunction, setting up a feed-forward cycle. Aberrant processing of protein aggregates by endogenous proteases may also lead to the creation of potentially toxic cleavage products. Although this is an incomplete list, the commonly studied genes whose mutations result in the formation of protein product aggregates and/or disrupt autophagy include *MAPT*, *C9orf72*, *SNCA*, *PSEN1*, *TMEM106B*, and *TARDBP*.

The *MAPT* gene encodes the microtubule associated protein tau, which plays important roles in the assembly and stabilization of microtubes and regulation of axonal transport, among other cellular functions [402]. While tau aggregates, called tangles, are prominently associated with AD, mutations in MAPT are primarily linked to FTD (pathologically, frontotemporal lobar degeneration with MAPT/tau pathology, or FTLD-MAPT/tau), which includes progressive supranuclear palsy, corticobasal degeneration, and Pick disease [403]. MAPT mutations have been long associated with impaired transport along cellular microtubules, due to disrupted stabilization [309]. More recently, mutant MAPT has been shown to dysregulate ESCRT-III complex formation, impairing cellular transport in various processes including autophagy [310].

The C9ORF72 (chromosome 9 open reading frame 72) protein activates RAB GTPases (that facilitate various aspects of autophagosome maturation and membrane trafficking necessary for lysosomal degradation) as part of a complex composed of itself, SMCR8 and WDR41. This GTPase activating complex can be recruited to lysosomes under cationic amino acid starvation conditions, binding to inactive RAB GTPases to decrease MTORC1 activity and enhance autophagy [311,404]. *C9ORF72* is mutated in the context of the ALS-FTD spectrum, with aberrant repeat insertion causing expansion of the gene at repetitive microsatellite regions (G_4_C_2_). The resulting dipeptide repeat proteins accumulate within protein aggregates, disturbing the normal functions of C9ORF72. Although one recent study has shown *in vitro* that some dipeptide repeat proteins can block autophagy under starvation conditions, it remains unclear as to whether this is an inciting event in autophagy inhibition or part of a feedback cycle driven by other cellular disturbances [405]. MG132 studies suggest that autophagic turnover of insoluble dipeptide repeat proteins under conditions of proteasomal impairment may provide protection against their accumulation in patients [311].

The *SNCA* gene encodes the α-synuclein (SNCA) protein, which may play an important role in supporting autophagosome-lysosomal fusion among other unclear functions when not mutated, aggregated, or overexpressed [406]. SNCA is deposited in pathological aggregates called Lewy bodies that are observed in PD and dementia with Lewy bodies [312]. Mutations in SNCA may increase protein aggregation, although the *SNCA* gene is mutated in only a small subset of cases, with wild type SNCA depositing in the brains of patients with sporadic PD or familial PD due to gene multiplication [407,408]. Mutations in SNCA impair its ability to be degraded by autophagy, perhaps due to impaired autophagosome-lysosomal fusion [312,406]. Two common mutants (A53T and A30P) affect the activity of LAMP2A, impairing chaperone mediated autophagy [313].

PSEN1 (presenilin 1) regulates lysosomal activity by stabilizing the vacuolar-type H^+^-translocating ATPase (V-ATPase) ATP6V0A1 subunit via glycosylation, allowing for acidification of the lysosome; loss of PSEN1 reduces ATP6V0A1 glycosylation and results in V-ATPase degradation and impaired lysosomal acidification [315]. PSEN1 is mutated in the context of AD, where mutations disrupt cleavage/processing of APP (amyloid beta precursor protein), leading to toxic aggregation of amyloid beta [305]. Mutant PSEN1 may also result in reduced MAPK/ERK-CREB activation, leading to decreases in TFEB (transcription factor EB; discussed in further detail below) and its lysosomal target genes [314].

Lysosomal TMEM106B (transmembrane protein 106B) plays a role in maintaining lysosomal acidification and stabilizing the lysosomal V-ATPase, via interaction with ATP6AP1 and ATP6AP2 [409]; TMEM106B may also play other roles in lysosomal biogenesis via signaling that enhances TFEB nuclear localization [410]. Mutations in TMEM106B are associated with increased risk of ALS, particularly in cases where patients also carry mutations in GRN and C9ORF72 [410]. TMEM106B filaments were recently found in various brain regions (mostly the frontal lobe) among patients with amyloid deposits, AD, corticobasal degeneration, and ALS [316]. Loss of TMEM106B can reduce lysosomal acidification due to impaired lysosomal V-ATPase stabilization and/or decrease lysosomal biogenesis via reduced TFEB signaling, as knockdown of TMEM106B reduces TFEB nuclear localization [409,410]. Loss of TMEM106B activity and expression enhances TARDBP/TDP-43 burden and is proposed to be a key determinant in the development of TARDBP/TDP-43 proteinopathies, as seen in ALS and FTD [410].

TARDBP/TDP-43 has multiple roles in cell function via regulation of RNA processing. It has been shown to be important for the stabilization of *ATG7* mRNA [411], potentiating autophagy. Both wild-type and mutated TARDBP participate in the formation of aggregates in ALS and FTD, with mutations in *TARDBP* representing a rare cause of ALS/FTD. Interestingly, while loss of TARDBP enhances TFEB nuclear translocation and gene expression of lysosomal genes, it also leads to impaired autophagosome-lysosomal fusion due to concurrent downregulation of DCTN1 [317], contributing to autophagic stress [192,412].

### Retromer-linked genes

Retrograde endosomal sorting is important for autophagy as it mediates the delivery of degradative hydrolases to the endo-lysosomal system; disruption of this process can lead to unprocessed autophagy substrates due to generalized lysosomal dysfunction [413]. In addition to facilitating end-stage autophagy (i.e. lysosomal degradation), the retromer complex also plays an important role in autophagosome formation via ATG9 recycling (discussed above) [414,415].

Retromer dysfunction, and genes with canonical roles in endosomal function, are primarily associated with protein aggregation in AD and PD, but other neurodegenerative disorders are pathogenically linked less prevalently (e.g., Down syndrome, hereditary spastic paraplegia, and neuronal ceroid lipofuscinosis) [413,416]. Interestingly, pathologically-expanded HTT protein was shown by one group to disrupt the endosomal-recycling function of Rab11a, but specific evidence of retromer dysfunction in HD has not since been established [417,418].

The retromer complex is composed of VPS35, VPS29, and VPS26, which serve functional roles in cargo recognition to facilitate retrograde endosomal sorting. VPS35 is a core subunit of the retromer complex [419], known for its role in facilitating lysosomal enzyme delivery. The WASH complex, which mediates actin nucleation on endosomes, regulates retromer activity with indirect effects on ATG9 trafficking. Interestingly, the PD-linked D620N mutation in VPS35 associates poorly with the WASH complex to diminish proper trafficking of ATG9 [318], but is more established as a regulator of lysosomal degradation of autophagosomes [319]. Patients affected by the VPS35^D620N^ mutation develop PD that is indistinguishable from sporadic PD, and knock-in mice harboring the mutation develop tau pathologies [420,421]. One recent study reports retinal SNCA/α-synuclein pathology in *vps35* knockout mice [422]. Interestingly, the D620N mutation may also play a role in regulating LRRK2 activity (see below), further complicating how this mutation may affect autophagy processing [423].

APOE (apolipoprotein E) is an important lipid transporter that interacts with the retromer and facilitates trafficking and processing of APP [320,424]. Though not *mutated*, inheritance of the E4 allele (i.e. APOE4) is one of the major risk factors for AD. Compared to APOE3, APOE4 shows a gain of function interaction with CLEAR (coordinated lysosomal expression and regulation) DNA motifs, inhibiting the stress-induced upregulation of TFEB-regulated transcripts for *SQSTM1, MAP1LC3B*, and *LAMP2* [425]. The *APOE4* allele is particularly disruptive to autophagic processes and shows gene dose dependent enhancement of amyloid and tau pathologies [320]. Although APOE is primarily known for its role in the regulation and processing of amyloid products, with APOE4 linked to their aberrant accumulation, recent experimental evidence has also established roles of APOE/APOE4 in dysregulated processing and/or aggregative pathology of SNCA and MAPT products [426,427]. However, direct linkages among these pathologies to retromer dysfunction associated with the E4 allele have not yet been established.

*Endosomal receptors* selectively deliver endosome cargo for various cellular processes. SORL1 (sortilin related receptor 1) is an endosomal receptor involved in the recycling of various cargo including APP [321]. SORL1 transcript downregulation is associated with AD; a few rare variants have been shown to increase clinical amyloid pathology in AD [321] and features of neurocognitive decline associated with amyloid pathology in PD [322]. Conversely, some SORL1 variants do not seem to affect amyloid processing or related pathology [323].

### Indirect effects on autophagy and mitophagy

VCP/p97 (valosin containing protein) belongs to the AAA(+)-ATPase family of chaperone-like proteins. Among its many functions, it has been shown to regulate autophagosome initiation and maturation [428,429]. Reduced VCP expression or activity results in accumulation of abnormally large and acidified immature autophagic vesicles containing ubiquitin [430]. Mounting evidence suggests that VCP regulates autophagy in a BECN1-dependent manner by promotion of ATXN3 (ataxin 3) deubiquitinase activity toward BECN1, thus regulating the assembly of the PtdIns3K complex I and subsequent PtdIns3*P* production [431,432]. More recent work implicates UFMylation of VCP which reportedly stabilizes BECN1 by promoting ATXN3 deubiquitinase activity; pathogenic VCP variants are associated with reduced UFMylation [433]. Mutations in *VCP* cause ALS-FTD, inclusion body myopathy, and Paget disease of the bone, with cellular pathology showing accumulation of abnormal autophagosomes, as also observed experimentally *in vitro* [179,333–335]. Although VCP mutations were not linked to parkinsonism (due to lack of pathogenic variants) in a large cohort of patients [434], reduced VCP expression is observed in a small cohort of PD patients and in the N-methyl-4-phenyl-1,2,3,6-tetrahydropyridine/MPTP mouse model of PD [435]. Despite the negative findings in that study, several case reports link specific pathogenic VCP variants to parkinsonism [335,436]. Notably, VCP is involved in such a wide array of cellular functions ranging from ER-associated degradation and cell cycle to Golgi biology, membrane trafficking and dendritic branching/spinogenesis, that the potential causal role of autophagy disruption to disease pathogenesis would need to be experimentally addressed for each particular mutation.

LRRK2 is a large protein with both kinase and GTPase domains. Silencing LRRK2 demonstrates a role in rapamycin-induced autophagy in inflammatory cells, in which its membrane recruitment plays a key role [437]. In neuronal cells, expression of PD-associated mutants LRRK2^G2019S^ and LRRK2^R1441C^ causes increased autophagy and mitophagy in the dendritic compartment due to post-synaptic mitochondrial calcium disruption; inhibiting autophagy protects against the resultant dendritic atrophy [170,171,324,438]. In contrast, other studies suggest that LRRK2^R1441C^ decreases mitophagy stimulated by chemical mitochondrial uncouplers, as evidenced by decreased p-S65-Ub staining in the soma [325]. Compartment-specific differences may also result from effects on axonal autophagosome trafficking [326]. Despite its prevalence in mutations underlying PD, the role(s) of LRRK2 in canonical physiological signaling are not completely understood outside of certain protein domain requirements for intrinsic phosphotransferase/kinase function [439]. Important to its function as a kinase, LRRK2 activity is hypothesized to be regulated by its many phosphorylation sites [440]. Phosphorylation of four key serine residues of LRRK2 regulate autophagy under basal conditions; these authors suggest that LRRK2 mediated phosphorylation of RAB8A and RAB10 regulates canonical autophagic signaling [441]. Notable to the study and the finding’s relevance to human disease is that PD associated mutations in LRRK2 displayed decreased phosphorylation of sites implicated by the quadruple mutant, despite increased kinase activity overall.

UBQLN2 (ubiquilin 2) is an adaptor protein initially known for its function in cargo delivery for proteasomal degradation that has since also been recognized for its role mediating autophagic flux through interaction with MAP1LC3 [442]. UBQLN2 also modulates autophagic flux via positive regulation of MTORC1 and promotion of lysosomal acidification [443,444]. Another study reports that UBQLN2 regulates mitophagy via cooperation with HSPA/HSP70 [445]. Mutations in *UBQLN2* result in ALS with cellular features of abnormal protein aggregation [234], aberrant polyubiquitination of proteins [297], impaired autophagy [328], mitochondrial dysfunction [446], and TARDBP/TDP-43 aggregation [447]. Specifically, the P506T mutation was shown to drive autophagy-related dysfunction [448].

*PEX13* encodes a transmembrane protein that regulates protein import to peroxisomes during peroxisomal biogenesis [449,450]. Loss of function mutations in PEX13 are associated with Zellweger syndrome spectrum disorders, and models of disease also demonstrate impaired clearance of damaged mitochondria [329–332]. However, more recent studies demonstrated that loss of PEX13 upregulates pexophagy (autophagic degradation of peroxisomes); the authors suggest that the increase in pexophagy, rather than loss of PEX13 itself, impairs other forms of selective autophagy (i.e. mitophagy) [451,452].

TFEB is a central regulator for the expression of multiple genes related to autophagy-lysosomal function [453]. Among gene variants associated with PD, 18 lysosomal genes important for autophagic flux are also regulated by TFEB [454]. Although TFEB has been associatively linked to variety of neurodegenerative diseases, the current evidence is mainly correlative [455]. The nutrient sensing functions of lysosomes also regulate their own biogenesis by activation of TFEB via inactivation of MTORC (which normally suppresses TFEB) [456]. Other transcription factors can regulate the expression of various autophagy or mitophagy genes (e.g., *KANSL1* and *PINK1*) [457]. Understanding how changes in transcriptional regulation can affect autophagy in the context of neurodegenerative diseases implicates higher order chromatin remodeling changes, an evolving area of neurodegenerative disease research [458], and further work may reveal novel explanations for some cases of idiopathic neurodegeneration. Both transcriptional regulation, by transcription factors or co-activators, or changes in chromatin structure present areas for future study that may identify new targets for therapeutics.

### Disease-associated mutations and synaptic autophagy

The sections above summarize both established and some emergent genes whose mutations are associated with various neurodegenerative disorders, their normal roles in autophagy function, and how mutations disrupt this function. Although there is not necessarily a biochemical distinction between general cellular autophagy and synaptic/perisynaptic autophagy, it is clear that autophagy and mitophagy can be triggered in specific distal subcompartments of the neuron by localized synaptic stimuli [2,51,74,127,143,170]. Here, we will briefly discuss neurodegenerative genes and their mutations that have been specifically studied in the context of perisynaptic autophagy and mitophagy.

Studies have established the important role of the *Drosophila* endocytic adaptor EndoA (human homolog SH3GL2/endophilin A1) and the human kinase LRRK2 in presynaptic autophagosome formation. EndoA is activated upon LRRK2 mediated phosphorylation of its BAR domain at S75, promoting the formation of highly curved membranes and generation of autophagosomes; this was confirmed by authors using nonphosphorylatable mutant EndoA^S75A^ vs. its phospho-mimetic S75D, which promoted the generation of tubular and curved membranes, respectively, in response to starvation [57]. As mentioned above, another PD risk variant highlights the importance of the D265 residue in autophagosome formation in response to calcium flux [73]. Moreover, PD-linked G2019S and R1441C mutations indirectly promote postsynaptic mitophagy during elevated excitatory synaptic activity and mitochondrial calcium toxicity through effects on MCU (mitochondrial calcium uniporter) [170]. These studies highlight how EndoA-related autophagy activity can be modulated by LRRK2 activity, starvation, or calcium influx at the presynapse. A study using *sh3gl2* knockout mice further showed that loss of SH3GL2/endophilin A1 leads to impaired synaptic autophagy; both *in vitro* and *in vivo* models suggest that interaction of SH3GL2/endophilin A1 and FBXO32 is necessary for autophagosome formation [459]. Extrapolating from previous discussions of LRRK2 mutants, disease-linked variants in LRRK2 could aberrantly enhance or limit synaptic autophagy and mitophagy, depending on the specific mutant and how LRRK2 kinase activity is modulated with respect to SH3GL2/endophilin A1.

SYNJ1 (synaptojanin 1) is another endocytic adaptor that facilitates synaptic autophagosome maturation at the pre-synapse. The SACM1L/SAC1 phosphoinositide phosphatase seems crucial for Synj-mediated PtdIns3*P* and PtdIns4*P* dephosphorylation during the maturation of autophagosomes in fruit fly synapses. The PD linked mutation R258Q impairs SACM1L/SAC1 phosphatase activity and leads to the accumulation of WIPI1/ATG18A, blocking autophagosome maturation [76].

Other proteins implicated in synaptic autophagy include the heterotetramer adaptor protein complex 2/AP-2 complex, which functions in autophagosome retrograde transport to enhance autophagy, and BSN/Bassoon, which inhibits autophagy via an ATG5 interaction that limits ATG12–ATG5 complex formation; these proteins are mutated in the context of hereditary spastic paraplegia and PSP, respectively [460]. The motor protein KIF5A promotes lysosomal motility in the synapses of primary rat hippocampal neurons, and disease related mutations in ALS impair autophagic flux/cargo turnover [461]. As discussed in greater detail above, ATG9 plays important roles as a lipid scramblase in autophagosome formation and expansion as well as synaptic vesicle transport; loss of ATG9 results in impaired neuronal health and development [91,198].

Using information discussed in the preceding sections, one might be tempted to extrapolate how synaptic autophagy may be modulated during disease based on non-human model systems; however, while the core mechanisms of autophagy are generally conserved across cells, different local environments and cell types could have notable variations in regulatory mechanisms, specific pathway(s) utilized, thresholds for induction, and/or rates of autophagy completion. Most studies linking causal mutations to autophagy-related genes in neurodegenerative diseases rely upon large-scale genome-wide association studies to identify novel variants. At this point, studies that specifically focus on synaptic autophagy and mitophagy are scarce as most efforts have been focused on understanding molecular interactions by which these disease-linked gene mutations may affect autophagy and mitophagy in general. Much more work is needed to understand autophagy regulation by neurodegenerative disease related genes, and the effects of their mutations in the context of the synapse.

### Neurodevelopmental disorders

Neurodevelopmental disorders are a group of conditions that affect the development of the nervous system, often leading to cognitive, behavioral, and social impairments. Neurodevelopmental disorders primarily impact early brain development and manifest in childhood. Symptoms can persist into adulthood, although the severity and specific manifestations may change over time. Their causes can include genetic factors, environmental influences (e.g., prenatal exposure to toxins), and perinatal complications. Recent research has highlighted the critical role of autophagy, a cellular process that degrades and recycles damaged or unwanted components, in the pathogenesis of these disorders. Here we will summarize the recent evidence linking mutations in autophagy genes with neurodevelopmental disorders (Table 3).

**Table 3.** Main neurodevelopmental disorders associated with mutations in autophagy proteins.

Mutated gene	Disease	Symptoms	Role in autophagy pathway	Re ference
*Atg5*	Neurodevelopmental delay	Congenital ataxia, mental retardation	Core machinery	[346]
*Atg7*	Neurodevelopmental alterations	Brain, muscle, endocrine, facial dysmorphism	Core machinery	[462]
*Ambra1*	Neural tube defects		Core machinery	[463]
*PIK3R4/VPS15*	Neurodevelopmental	Cortical atrophy, intellectual impairment,	Core machinery	[224]
*WIPI2*	Neurodevelopmental delay	intellectual impairment, reduced brain volume	Core machinery	[464]
*WDR45/WIPI4*	BPAN	Seizures, developmental delay, dementia, dystonia	Core machinery	[465]
*WDR45B/WIPI3*	El-Hattab-Alkuraya syndrome	Intellectual disability, spastic quadriplegia, microcephaly, and early-onset refractory epilepsy	Core machinery	[466]
*SPG11*	Spastic paraplegia 11	Spasticity, pyramidal weakness, corpus callosum affection, epilepsy	Lysosome	[467]
*SPG15*	Spastic paraplegia 15	Spasticity, pyramidal weakness, corpus callosum affection, epilepsy	Autophagosome maturation	[467]
*EPG5*	Vici Syndrome	Corpus callosum agenesis, hypotonia, cataracts, epilepsy, microcephaly	Autophagosome-lysosome fusion	[390]
*SNX14*	Autosomal recessive cerebellar ataxia	Ataxia, cerebellum atrophy, mental retardation, seizures	Autophagosome-lysosome fusion	[388]

### Mutations involving the core autophagy machinery

A homozygous missense mutation in *ATG5* found in two siblings resulted in congenital ataxia, mental retardation and developmental delay [346]. The mutation results in decreased autophagy flux and impaired ATG12–ATG5 conjugation in patient cells. Yeast models with the equivalent mutation showed a 30–50% reduction in induced autophagy, while *Drosophila* expressing the mutant human *ATG5* exhibited severe movement disorders compared to those expressing wild-type *ATG5* [346]. Recent research has identified deleterious, recessive variants in the *ATG7* gene in individuals from five families exhibiting neurodevelopmental alterations that include brain, muscle, and endocrine involvement. Patients had abnormalities of the cerebellum and corpus callosum and various degrees of facial dysmorphism [462]. The identified mutations in *ATG7* lead to either a significant reduction or a complete absence of the ATG7 protein; this results in substantial reduction of autophagic sequestration and flux in patient-derived fibroblasts and skeletal muscle tissues compared to healthy controls [462]. Interestingly, despite the severe loss of ATG7 function, patients with these mutations have survived to adulthood, suggesting that humans may be more tolerant to the loss of ATG7, or that compensatory mechanisms exist that are not present in mice, wherein *atg7* knockout results in early postnatal death within 24 hours after birth [468]. These findings highlight the critical role of core autophagy genes in human neural development.

AMBRA1 (autophagy and beclin 1 regulator 1) positively regulates autophagy initiation by facilitating and activating the BECN1-PIK3C3/VPS34 complex that is required for autophagosome nucleation. In humans, a specific intronic SNP (rs3802890-AA) in the *AMBRA1* gene has been associated with autistic features, particularly in women. This SNP is located within a non-coding RNA, potentially affecting mRNA stability and expression [463]. Missense mutations in the *AMBRA1* gene have been found in patients with neural tube defects. Four of these mutations impair autophagy initiation, as demonstrated in cell lines and zebrafish models, indicating a loss-of-function effect compared to wild-type *AMBRA1* [469]. *Ambra1*-deficient mice exhibit early embryonic lethality, with neural tube defects including exencephalia and spina bifida [470]. Heterozygous *ambra1*-deficient mice (ambra1+/gt) display ASD-like symptoms, including social interaction and communication deficits, repetitive behaviors, and cognitive rigidity, with these features being more pronounced in females [471]. In summary, AMBRA1 plays a significant role in neurodevelopmental disorders, particularly ASD, with evidence supporting its involvement in both genetic predisposition and neurophysiological manifestations. Its role is also influenced by sex, with distinct effects observed in females.

PIK3R4/VPS15 plays a crucial role in neurodevelopmental disorders by impacting autophagy through its involvement in the PIK3R4-PIK3C3/VPS34-BECN1 complex, essential for autophagosome formation. Mutations in *PIK3R4*, such as the L1224R mutation identified in humans, are linked to cortical atrophy, intellectual impairment, and other neurological symptoms [224]. These mutations disrupt autophagy in dermal fibroblasts derived from affected individuals, leading to reduced stability of the PIK3C3-BECN1 complex, increased levels of SQSTM1/p62, and defective autophagic degradation. The resulting accumulation of autophagic substrates contributes to neuronal loss and various neurodevelopmental deficits, including cortical dysplasia and epilepsy [224]. In mice knockout of *Pik3r4* in neurons leads to a fractured pyramidal cell layer in the hippocampus, with an increased number of ectopic pyramidal cells as well as severe cortical atrophy [224].

Defects in WIPI2, which plays an important role in autophagosome formation via ATG16L1 interaction and PtdIns3P expansion, cause a neurodevelopmental disorder characterized by global developmental delay/intellectual disability, reduced brain volume, and variable other features [464]. The authors identified a novel homozygous mutation (c.G745A;pV249M) in the *WIPI2* gene in an extended family with a complex developmental disorder. Functional studies showed that the WIPI2^V249M^ mutation results in reduced WIPI2-positive membranes and decreased autophagosome formation and flux. The WIPI2^V249M^ mutation is located near the PtdIns3P binding site and is predicted to disrupt the interaction between WIPI2 and ATG16L1, which is required for autophagosome formation [464]. A more recent study identified two novel homozygous *WIPI2* variants that expand the molecular and phenotypic spectrum of WIPI2-related disorders, with a wide range of clinical presentations from severe, progressive neurodevelopmental impairment to a milder, non-progressive phenotype. Functional studies in cell lines showed that the *WIPI2* variants differentially impact autophagy, suggesting dysregulation of the early steps of the autophagy pathway [472]

Mutations in other WIPI proteins also cause neurodevelopmental disorders. Defects in *WDR45/WIPI4* cause BPAN, a disorder with a biphasic presentation of early childhood onset seizures and global developmental delay followed by progressive dementia, parkinsonism, and dystonia in adolescence or early adulthood [465]. Studies reveal that BPAN patient cells show autophagy defects, with improper autophagosome formation [465]. Mice with *wdr45* KO display axon swelling and cognitive impairments, mimicking BPAN symptoms [473]. El-Hattab-Alkuraya syndrome is a neurodevelopmental disorder characterized by global developmental delay, intellectual disability, spastic quadriplegia, microcephaly, and early-onset refractory epilepsy [474]. This syndrome is linked to mutations in the *WDR45B/WIPI3* gene, which is involved in the autophagy pathway [466]. Homozygous nonsense mutations in *WDR45B*, such as R225X and Q267X, lead to a loss of function, contributing to the neurological and developmental abnormalities observed in affected individuals [466]. The disorder follows an autosomal recessive inheritance pattern and is associated with significant brain abnormalities, including enlarged ventricles, cortical thinning, and reduced white matter volume [466].

### Mutations in other genes that impact autophagy pathways

Hereditary spastic paraplegia (HSP) is a group of neurological disorders characterized by upper motor neuron degeneration, resulting in symptoms like spasticity and pyramidal weakness, with complex forms including additional neurological and extraneurological features such as epilepsy and skeletal abnormalities [467]. Mutations in *SPG11/SPATACSIN* and *ZFYVE26/SPG15/SPASTIZIN* are linked to HSP with alterations in the corpus callosum and are responsible for about 70% of such cases; these proteins are involved in autophagosome maturation and lysosome reformation [467]. Patients with hereditary spastic paraplegia type 49 (SPG49), caused by mutations in *TECPR2* (tectonin beta-propeller repeat containing 2), present with a thin corpus callosum, cerebral and cerebellar atrophy, and moderate to severe intellectual disability, with TECPR2 playing a role in autophagy by maintaining endoplasmic exit sites for autophagosome formation [475]. Additionally, mutations in *AP5Z1/SPG48* and *AP4B1/SPG47*, disrupt lysosomal activity and autophagy, further implicating autophagy defects in the neurodegeneration observed in HSPs [476]. A recent study identified TECPR2-related hereditary sensory and autonomic neuropathy in two Palestinian siblings, presenting with acute encephalopathy and severe multi-organ dysfunction, expanding the clinical and genetic spectrum of *TECPR2* mutations [477]. These findings suggest that disruptions in the autophagic pathway play a significant role in corticospinal tract degeneration and the development of hereditary spastic paraplegias.

*EPG5* is the homolog of the *Caenorhabditis elegans* gene *epg-5*, a specific autophagy gene that promotes fusion of autophagosomes with late endosomes/lysosomes during autophagy [296]. Vici syndrome is a severe multisystem disorder caused by recessive mutations in the *EPG5* gene, which impairs autophagy by disrupting the fusion of autophagosomes with lysosomes [390]. A hallmark feature is agenesis of the corpus callosum, where the tissue connecting the brain’s hemispheres fails to develop properly. Patients typically have weak muscle tone (hypotonia) and cataracts, epilepsy, microcephagy and loss of learned skills [390]. Patient-derived fibroblast samples exhibit accumulation of SQSTM1 and NBR1, indicating impaired autophagosomal cargo clearance, and decreased colocalization of MAP1LC3B with LAMP1, suggesting dysfunctional autophagosome-lysosome fusion [478]. In *epg5*^*-/-*^ mice, similar autophagic disruptions are observed, including selective neuronal degeneration which partially mirror human Vici syndrome features such as agenesis of the corpus callosum [479]. Additionally, these mice show retinal degeneration, evidenced by decreased outer nuclear layer thickness and reduced electroretinogram responses, resembling retinitis pigmentosa [480]. These findings underscore the importance of EPG5 in maintaining autophagic function and its role in the pathogenesis of Vici syndrome, characterized by neurodevelopmental and neurodegenerative symptoms.

Mutations in the *SNX14* gene cause autosomal recessive spinocerebellar ataxia 20 (SCAR20), characterized by cerebellar atrophy, motor ataxia, and intellectual disability. Patient-derived fibroblasts with *SNX14* mutations exhibit large lysosomes and impaired autophagosome clearance, indicating lysosomal dysfunction [278,388]. Studies have identified *SNX14* mutations in several consanguineous families, demonstrating a loss of protein function and increased cytoplasmic vacuolation in cultured fibroblasts. These findings underscore the importance of SNX14 in cerebellar development and maintenance, with evidence suggesting its role in mediating lysosome-autophagosome fusion [278]. In zebrafish models, *snx14* knockdown results in loss of cerebellar parenchyma and decreased Purkinje cell numbers, mimicking the human condition [278]. Additionally, SNX14 deficiency in mice leads to defective axonal mitochondrial transport in Purkinje cells, contributing to cerebellar ataxia, which can be reversed by valproate treatment [481]. SNX14 is also crucial for neuronal excitability and synaptic transmission, as its deficiency reduces intrinsic neuronal excitability in mouse cortical neurons [482]. These studies highlight the critical role of SNX14 in maintaining neuronal function and structure, particularly in the cerebellum, and its involvement in the pathogenesis of SCAR20.

In conclusion, mutations in several core autophagy genes are linked to neurological disorders, often with neurodevelopmental phenotypes. For instance, mutations in ATG5 are linked to congenital ataxia and developmental delay, while WIPI2 mutations result in global developmental delay and intellectual disability, demonstrating the diverse roles of autophagy proteins in neuronal health. Moreover, disorders like BPAN and El-Hattab-Alkuraya syndrome further illustrate the potential impact of impaired autophagy on neurodevelopment, with symptoms ranging from intellectual impairment to epilepsy. Despite this, it is important to note that many individuals with mutations in autophagy-related genes can exhibit normal early development, suggesting that the role of autophagy in the nervous system may be highly context-dependent, manifesting after failure of compensatory mechanisms, or becoming more critical under conditions of stress or aging. More studies are needed to fully understand how specific mutations lead to varied phenotypes, as proteins involved in autophagy may have multiple, unknown roles or interact with different cellular pathways. Additionally, the precise mechanisms by which autophagy modulates synaptic balance and contributes to conditions like epilepsy and ASD require further investigation. Understanding these complexities is crucial for developing targeted therapies for neurodevelopmental disorders linked to autophagy dysfunction.

### Biomarker and treatment potential of autophagy-related pathways

Autophagy is a dynamic response to cell stress and nutrient availability. Clinical outcomes and disease effects will depend on the nature, extent and duration of these conditions and the expression/activities of the respective autophagy genes. The understanding of disease pathology and progression in neurodegenerative disorders has improved the last several decades, but current biomarkers available to support diagnostic efforts do not allow for the evaluation of autophagy activity. As previous and novel therapies target the autophagy system, it will be useful to understand to what degree autophagy is dysregulated and in what direction, so treatments can be titrated to individual patients. Modern treatment of neurodegenerative disorders target autophagy either by enhancing bulk autophagy or targeting some specified point in the pathway implicated in disease models. However, it is important to note that any efficacy attributed to autophagy modulation must be experimentally proven, as these therapies may also target other functions of these proteins. For example, while PINK1 and PRKN activators have been designed to enhance depolarization-induced mitophagy in PD, they may also target non-autophagy functions of these proteins [174,483]. Given that the impact of activating autophagy or mitophagy may be protective, detrimental or neutral, depending upon the ability to complete autophagy and replace degraded components [3,192], it is critical that multiple modalities for testing and treatment of neurodegenerative disorders be developed due the inherent heterogeneity of these diseases.

#### Biomarkers

While a myriad of previous work has characterized the many protein pathologies in neurodegenerative disorders that are either mechanistically linked to or correlated with autophagy dysfunction, contemporary studies seek to assess autophagy genes as biomarkers in neurodegeneration. Perhaps the greatest challenge in the evaluation of autophagic activity in humans is that autophagy is a *dynamic* process whereas measurements from patient samples only provide *static* “snapshots” of the autophagosome markers that may or may not correlate with activity or flux. Measurement of MAP1LC3-II (lipidated MAP1LC3-I) to housekeeping protein ratios are a classic method of determining if cells exhibit increased autophagosome levels, but such measurements *taken alone* do not inform about upstream processes affecting autophagy initiation or downstream completion of lysosomal degradation. The snapshot is generally informative regarding autophagic dysregulation, but current methods do not provide for the determination of autophagic flux (e.g., elevations in autophagosome content could result from robust autophagy initiation and/or from impaired intracellular trafficking and lysosomal dysregulation). Ideally, evaluation of autophagy would include clear indicators of autophagosome formation and degradation, dependent upon the autophagy machinery, and functional readouts relating autophagy parameters to cell health; unfortunately, this approach currently lacks feasibility, would be very expensive, and requires standardized definitions or benchmarks of autophagy processes in specific regions of the brain (e.g., basal and stressed flux ratios, kinetics of initiation under defined stimulation conditions, lysosome activity, etc.) in healthy individuals, with which to compare against patient samples. Even in more controlled, inbred animal model systems, the development of accurate and dynamic biomarkers of autophagy are needed to better understand the molecular pathology of autophagy dysregulation.

Given the importance of PTMs in governing the activity or functional status of many autophagy and mitophagy proteins, valuable information may be inferred from their study. Although still representing static readouts, understanding PTMs and multiplexed assessment of many autophagy genes would at least allow discrimination of activation states for such proteins. Ultimately, machine learning, artificial intelligence or other systems-based methods may be needed to develop an improved understanding of how flux states are disrupted in individuals and populations affected by neurodegenerative disease. Here, we highlight potential future prognostic biomarkers seeking to define disease severity and the impact on autophagy/mitophagy among patients.

In addition to the well-known lipidation of species required for intracellular membrane incorporation, MAP1LC3 and other Atg8-family proteins are also modified by phosphorylation and other PTMs that further regulate their interactions and functions in governing autophagy processes [484,485]. Similarly, the various autophagy receptors are complexly modified during autophagy [486]. Understanding the PTM code of protein groups (e.g., autophagy receptors) paired with technical advances to monitor PTM changes of protein involved in autophagy may allow for real-time evaluation of autophagy initiation, progression, and execution – given that basal function of such targets is well-defined. Also for consideration are the individual protein domains modified by PTMs. Both the type of PTMs and the unique sites they modify can exert distinctly different functional effects on the protein in question.

Biochemical and functional analyses in recent years revealed that SQSTM1 can be phosphorylated in its oligomerization, MAP1LC3-interacting, or ubiquitin binding domains, which consequently direct it to be more or less engaged in aggrephagy, mitophagy, or secretory autophagy [38]. The SCOC protein was not previously established as an autophagy receptor, but one study identified a LIR-related sequence, which conferred SCOC autophagy receptor/adaptor action following phosphorylation of serine residues adjacent to the LIR [487]. This indicates that potentially many more LIR-related proteins could behave as autophagy receptors following some PTM that enhances its affinity for MAP1LC3 or similar Atg8-family proteins. PTMs of MAP1LC3 (including ubiquitination, lipidation, acetylation, and phosphorylation) can throttle its affinity for autophagy receptors, resulting in various effects on autophagy [484,485]. In theory, this could mean that uncovering specific PTMs of MAP1LC3 combined with PTMs regulating autophagy receptors may reveal the snapshot of current efforts in the autophagy landscape (e.g., more aggrephagy, mitophagy, or generalized autophagy). Combined evaluation of phosphorylation states for multiple key proteins could reveal the prevailing autophagy signaling mechanism in a given sample.

Additionally, upstream analyses of kinase signaling/activity would provide useful information on whether the background cellular context is permissive or inhibitory for autophagy. TBK1 is a kinase that phosphorylates various autophagy receptors and MAP1LC3-II, providing a feed forward signal for autophagy. However, different sites of MAP1LC3 and other GABARAP phosphorylation can differentially regulate lipidation or other biochemical features which impact the progression of autophagy at different stages [488], providing yet another layer of complexity to be decoded with regard to site specific combinatorial effects. One study newly found that phosphorylated TBK1 accumulates in protein aggregates in an AAV-C9orf72(G_4_G_2_)_149_ mouse model [489]. Introduction of a reduced activity TBK1 mutant in this mouse model exacerbated phenotypes including an increase in TARDBP/TDP-43 pathology and accumulation of irregular endosomes [489]. Utilization of accessible biomaterials or alternative methods for *in vivo* evaluation of pTBK1 in the brain (e.g., PET-CT using radiolabeled antibodies) may be considered.

As another example, PINK1-mediated phosphorylation of RHOT/MIRO at different sites have opposite effects on RHOT/MIRO stability; combinatorial studies show that PINK1-mediated stabilization of RHOT/MIRO is the dominant effect [163]. One review also proposes monitoring the oxidation state of the antioxidant scavenging PARK7/DJ-1 in relation to cellular pathology with respect to ROS [264]. Working toward more thorough descriptions of various PTMs, the sites they modify, and how different types of PTMs work in concert on the same proteins, would further enhance the ability of an individual *snapshot* of biomarkers to reflect *dynamic* autophagy processes.

As discussed above, acute loss of mitochondrial membrane potential activates the PINK1-PRKN mitophagy pathway, in which OMM proteins become labeled with poly-ubiquitin chains including p-S65-Ub by the coordinated activities of PINK1 and PRKN. While measurement of non-modified PINK1 and PRKN may be useful in evaluating general protein stability in the contexts of disease-causing mutations, understanding protein activation status would be more informative to unraveling the *functional capability*. Measuring homeostatic PINK1 levels is a challenge due to the continual turnover of this ubiquitin kinase in absence of mitochondrial uncoupling. Yet, PRKN protein levels may be used as an indirect indicator of pathway activity as this ubiquitin ligase seems to be “consumed” with the degraded mitochondria; a complete loss of PINK1 stabilizes PRKN protein in its inactive form [365,490,491]. PINK1 and PRKN phosphorylation, with respect to PD-linked mutations, have been assessed *in vitro*, and efforts of developing quantitative and highly specific assays to measure these PTMs in PINK1 and PRKN are ongoing and would provide useful insights into the effect of mutations on activation status and signaling *in vivo.*

The mitophagy signal, p-S65-Ub, is a molecule specifically generated by the activity of PINK1 and represents < 1% of endogenous ubiquitin. The levels of p-S65-Ub increase during the normal aging process, and are higher in patient brains affected by AD [492], Dementia with Lewy Bodies [493] and PD [360], but not multiple system atrophy [494]. Increased levels of p-S65-Ub with aging are indicative of greater mitochondrial dysfunction requiring surveillance by the mitochondrial quality control system. Decreased p-S65-Ub may indicate low activation of this pathway, either due to low requirement for turnover (due to limited stress/damage) or mutation-associated disruption of p-S65-Ub production or mitochondrial labeling, or reflect engagement of PINK1-independent mitophagy pathways. Increased p-S65-Ub in the context of neurodegenerative disorders (compared to healthy individuals) may signal either impaired turnover or increased active turnover to compensate for pathology-associated mitochondrial stressors; therefore, understanding an individual’s pathologies (including other mutations in autophagy/mitophagy genes) is crucial for the accurate interpretation of p-S65-Ub levels. Recent and ongoing efforts have allowed for greater precision and specificity in measuring p-S65-Ub [495,496]. Levels of p-S65-Ub have been exploited for functional screening in cells and more recently as a quantitative trait in an autopsy brain genome-wide association study, resulting in the identification of disease risk and potential resilience factors [457,497,498]. While this biomarker is not informative concerning other mitophagy pathways (Table 1), levels of p-S65-Ub may also be used as a biomarker to track increased PINK1 activity in living systems in response to small molecules that inhibit its degradation [174] or promote its activity [499].

These additional modalities should be considered as they each provide unique insight into the activation state and status of proteins, allowing for more precise prognostic evaluation at the molecular level regulating autophagy activation and progression. Working toward the building of standardized panels that define PTMs in “key” autophagy genes via highly specific and sufficiently sensitive assays, and using them to evaluate large well-defined and well-controlled clinical cohorts would improve the precision by which autophagy *dynamics* can be described. This would of course require the generation and standardization of normal autophagy dynamics “benchmarks” in healthy men and women of different genetic backgrounds that could be compared to diseased samples. Such studies will improve our general understanding of how autophagy is compromised during various stages of neurodegeneration in a disease-specific manner and serve as screening tools to determine which treatments (i.e. what gene or part of the pathway to target) would best serve individuals suffering from neurological diseases.

#### Treatments

MTOR (mechanistic target of rapamycin kinase) is a component of the MTORC1 and MTORC2 complexes that negatively regulate autophagy by phosphorylation of ULK1 at its S757 inhibitory residue; MTOR is hyperactive in AD which inhibits autophagic clearance of pathologically associated protein aggregates [500]. Efforts to target overactive MTOR signaling have long been in development as a means of enhancing autophagic clearance of protein aggregates or damaged intracellular compartments. The evidence supporting rapamycin as a potential treatment for AD or other neurodegenerative disorders is mostly pre-clinical. A phase I clinical trial was successful in establishing safety among patients with AD [501], and there are currently two phase II studies still in the recruitment stage [502]. MTOR is also hyperactive in PD, HD, and ALS, suggesting that these populations may also benefit from rapamycin treatment [500].

Metformin [503] and trehalose [504] are thought to activate autophagy through activation of AMPK, not requiring inhibition of MTOR, but more recent evidence has also shown that trehalose also induces autophagy by activation of TFEB [505,506]. A 1–2 year treatment with metformin in normal and AD mice demonstrated cognitive improvement in mice younger than 16 months old, whereas longer treatments caused cognitive decline in healthy mice and mice with AD; the authors cautioned that care should be taken when considering metformin treatment in patients with AD and highlighted conflicting studies as to the safety and efficacy of metformin [507]. Other caveats regarding the use of metformin come from a large meta-analysis on its utility in preventing dementia in patients with or without diabetes (metformin is widely used as a treatment for diabetes), where the results suggest it may not be effective in the prevention of dementia in patients without diabetes [508]. Despite this, Metformin has been used to alleviate autophagic inhibition in animal models of PD and HD as well as in patients with AD, PD, and HD. In general animal models show improvement, but efficacy and side-effects in patients and animal models (such as reduced vitamin B12 levels) should be further explored to refine treatment with Metformin, or to better identify sub-populations that would benefit most from treatment [503].

Trehalose has been assessed in several animal models of neurodegenerative disease (PD, AD, HD, ALS) and found to reduce protein aggregates and rescue autophagy function. In a seminal article published in 2013, Trehalose prolonged lifespan, increased autophagy in motoneurons, and decreased aggregation of mutant SOD1 protein in an ALS mouse model, leading to another decade of research on MTOR-independent autophagy activation. The molecular characteristics, mechanism of action, and current and future disease indications for Trehalose therapies has been extensively reviewed [509]. Pre-clinical data demonstrates neuroprotection with limited pleiotropic affects in various disease models. Early phase clinical trials using Trehalose have demonstrated safety and improved cognitive and motor neuron functions (e.g., swallowing) [509]. A trial using trehalose to treat ALS demonstrated efficacy among individuals not taking RELYVRIO (another medication used to treat ALS that was recently discontinued after a failed phase III clinical trial; [https://clinicaltrials.gov/study/NCT05136885?tab=results#outcome-measures, last accessed 22 Aug 2025]). Taken together, trehalose seems to have promising therapeutic potential for neurodegenerative diseases in which autophagic function is disrupted.

Acting at the terminal step in autophagy, lysosomes regulate autophagic turnover and are dysregulated in several neurodegenerative diseases. Targeting lysosomal status is particularly important given the known detrimental consequences of driving autophagy initiation in cells with lysosomal impairment [192]. Lysosomes regulate autophagy via inactivation of MTOR during cellular nutrient deficiency, and lysosomal deacidification impairs the activity of pH sensitive lysosomal enzymes [456]. Pharmacological approaches to enhancing lysosomal acidification rescue autophagic degradation and prevent inflammation in mouse models of lysosomal storage disease [510,511]. Several drugs restore the acidic pH of the lysosome via modulation of the MCOLN/TRPML ion channel or V-ATPase activity [512], and delivery of nanoparticles that enhance lysosomal acidification are promising and under early development [513]. As nanoparticle medicines are highly heterogeneous and represent an evolving area in biology, these therapies may require additional regulatory considerations compared to “simple drugs” [514].

Inhibitors of LRRK2 activity (either pharmacological kinase inhibitors or anti-sense oligonucleotides that decrease LRRK2 expression) demonstrate promising results in pre-clinical and early phase clinical studies for PD. One early phase II study seeks to enhance lysosome function via activation of GBA and another phase II study in progress seeks to restore GBA function using adeno-associated-virus (AAV) gene therapy to replace mutant GBA in patients with PD [400]. Another gene therapy targeting lysosomes restores the nominal function of PGRN via delivery of functional PGRN, referred to as ALPHA-0602. This pre-clinical gene therapy is reported to restore PGRN levels *in vitro* and *in vivo*, protects mice against TARDBP/TDP-43 pathology, and reduces motor neuron toxicity in an ALS model [515]. ALPHA-0602 has an orphan drug designation in the United States (https://www.accessdata.fda.gov/scripts/opdlisting/oopd/detailedIndex.cfm?cfgridkey=725319, last accessed 22 Aug 2025), but currently there are no clinical studies recruiting for ALPHA-0602.

Other potential avenues for gene therapies include DJ-1, NDP52, PINK1, and PRKN. Introduction of wild-type DJ-1 seems to be neuroprotective in rats against neuronal death in a model of 6-hydroxydopamine induced PD, congruent with similar findings that pharmacologically targeting DJ-1 is protective in these contexts [516]. The discovery of a protective and more active NDP52 variant that interacts with autophagic machinery more readily compared to wild-type NDP52 implicates NDP52 as a novel gene therapy target for AD [371]. Another potential target for gene therapy is the mitophagy associated kinase PINK1, where *in vitro* work demonstrates enhanced production of p-S65-Ub by a G411A mutant associated with increased mitochondrial turnover [368]. Although this PINK1 variant has not been identified in a patient, the neuroprotective effects it confers may be able to limit the neuropathology or may provide resilience. Animal models of AD and HD show enhanced neuroprotection with AAV-PINK1 overexpression or germline editing [172,173,517]. Similarly, models of PD and AD show that overexpression of PRKN (by means of AAV, lentivirus, germline editing, or introduction of membrane permeable PRKN) reduces cellular pathology and protects against disease progression [518–522]. Considering the many known autophagy and autophagy-related genes with causal mutations in disease progression that perturb normal function, and the major advances in gene editing technology over the last 20 years, additional targets of autophagy may be considered. The benefit of targeting *autophagy* as a mechanism, especially with causal genes implicated in multiple disorders (e.g., SNX14), is that one gene therapy could show applicability across multiple diseases, potentially allowing for more rapid translation of such treatments.

Mitophagy can also be enhanced pharmacologically, and several strategies target PINK1, PRKN, USP30, and SQSTM1– a few of which have recently entered clinical trials [523]. Urolithin A is a naturally occurring metabolite that stimulates mitophagy by promoting PINK1 and PRKN activity by a mechanism that is not entirely understood [524]. Safety was demonstrated in healthy individuals after *in vivo* testing showed prolonged lifespan in *C. elegans* and improved muscle function in rodents [525,526]. Urolithin A has also been shown to improve multiple cognitive measures and to reduce neuropathology in mouse models of AD, physiological aging and a model of macular degeneration [527,528]. One of the earliest small molecules targeting PINK1, kinetin triphosphate, is an ATP analog that was initially shown to increase both a mutant and wild-type PINK1 activity *in vitro* [499]; although direct binding of kinetin triphosphate to PINK1 was recently questioned [529]. Inhibiting the rapid degradation of PINK1 is another strategy to elevate the bioavailability of this neuroprotective kinase. The small molecule BC1464 interferes with the interaction of PINK1 with the ubiquitin ligase FBXO7, elevating PINK1 expression and p-S65-Ub levels, and protecting against cell death and dendritic atrophy in chemical and genetic models of PD [174]. Another small molecule, BIO-2007817, promotes PRKN activity via direct interaction and can rescue the activity of some disease mutants [530,531]. Other small molecule activators of PRKN were identified in a high-content screen and found to exert their action via inhibition of USP30 [532]. Deubiquitinases, such as USP30, inhibit PINK1- and PRKN-mediated mitophagy by the deconjugation of ubiquitin chains on OMM species, thus limiting the ability of ubiquitin-dependent mitophagy adaptors to tether OMM proteins to the forming autophagosome. Inhibition of USP30 enhances mitophagy *in vitro* and can rescue p-S65-Ub levels in fibroblasts with pathogenic PRKN mutations [533,534]. Knockout or inhibition of USP30 protects against loss of dopaminergic neurons in a mouse model of PD [535]. Similarly, inhibition of USP14 (another deubiquitinase) can rescue mitophagy activity *in vitro*, but apparently in a PRKN-independent fashion [39]. The autophagy receptor SQSTM1 has also been targeted to enhance mitophagy *in vitro* [536]. One caveat to consider is that each of these proteins have additional functions beyond autophagy, and it may be difficult to ascribe beneficial or detrimental impacts on one particular pathway.

Last, it would be negligent to disregard the impact of lifestyle choices on disease progression. Caloric restriction (CR) has been long recognized as major factor that influences brain aging and the cognitive decline associated with neurodegeneration [537]. By nature of the low nutrient conditions under which autophagy is activated, restricting food intake stimulates autophagy [538]. A thorough review summarizes the effects of caloric restriction on various animal models of neurodegeneration and reports that CR regulates neuroinflammation in neurodegenerative diseases [539]. Another article discusses how CR reduces symptomology and disease progression in various disorders [540]. A recent report demonstrates that 40% CR led to a 9 month ( > 36%) increase in lifespan compared to mice that were fed *ad libitum* [541]. One caveat to consider is the degree of CR that may be necessary to induce autophagy in the brain. In mice, 48 h of complete starvation does not induce brain autophagy [542], most likely because responses in other peripheral tissues act to support the nutritive status of the brain. While neurons in culture do respond to nutrient deprivation by upregulating autophagy, these responses are more robust in male neurons, with female neurons showing primarily lipid mobilization [543]. Thus, beneficial impacts of CR may involve multiple mechanisms.

Another factor known to prolong lifespan in humans is exercise. Unsurprisingly, exercise stimulates autophagy and is thought to be neuroprotective, both for healthy aging and in the context of neurodegenerative disease [544,545]. Exercise may also be beneficial for reducing age-related cellular senescence [546]. Again, most of these studies are correlative, showing upregulation of autophagy coincident with the beneficial effect being studied. In aged mice, exercise-related improvements in mitochondrial function were not linked to increased autophagy, but to changes in mitochondrial fission [547]. Nevertheless, as CR and exercise are generally accessible despite factors such as socioeconomic status, which may limit patient access to certain therapies, they should be considered and further studied as a means of ameliorating or delaying symptom onset in susceptible families and individuals.

## Conclusions and future perspectives

In the central and peripheral nervous system, cellular quality control mechanisms face unique challenges due to the longevity of individual neurons and the architectural, metabolic and functional specializations necessary for proper brain function. It is thus not surprising that numerous gene mutations associated with neurodevelopmental and neurodegenerative diseases have been implicated in every step of autophagy (Figure 1).

A growing number of *in vitro* and *in vivo* studies implicate autophagy and mitophagy in normal synaptic development and function. Synapses are enriched in autophagy mediators, and localized induction of autophagy occurs in response to synaptic activity. While the impact of autophagy impairment on synaptic tuning and plasticity, which underlie learning, memory, sensation, motor control, and cognition, have been demonstrated, the particular mechanisms by which autophagy- or mitophagy-linked proteins regulate synaptic activity are still being elucidated. Emerging themes include degradation/trafficking of neurotransmitter receptors, degradation of proteins that regulate components of the synaptic vesicle cycle, pre- and postsynaptic membrane trafficking, and vesicular crosstalk with the endocytic system. Future work is needed to elucidate how nuances in neuronal stimulation translate into distinguishing degradative and secretory functions downstream of autophagy induction.

Mitochondria are particularly important for maintaining neuronal structure, health and function, with particularly important roles in supporting both pre- and postsynaptic activity. Not surprisingly, numerous disease-associated mutations target proteins that are implicated in regulating the mitochondrial life cycle, including mitophagy. PINK1 and PRKN play important roles in regulating one of a growing number of cargo recognition pathways for mitophagy. These proteins also play important roles in regulating synaptic protein turnover, excitotoxicity, mitochondrial biogenesis, fission-fusion-transport dynamics, and calcium homeostasis, through both shared and distinct pathways. The newly described role of PINK1 in regulating dendritic spine maturation and electrophysiology *in vitro* and spine density *in vivo* likely involves both mitochondrial-dependent and -independent signaling functions [13].

While the link between autophagy dysfunction and neurodevelopmental disorders and neurodegenerative diseases is evident, further research is necessary to elucidate the precise mechanisms connecting alterations in autophagy- and mitophagy-linked processes and proteins to dysregulated neuronal function and survival. In particular, the specific impact of disease related alterations in protein and organellar quality control on synaptic health, structure and function remain to be elucidated. Nevertheless, the knowledge that autophagy is disrupted in these diseases opens the door for autophagy-targeting therapies, which have thus far resulted in mixed success. Advances in understanding the basic biochemistry and cell biology driving the molecular pathogenesis of specific diseases should be leveraged to generate and test new biomarkers that will allow for earlier treatments and individualized prognostic and theranostic predictions.

## Supplementary Material

Author relevant publications.pdf

## Data Availability

Data sharing is not applicable to this article as no new data were created or analyzed in this study.
