# Supplementary material for: Autophagy and mitophagy at the synapse and beyond: implications for learning, memory and neurological disorders
Source: Autophagy. 2025 Nov 23;22(1):10–52. doi: 10.1080/15548627.2025.2581217 (PMC12758295; doi:10.1080/15548627.2025.2581217)
Supplement: Author relevant publications.pdf [file KAUP_A_2581217_SM7038.pdf]

List of Relevant Publications by each co-author

Jiayi Lu – Relevant Works

Peer Reviewed Publications

Z Xu, **J Lu**, S Gao, YN Rui. (2024) THSD1 Suppresses Autophagy-Mediated Focal Adhesion Turnover by Modulating the FAK-Beclin 1 Pathway. *Int J Mol Sci* **25:** 2139.

Reviews

J Lu, B Linares, Z Xu, YN Rui. (2021) Mechanisms of FA-Phagy, a New Form of Selective Autophagy/Organellophagy. *Front Cell Dev Biol* **9:** 799123.

Damian Di Florio – Relevant Works

Peer Reviewed Publications

Baninameh Z, Watzlawik JO, Hou X, Richardson T, Kurchaba NW, Yan T, **Di Florio DN**, Fairweather D, Kang L, Nguyen JH, Kanekiyo T, Dickson DW, Noda S, Sato S, Hattori N, Goldberg MS, Ganley IG, Stauch KL, Fiesel FC, Springer W. Alterations of PINK1-PRKN signaling in mice during normal aging. Autophagy Rep. 2024; 3 (1) Epub 2024 Dec 07 PMID: 40008113 PMCID: PMC11855339 DOI: 10.1080/27694127.2024.2434379

Reviews

Beetler DJ#, **Di Florio DN#,** Law EW, Groen CM, Windebank AJ, Peterson QP, Fairweather D. The evolving regulatory landscape in regenerative medicine. Mol Aspects Med. 2023 Jun; 91:101138 Epub 2022 Aug 29 PMID: 36050142 PMCID: PMC10162454 DOI: 10.1016/j.mam.2022.101138

Di Florio DN, Sin J, Coronado MJ, Atwal PS, Fairweather D. Sex differences in inflammation, redox biology, mitochondria and autoimmunity. Redox Biol. 2020 Apr; 31:101482 Epub 2020 Mar 04 PMID: 32197947 PMCID: PMC7212489 DOI: 10.1016/j.redox.2020.101482

Patricia Boya - Relevant Works (last five years)

128. How crosstalk between mitochondria, lysosomes, and other organelles can prevent or promote dry age-related macular degeneration.

Lakkaraju A, **Boya P**, Csete M, Ferrington DA, Hurley JB, Sadun AA, Shang P, Sharma R, Sinha D, Ueffing M, Brockerhoff SE.Exp Eye Res. 2024 Dec 22;251:110219. doi: 10.1016/j.exer.2024.110219. Online ahead of print.PMID: 39716681

127. Women in Autophagy, an initiative to promote gender parity in science.

McCabe M, **Boya P,** Chen R-H, Chu C, Colombo M, Delgui L, Eskeline EL, Hamasaki M, Hansen M, He C, Jäättelä M, Kimchi, Kraft C, Kundu M, Melendez A, Pattingre S, Proikas-Cezanne T, Sebti S, Simon K, Simonsen A, Tooze S, Vaccaro M., Wang X, White E, Zhao Y,, Cuervo A.M.

Nature Cell Biology. NCB-COM55810A, *in press.*

126. Targeted proteomics addresses selectivity and complexity of protein degradation by **autophagy**.

Leytens A, Benítez-Fernández R, Jiménez-García C, Robaty C, Stumpe M, **Boya P**, Dengjel J.

*Autophagy***.** 2024 Sep 20:1-16. doi: 10.1080/15548627.2024.2396792. Online ahead of print.

125. Fast and quantitative mitophagy assessment by flow cytometry using the *mito*-QC reporter.

Jiménez-Loygorri JI, Jiménez-García C, Viedma-Poyatos Á, **Boya P.**

*Front Cell Dev Bio***l**. 2024 Sep 11;12:1460061. doi: 10.3389/fcell.2024.1460061.

124. Recycling the recyclers: lysophagy emerges as a new pharmacological target for retinal degeneration.

Jiménez-Loygorri JI, **Boya P.**

*Autophag*y. 2024 Nov;20(11):2589-2590. doi: 10.1080/15548627.2024.2391726.

123. Urolithin A promotes p62-dependent lysophagy to prevent acute retinal neurodegeneration.

Jiménez-Loygorri JI, Viedma-Poyatos Á, Gómez-Sintes R, **Boya P.**

Mol Neurodegener. 2024 Jun 18;19(1):49. doi: 10.1186/s13024-024-00739-3.

122. Aging STINGs: mitophagy at the crossroads of neuroinflammation.

Jiménez-Loygorri JI, **Boya P**.

Autophagy. 2024 Mar 6:1-3. doi: 10.1080/15548627.2024.2322421.

121. Mitophagy curtails cytosolic mtDNA-dependent activation of cGAS/STING inflammation during aging.

Jiménez-Loygorri JI, Villarejo-Zori B, Viedma-Poyatos A, Zapata-Muñoz J, Benítez-Fernández R, Frutos-Lisón MD, Tomás-Barberán FA, Espín JC, Area-Gómez E, Gomez-Duran A, **Boya P**.

Nat Commun. 2024 Jan 27;15(1):830. doi: 10.1038/s41467-024-45044-1.

120. Lysosomes in retinal health and disease.

Boya P, Kaarniranta K, Handa JT, Sinha D.

*Trends Neurosci***.** 2023 Dec;46(12):1067-1082. doi: 10.1016/j.tins.2023.09.006. Invited review.

119. Transcriptomics and translatomics identify a robust inflammatory gene signature in brain endothelial cells after ischemic stroke.

Arbaizar-Rovirosa M, Gallizioli M, Lozano JJ, Sidorova J, Pedragosa J, Figuerola S, Chaparro-Cabanillas N, Boya P, Graupera M, Claret M, Urra X, Planas AM.

*J Neuroinflammation***.** 2023 Sep 11;20(1):207. doi: 10.1186/s12974-023-02888-6.

118. Mitophagy in the retina: Viewing mitochondrial homeostasis through a new lens.

Jiménez-Loygorri JI, Benítez-Fernández R, Viedma-Poyatos Á, Zapata-Muñoz J, Villarejo-Zori B, Gómez-Sintes R, **Boya P***.

Prog Retin Eye Res. 2023 Jul 15;96:101205. doi: 10.1016/j.preteyeres.2023.101205. Review.

117. Bacteria-instructed B cells cross-prime naïve CD8+ T cells triggering effective cytotoxic responses.

García-Ferreras R, Osuna-Pérez J, Ramírez-Santiago G, Méndez-Pérez A, Acosta-Moreno AM, Del Campo L, Gómez-Sánchez MJ, Iborra M, Herrero-Fernández B, González-Granado JM, Sánchez-Madrid F, Carrasco YR, Boya P, Martínez-Martín N, Veiga E.

EMBO Rep. 2023 May 15:e56131. doi: 10.15252/embr.202256131. Online ahead of print.

116. Apoptotic cell death in disease-Current understanding of the NCCD 2023.

Vitale I, …., Boya P,

Cell Death Differ. 2023 May;30(5):1097-1154. doi: 10.1038/s41418-023-01153-w. Review

115. Autophagy in the eye: from physiology to pathophysology.

Liton PB, Boesze-Battaglia K, Boulton ME, Boya P, Ferguson TA, Ganley IG, Kauppinnen A, Laurie GW, Mizushima N, Morishita H, Russo R, Sadda J, Shyam R, Sinha D, Thompson DA, Zacks DN.

*Autophagy Rep***.** 2023;2(1):2178996. doi: 10.1080/27694127.2023.2178996. Review

114. Identification of a new structural family of SGK1 inhibitors as potential neuroprotective agents.

Maestro I, Madruga, E, **Boya P**, Martínez A.

*J Enzyme Inhib Med Che*m (2023) 38(1): 2153841.

113. Microglial phagocytosis dysfunction in stroke is driven by energy depletion and induction of autophagy.

Beccari S, Sierra-Torre V, Valero J, Pereira-Iglesias M, García-Zaballa M, Soria FN, De Las Heras-Garcia L, Carretero-Guillen A, Capetillo-Zarate E, Domercq M, Huguet PR, Ramonet D, Osman A, Han W, Dominguez C, Faust TE, Touzani O, Pampliega O, **Boya P**, Schafer D, Mariño G, Canet-Soulas E, Blomgren K, Plaza-Zabala A, Sierra A.

Autophagy. 2023 Jul;19(7):1952-1981. doi: 10.1080/15548627.2023.2165313.

112. Carbon Monoxide stimulates both mitophagy and mitochondrial biogenesis to mediate protection against oxidative stress in astrocytes.

Figueiredo-Pereira C, Villarejo-Zori B, Cipriano PC, Tavares D, Ramírez-Pardo I, **Boya P**, Vieira HLA.

Mol Neurobiol. 2022 Nov 15. doi: 10.1007/s12035-022-03108-7.

111. Discovery of Mitophagy Inhibitors with Therapeutic Potential in Different Familial Amyotrophic Lateral Sclerosis Mutations.

Maestro I, de la Ballina LR, Porras G, Corrochano S, De Lago E, Simonsen A, **Boya P*, Martinez A*.**

Int J Mol Sci. 2022 Oct 21;23(20):12676. doi: 10.3390/ijms232012676.

110. BNIP3L/NIX regulates both mitophagy and pexophagy.

Wilhelm LP, Zapata-Muñoz J, Villarejo-Zori B, Pellegrin S, Freire CM, Toye AM, **Boya P**, Ganley IG.

EMBO J. 2022 Oct 10:e111115. doi: 10.15252/embj.2022111115.

109. The mechanism of macroautophagy: The movie.

Reggiori F, **Boya P**, da Costa D, Elazar Z, Eskelinen EL, Farrés J, Guettler S, Kraft C, Jungbluth H, Martinez A, Morel E, Pless O, Proikas-Cezanne T, Simonsen A.

Autophagy Rep. 2022 Sep 14;1(1):414-417. doi: 10.1080/27694127.2022.2096115.

108. Ambra1 haploinsufficiency in CD1 mice results in metabolic alterations and exacerbates age-associated retinal degeneration.

Ramírez-Pardo I, Villarejo-Zori B, Jiménez-Loygorri JI, Sierra-Filardi E, Alonso-Gil S, Mariño G, de la Villa P, Fitze PS, Fuentes JM, García-Escudero R, Ferrington DA, Gomez-Sintes R, **Boya P*.**

*Autophag*y. 2022 Jul 24:1-21. doi: 10.1080/15548627.2022.2103307.

107. Targeting retinoic acid receptor alpha-corepressor interaction activates chaperone-mediated autophagy and protects against retinal degeneration.

Gomez-Sintes R, Xin Q, Jimenez-Loygorri JI, McCabe M, Diaz A, Garner TP, Cotto-Rios XM, Wu Y, Dong S, Reynolds CA, Patel B, de la Villa P, Macian F, **Boya P*, Gavathiotis E*, Cuervo AM*.**

Nat Commun. 2022 Jul 21;13(1):4220. doi: 10.1038/s41467-022-31869

106. p38 MAPK priming boosts VSMC proliferation and arteriogenesis by promoting PGC1α-dependent mitochondrial dynamics.

Sahún-Español Á, Clemente C, Jiménez-Loygorri JI, Sierra-Filardi E, Herrera-Melle L, Gómez-Durán A, Sabio G, Monsalve M, **Boya P**, Arroyo AG.

Sci Rep. 2022 Apr 8;12(1):5938. doi: 10.1038/s41598-022-09757-x

105. Apoptosis-Inducing Factor Deficiency Induces Tissue-Specific Alterations in Autophagy: Insights from a Preclinical Model of Mitochondrial Disease and Exercise Training Effects

Laine-Menéndez S, Fernández-de la Torre M, Fiuza-Luces C, Delmiro A, Arenas J, Martín MA, **Boya P**, Lucia A, Morán M.

Antioxidants (Basel). 2022 Mar 7;11(3):510. doi: 10.3390/antiox11030510.

104. Phenotypic Assay Leads to Discovery of Mitophagy Inducers with Therapeutic Potential for Parkinson's Disease.

Maestro I, de la Ballina LR, Simonsen A**, Boya P*, Martinez A*.**

ACS Chem Neurosci. 2021 Dec 15;12(24):4512-4523. doi: 10.1021/acschemneuro.1c00529.

103. Autophagy and disease: new insights and challenges ahead.

Boya P.

Mol Aspects Med. 2021 Dec;82:101047. doi: 10.1016/j.mam.2021.101047. Issue introduction.

102. New insights into the role of autophagy in retinal and eye diseases.

Villarejo-Zori B, Jiménez-Loygorri JI, Zapata-Muñoz J, Bell K, **Boya P*.**

Mol Aspects Med. 2021 Oct 4:101038. doi: 10.1016/j.mam.2021.101038.

IF2020:14.3, Q1, D1

101. Autophagy in major human diseases

Klionsky DJ, Petroni G, Amaravadi RK, Baehrecke EH, Ballabio A, **Boya P**, Bravo-San Pedro JM, Cadwell K, Cecconi F, Choi AMK, Choi ME, Chu CT, Codogno P, Colombo MI, Cuervo AM, Deretic V, Dikic I, Elazar Z, Eskelinen EL, Fimia GM, Gewirtz DA, Green DR, Hansen M, Jäättelä M, Johansen T, Juhász G, Karantza V, Kraft C, Kroemer G, Ktistakis NT, Kumar S, Lopez-Otin C, Macleod KF, Madeo F, Martinez J, Meléndez A, Mizushima N, Münz C, Penninger JM, Perera RM, Piacentini M, Reggiori F, Rubinsztein DC, Ryan KM, Sadoshima J, Santambrogio L, Scorrano L, Simon HU, Simon AK, Simonsen A, Stolz A, Tavernarakis N, Tooze SA, Yoshimori T, Yuan J, Yue Z, Zhong Q, Galluzzi L, Pietrocola F.

EMBO J. 2021 Oct 1;40(19):e108863. doi: 10.15252/embj.2021108863. Review.

IF2020: 12,9,Q1, D1

100. Towards a better understanding of the neurodevelopmental role of autophagy in sickness and in health

Zapata-Muñoz J, Villarejo-Zori B, Largo-Barrientos P, **Boya P*.**

Cell Stress. 2021 Jun 29;5(7):99-118. doi: 10.15698/cst2021.07.253. Review

99. Autophagy induction during stem cell activation plays a key role in salivary gland self-renewal.
Orhon I, Rocchi C, Villarejo-Zori B, Serrano Martinez P, Baanstra M, Brouwer U, **Boya P*,** Coppes R*, Reggiori F*.

Autophagy, 2021 May 19;1-16.

IF2020: 16.0,Q1, D1

98. Lipid dismantling of lens organelles for clear vision.

Boya P.

Nature. 2021 Apr;592(7855):509-510. doi: 10.1038/d41586-021-00888-1. News and views.

IF2019: 42.8, Q1, D1

97. Molecular Alterations in Sporadic and *SOD1*-ALS Immortalized Lymphocytes: Towards a Personalized Therapy.

Lastres-Becker I, Porras G, Arribas-Blázquez M, Maestro I, Borrego-Hernández D, **Boya P**, Cerdán S, García-Redondo A, Martínez A, Martin-Requero Á.

Int J Mol Sci. 2021 Mar 16;22(6):3007. doi: 10.3390/ijms22063007.

IF2019: 4.6, Q1

96. Guidelines for the use and interpretation of assays for monitoring **autophagy** (4th edition).

Klionsky Dj, ….Boya P,… Gomez-Sintes R, …., Ramirez-Pardo I, ….Villarejo-Zori B, …

Autophagy. 2021 Feb 8:1-382. doi: 10.1080/15548627.2020.1797280. Review

IF2019:9.8, Q1

95. Regulation of PRKN-independent mitophagy.

Terešak P, Lapao A, Subic N, **Boya P**, Elazar Z, Simonsen A.

Autophagy. 2021 Feb 25:1-16. doi: 10.1080/15548627.2021.1888244.

IF2019:9.8, Q1

94. HDAC inhibition ameliorates cone survival in retinitis pigmentosa mice.

Samardzija M*, Corna A*, Raquel Gomez-Sintes R*, Ali Jarboui M, Armento A, Roger JE, Petridou E, Haq W, Paquet-Durand F, Zrenner E, de la Villa P, Zeck G, Grimm C, **Boya P**, Ueffing M and Trifunovic D.

Cell Death Differ. 2021 Apr;28(4):1317-1332 doi.org/10.1038/s41418-020-00653-3

IF2019:10.7, Q1, D1

93. Caty Casas (1967-2020).

Boya P.

Autophagy. 2020 Oct 12:1-3. doi: 10.1080/15548627.2020.1822092.

IF2019:9.8, Q1

92. HIF1α or mitophagy: which drives cardiomyocyte differentiation?

Villarejo-Zori B, Jiménez-Loygorri JI, Boya P. Cell Stress. 2020 May 11;4(5):95-98.

doi: 10.15698/cst2020.05.219.

91. Age related Retinal Ganglion Cell susceptibility in context of autophagy deficiency.

Bell K, Rosignol I, Sierra-Filardi E, Rodríguez-Muela N, Schmelter C, Cecconi F, Grus F, **Boya P.**

Cell Death and Discovery, 2020 Apr 17;6:21. doi: 10.1038/s41420-020-0257-4.

IF2019: 4.1

90. The mito-QC reporter for quantitative mitophagy assessment in primary retinal ganglion cells and experimental glaucoma models.
Rosignol I, Villarejo-Zori B, Teresak P, Sierra-Filardi E, Pereiro X, Rodríguez-Muela N, Vecino E, Vieira HLA, Bell K, **Boya P.**

IJMS, 2020 Mar 10;21(5). pii: E1882. doi: 10.3390/ijms21051882,

IF2019:4.2, Q2

89. Serum- and glucocorticoid-induced kinase 1, a new therapeutic target for autophagy modulation in chronic diseases.

Maestro I, **Boya P**, Martinez A.

Expert Opin Ther Targets. 2020 Feb 18:1-13. doi: 10.1080/14728222.2020.1730328.

IF2018:4,6, Q1

88. Acyl-CoA-Binding Protein Is a Lipogenic Factor that Triggers Food Intake and Obesity.

Bravo-San Pedro JM, Sica V, Martins I, Pol J, Loos F, Maiuri MC, Durand S, Bossut N, Aprahamian F, Anagnostopoulos G, Niso-Santano M, Aranda F, Ramírez-Pardo I, Lallement J, Denom J, Boedec E, Gorwood P, Ramoz N, Clément K, Pelloux V, Rohia A, Pattou F, Raverdy V, Caiazzo R, Denis RGP, **Boya P**, Galluzzi L, Madeo F, Migrenne-Li S, Cruciani-Guglielmacci C, Tavernarakis N, López-Otín C, Magnan C, Kroemer G.

Cell Metab. 2019;30(4):754-767.e9.

IF2018:22.4, Q1, D1.

Sandra Maday - relevant works

Peer-reviewed primary publications

Jimenez-Cyrus, D., V.S. Adusumilli, M.H. Stempel, **S. Maday**, G. Ming, H. Song, and A.M. Bond. 2024. Molecular cascade reveals sequential milestones underlying hippocampal neural stem cell development into an adult state. *Cell Reports*. 43: 114339.

Yuan, R., Y. Hahn, M.H. Stempel, D.K. Sidibe, O. Laxton, J. Chen, A. Kulkarni, and **S. Maday**. 2023. Proteasomal inhibition preferentially stimulates lysosome activity relative to autophagic flux in primary astrocytes. *Autophagy.* 19: 570-596.

Sidibe, D.K., V.V. Kulkarni, A. Dong, J.B. Herr, M.C. Vogel, M.H. Stempel, and **S. Maday**. 2022. Brain-derived neurotrophic factor stimulates the retrograde pathway for axonal autophagy. *Journal of Biological Chemistry.* 298: 102673.

Kulkarni, V.V., M.H. Stempel, A. Anand, D.K. Sidibe, and **S. Maday**. 2022. Retrograde axonal autophagy and endocytic pathways are parallel and separate in neurons. *Journal of Neuroscience.* 42: 8524-8541.

Kulkarni, V.V., A. Anand, J.B. Herr, C. Miranda, M.C. Vogel, and **S. Maday.** 2021. Synaptic activity controls autophagic vacuole motility and function in dendrites. *Journal of Cell Biology.* 220 (6): e202002084.

Kulkarni, A., A. Dong, V.V. Kulkarni, J. Chen, O. Laxton, A. Anand, and **S. Maday**. 2020. Differential regulation of autophagy during metabolic stress in astrocytes and neurons. *Autophagy.* 16: 1651-1667.

Reviews

Smith, E.M., Coughlan, M.L., and **S. Maday**. 2025. Turning garbage into gold: Autophagy in synaptic function. *Current Opinion in Neurobiology*. 90: 102937.

Coughlan, M.L. and **S. Maday.** 2023. Beyond housekeeping: autophagy regulates PKA signaling at synapses. *Trends in Neurosciences.* 46: 167-169.

Kulkarni, V.V. and **S. Maday.** 2023. Sorting trash from treasure: separate pathways for autophagy and endocytic trafficking in axons. *Autophagy Reports.* 2: 2166322.

Sidibe, D.K., M.C. Vogel, and **S. Maday**. 2022. Organization of the autophagy pathway in neurons. *Current Opinion in Neurobiology.* 75: 102554.

Vogel, M.C., and **S. Maday**. 2022. Autophagy: Identification of MTMR5 as a neuron-enriched suppressor. *Current Biology*. 32: R574-R577.

Winckler, B, V. Faundez, **S. Maday**, Q. Cai, C.G. Almeida, and H. Zhang. 2018. The endolysosomal system and proteostasis: from development to degeneration. *Journal of Neuroscience*. 38: 9364-9374.

Kulkarni, V.V., and **S. Maday**. 2018. Neuronal endosomes to lysosomes: A journey to the soma. *The Journal of Cell Biology*. 217: 2977-2979.

Kulkarni, A., J. Chen, and **S. Maday.** 2018. Neuronal autophagy and intercellular regulation of homeostasis in the brain. *Current Opinion in Neurobiology.* 51: 29-36.

Kulkarni, V.V. and **S. Maday**. 2018. Compartment-specific dynamics and functions of autophagy in neurons. *Developmental Neurobiology.* 78: 298-310.

Maday, S. 2016. Mechanisms of neuronal homeostasis: autophagy in the axon. *Brain Research.* 1649: 143-150.

Book chapters

Dong, A., V.V. Kulkarni, and **S. Maday**. 2019. Methods for imaging autophagosome dynamics in primary neurons. *Methods in Molecular Biology.* 1880: 243-256.

Wolfdieter Springer – Relevant Works

Peer Reviewed Publications

Springer W, Hoppe T, Schmidt E, Baumeister R. A Caenorhabditis elegans Parkin mutant with altered solubility couples alpha-synuclein aggregation to proteotoxic stress. Hum Mol Genet. 2005 Nov 15; 14(22):3407-23. Epub 2005 Oct 04. PMID: 16204351 DOI: 10.1093/hmg/ddi371

Hasegawa T, Treis A, Patenge N, Fiesel FC, **Springer W**, Kahle PJ. Parkin protects against tyrosinase-mediated dopamine neurotoxicity by suppressing stress-activated protein kinase pathways. J Neurochem. 2008 Jun; 105(5):1700-15. Epub 2008 Feb 04. PMID: 18248610 DOI: 10.1111/j.1471-4159.2008.05277.x

Klein CL, Rovelli G, **Springer W**, Schall C, Gasser T, Kahle PJ. Homo- and heterodimerization of ROCO kinases: LRRK2 kinase inhibition by the LRRK2 ROCO fragment. J Neurochem. 2009 Nov; 111(3):703-15. Epub 2009 Aug 27. PMID: 19712061 DOI: 10.1111/j.1471-4159.2009.06358.x

Fiesel FC, Voigt A, Weber SS, Van den Haute C, Waldenmaier A, Gorner K, Walter M, Anderson ML, Kern JV, Rasse TM, Schmidt T, **Springer W**, Kirchner R, Bonin M, Neumann M, Baekelandt V, Alunni-Fabbroni M, Schulz JB, Kahle PJ. Knockdown of transactive response DNA-binding protein (TDP-43) downregulates histone deacetylase 6. EMBO J. 2010 Jan 6; 29(1):209-21. Epub 2009 Nov 12. PMID: 19910924 PMCID: PMC2808372 DOI: 10.1038/emboj.2009.324

Geisler S, Holmstrom KM, Skujat D, Fiesel FC, Rothfuss OC, Kahle PJ, **Springer W**. PINK1/Parkin-mediated mitophagy is dependent on VDAC1 and p62/SQSTM1. Nat Cell Biol. 2010 Feb; 12(2):119-31. Epub 2010 Jan 24. PMID: 20098416 DOI: 10.1038/ncb2012

Cornejo Castro EM, Waak J, Weber SS, Fiesel FC, Oberhettinger P, Schutz M, Autenrieth IB, **Springer W**, Kahle PJ. Parkinson's disease-associated DJ-1 modulates innate immunity signaling in Caenorhabditis elegans. J Neural Transm. 2010 May; 117(5):599-604. Epub 2010 Apr 08. PMID: 20376509 DOI: 10.1007/s00702-010-0397-4

Geisler S, Holmstrom KM, Treis A, Skujat D, Weber SS, Fiesel FC, Kahle PJ, **Springer W**. The PINK1/Parkin-mediated mitophagy is compromised by PD-associated mutations. Autophagy. 2010 Oct; 6(7):871-8. Epub 2010 Oct 03. PMID: 20798600

Lincoln S, Allen M, Cox CL, Walker LP, Malphrus K, Qiu Y, Nguyen T, Rowley C, Kouri N, Crook J, Pankratz VS, Younkin S, Younkin L, Carrasquillo M, Zou F, Abdul-Hay SO, **Springer W**, Sando SB, Aasly JO, Barcikowska M, Wszolek ZK, Lewis JM, Dickson D, Graff-Radford NR, Petersen RC, Eckman E, Younkin SG, Ertekin-Taner N. LRRTM3 interacts with APP and BACE1 and has variants associating with late-onset Alzheimer's disease (LOAD). PLoS One. 2013; 8 (6):e64164 Epub 2013 June 04 PMID: 23750206 PMCID: PMC3672107 DOI: 10.1371/journal.pone.0064164

Hans F, Fiesel FC, Strong JC, Jackel S, Rasse TM, Geisler S, **Springer W**, Schulz JB, Voigt A, Kahle PJ. UBE2E ubiquitin-conjugating enzymes and ubiquitin isopeptidase Y regulate TDP-43 protein ubiquitination. J Biol Chem. 2014 Jul 4; 289(27):19164-79. Epub 2014 May 13. PMID: 24825905 PMCID: PMC4081952 DOI: 10.1074/jbc.M114.561704

Shannon B, Soto-Ortolaza A, Rayaprolu S, Cannon HD, Labbe C, Benitez BA, Choi J, Lynch T, Boczarska-Jedynak M, Opala G, Krygowska-Wajs A, Barcikowska M, Van Gerpen JA, Uitti RJ, **Springer W**, Cruchaga C, Wszolek ZK, Ross OA. Genetic variation of the retromer subunits VPS26A/B-VPS29 in Parkinson's disease. Neurobiol Aging. 2014 Aug; 35 (8):1958.e1-2 Epub 2014 Mar 05 PMID: 24684791 PMCID: PMC4023811 DOI: 10.1016/j.neurobiolaging.2014.03.004

Tacik P, Fiesel FC, Fujioka S, Ross OA, Pretelt F, Castaneda Cardona C, Kidd A, Hlavac M, Raizis A, Okun MS, Traynor S, Strongosky AJ, **Springer W**, Wszolek ZK. Three families with Perry syndrome from distinct

parts of the world. Parkinsonism Relat Disord. 2014 Aug; 20 (8):884-8 Epub 2014 May 13 PMID: 24881494 PMCID: PMC4125456 DOI: 10.1016/j.parkreldis.2014.05.004

Fiesel FC, Moussaud-Lamodiere EL, Ando M, **Springer W**. A specific subset of E2 ubiquitin-conjugating enzymes regulate Parkin activation and mitophagy differently. J Cell Sci. 2014 Aug 15; 127 (Pt 16):3488-504 Epub 2014 June 13 PMID: 24928900 PMCID: PMC4132391 DOI: 10.1242/jcs.147520

Siuda J, Jasinska-Myga B, Boczarska-Jedynak M, Opala G, Fiesel FC, Moussaud-Lamodiere EL, Scarffe LA, Dawson VL, Ross OA, **Springer W**, Dawson TM, Wszolek ZK. Early-onset Parkinson's disease due to PINK1 p.Q456X mutation--clinical and functional study. Parkinsonism Relat Disord. 2014 Nov; 20 (11):1274-8 Epub 2014 Sept 02 PMID: 25226871 PMCID: PMC4253017 DOI: 10.1016/j.parkreldis.2014.08.019

Caulfield TR, Fiesel FC, Moussaud-Lamodiere EL, Dourado DF, Flores SC, **Springer W**. Phosphorylation by PINK1 releases the UBL domain and initializes the conformational opening of the E3 ubiquitin ligase Parkin. PLoS Comput Biol. 2014 Nov; 10 (11):e1003935 Epub 2014 Nov 06 PMID: 25375667 PMCID: PMC4222639 DOI: 10.1371/journal.pcbi.1003935

Yue M, Hinkle KM, Davies P, Trushina E, Fiesel FC, Christenson TA, Schroeder AS, Zhang L, Bowles E, Behrouz B, Lincoln SJ, Beevers JE, Milnerwood AJ, Kurti A, McLean PJ, Fryer JD, **Springer W**, Dickson DW, Farrer MJ, Melrose HL. Progressive dopaminergic alterations and mitochondrial abnormalities in LRRK2 G2019S knock-in mice. Neurobiol Dis. 2015 Jun; 78:172-95. Epub 2015 Mar 31. PMID: 25836420 PMCID: PMC4526103 DOI: 10.1016/j.nbd.2015.02.031

Fiesel FC, Caulfield TR, Moussaud-Lamodiere EL, Ogaki K, Dourado DF, Flores SC, Ross OA, **Springer W**. Structural and Functional Impact of Parkinson Disease-Associated Mutations in the E3 Ubiquitin Ligase Parkin. Hum Mutat. 2015 Aug; 36 (8):774-86 Epub 2015 June 03 PMID: 25939424 PMCID: PMC4514554 DOI: 10.1002/humu.22808

Fiesel FC, Ando M, Hudec R, Hill AR, Castanedes-Casey M, Caulfield TR, Moussaud-Lamodiere EL, Stankowski JN, Bauer PO, Lorenzo-Betancor O, Ferrer I, Arbelo JM, Siuda J, Chen L, Dawson VL, Dawson TM, Wszolek ZK, Ross OA, Dickson DW, **Springer W**. (Patho-)physiological relevance of PINK1-dependent ubiquitin phosphorylation. EMBO Rep. 2015 Sep; 16 (9):1114-30 Epub 2015 July 10 PMID: 26162776 PMCID: PMC4576981 DOI: 10.15252/embr.201540514

Lorenzo-Betancor O, Ogaki K, Soto-Ortolaza AI, Labbe C, Walton RL, Strongosky AJ, van Gerpen JA, Uitti RJ, McLean PJ, **Springer W**, Siuda J, Opala G, Krygowska-Wajs A, Barcikowska M, Czyzewski K, McCarthy A, Lynch T, Puschmann A, Rektorova I, Sanotsky Y, Vilarino-Guell C, Farrer MJ, Ferman TJ, Boeve BF, Petersen RC, Parisi JE, Graff-Radford NR, Dickson DW, Wszolek ZK, Ross OA. DNAJC13 p.Asn855Ser mutation screening in Parkinson's disease and pathologically confirmed Lewy body disease patients. Eur J Neurol. 2015 Sep; 22 (9):1323-5 PMID: 26278106 PMCID: PMC4542017 DOI: 10.1111/ene.12770

Shi J, Fung G, Deng H, Zhang J, Fiesel FC, **Springer W**, Li X, Luo H. NBR1 is dispensable for PARK2-mediated mitophagy regardless of the presence or absence of SQSTM1. Cell Death Dis. 2015 Oct 29; 6:e1943 PMID: 26512954 PMCID: PMC4632303 DOI: 10.1038/cddis.2015.278

Ogaki K, Koga S, Heckman MG, Fiesel FC, Ando M, Labbe C, Lorenzo-Betancor O, Moussaud-Lamodiere EL, Soto-Ortolaza AI, Walton RL, Strongosky AJ, Uitti RJ, McCarthy A, Lynch T, Siuda J, Opala G, Rudzinska M, Krygowska-Wajs A, Barcikowska M, Czyzewski K, Puschmann A, Nishioka K, Funayama M, Hattori N, Parisi JE, Petersen RC, Graff-Radford NR, Boeve BF, **Springer W**, Wszolek ZK, Dickson DW, Ross OA. Mitochondrial targeting sequence variants of the CHCHD2 gene are a risk for Lewy body disorders. Neurology. 2015 Dec 8; 85 (23):2016-25 Epub 2015 Nov 11 PMID: 26561290 PMCID: PMC4676755 DOI: 10.1212/WNL.0000000000002170

Kim J, Fiesel FC, Belmonte KC, Hudec R, Wang WX, Kim C, Nelson PT, **Springer W**, Kim J. miR-27a and miR-27b regulate autophagic clearance of damaged mitochondria by targeting PTEN-induced putative kinase 1 (PINK1). Mol Neurodegener. 2016 Jul 26; 11 (1):55 Epub 2016 July 26 PMID: 27456084 PMCID: PMC4960690 DOI: 10.1186/s13024-016-0121-4

Fiesel FC, Hudec R, **Springer W**. Non-radioactive in vitro PINK1 Kinase Assays Using Ubiquitin or Parkin as Substrate. Bio Protoc. 2016 Oct 5; 6 (19) PMID: 28573163 PMCID: PMC5448420 DOI: 10.21769/BioProtoc.1946

Puschmann A, Fiesel FC, Caulfield TR#, Hudec R, Ando M, Truban D, Hou X, Ogaki K, Heckman MG, James ED, Swanberg M, Jimenez-Ferrer I, Hansson O, Opala G, Siuda J, Boczarska-Jedynak M, Friedman A, Koziorowski D, Rudzinska-Bar M, Aasly JO, Lynch T, Mellick GD, Mohan M, Silburn PA, Sanotsky Y, Vilarino-Guell C, Farrer MJ, Chen L, Dawson VL, Dawson TM, Wszolek ZK, Ross OA, **Springer W**. Heterozygous PINK1 p.G411S increases risk of Parkinson's disease via a dominant-negative mechanism. Brain. 2017 Jan; 140 (1):98-117 Epub 2016 Nov 02 PMID: 27807026 PMCID: PMC5379862 DOI: 10.1093/brain/aww261

Lee Y, Stevens DA, Kang SU, Jiang H, Lee YI, Ko HS, Scarffe LA, Umanah GE, Kang H, Ham S, Kam TI, Allen K, Brahmachari S, Kim JW, Neifert S, Yun SP, Fiesel FC, **Springer W**, Dawson VL, Shin JH, Dawson TM. PINK1 Primes Parkin-Mediated Ubiquitination of PARIS in Dopaminergic Neuronal Survival. Cell Rep. 2017 Jan 24; 18 (4):918-932 PMID: 28122242 PMCID: PMC5312976 DOI: 10.1016/j.celrep.2016.12.090

Ando M, Fiesel FC, Hudec R, Caulfield TR, Ogaki K, Gorka-Skoczylas P, Koziorowski D, Friedman A, Chen L, Dawson VL, Dawson TM, Bu G, Ross OA, Wszolek ZK, **Springer W**. The PINK1 p.I368N mutation affects protein stability and ubiquitin kinase activity. Mol Neurodegener. 2017 Apr 24; 12 (1):32 PMID: 28438176 PMCID: PMC5404317 DOI: 10.1186/s13024-017-0174-z

Puschmann A, Fiesel FC, Caulfield TR#, Hudec R, Ando M, Truban D, Hou X, Ogaki K, Heckman MG, James ED, Swanberg M, Jimenez-Ferrer I, Hansson O, Opala G, Siuda J, Boczarska-Jedynak M, Friedman A, Koziorowski D, Rudzinska-Bar M, Aasly JO, Lynch T, Mellick GD, Mohan M, Silburn PA, Sanotsky Y, Vilarino-Guell C, Farrer MJ, Chen L, Dawson VL, Dawson TM, Wszolek ZK, Ross OA, **Springer W**. Reply: Heterozygous PINK1 p.G411S in rapid eye movement sleep behaviour disorder. Brain 2017 Jun 1; 140 (6):e33 PMID: 28379295 PMCID: PMC6248563 DOI: 10.1093/brain/awx077

Hauser DN, Mamais A, Conti MM, Primiani CT, Kumaran R, Dillman AA, Langston RG, Beilina A, Garcia JH, Diaz-Ruiz A, Bernier M, Fiesel FC, Hou X, **Springer W**, Li Y, de Cabo R, Cookson MR. Hexokinases link DJ-1 to the PINK1/parkin pathway. Mol Neurodegener. 2017 Sep 29; 12 (1):70 Epub 2017 Sept 29 PMID: 28962651 PMCID: PMC5622528 DOI: 10.1186/s13024-017-0212-x

Fiesel FC, James ED, Hudec R, **Springer W**. Mitochondrial targeted HSP90 inhibitor Gamitrinib-TPP (G-TPP) induces PINK1/Parkin-dependent mitophagy. Oncotarget. 2017 Dec 5; 8 (63):106233-106248 Epub 2017 Nov 06 PMID: 29290944 PMCID: PMC5739729 DOI: 10.18632/oncotarget.22287

Hou X, Fiesel FC, Truban D, Castanedes Casey M, Lin WL, Soto AI, Tacik P, Rousseau LG, Diehl NN, Heckman MG, Lorenzo-Betancor O, Ferrer I, Arbelo JM, Steele JC, Farrer MJ, Cornejo-Olivas M, Torres L, Mata IF, Graff-Radford NR, Wszolek ZK, Ross OA, Murray ME, Dickson DW, **Springer W**. Age- and disease-dependent increase of the mitophagy marker phospho-ubiquitin in normal aging and Lewy body disease. Autophagy. 2018; 14 (8):1404-1418 Epub 2018 July 28 PMID: 29947276 PMCID: PMC6372017 DOI: 10.1080/15548627.2018.1461294

Lee SB, Kim JJ, Han SA, Fan Y, Guo LS, Aziz K, Nowsheen S, Kim SS, Park SY, Luo Q, Chung JO, Choi SI, Aziz A, Yin P, Tong SY, Fiesel FC, **Springer W**, Zhang JS, Lou Z. The AMPK-Parkin axis negatively regulates necroptosis and tumorigenesis by inhibiting the necrosome. Nat Cell Biol. 2019 Aug; 21 (8):940-951 Epub 2019 July 29 PMID: 31358971 PMCID: PMC6679774 DOI: 10.1038/s41556-019-0356-8

Luo S, Kang SS, Wang ZH, Liu X, Day JX, Wu Z, Peng J, Xiang D, **Springer W**, Ye K. Akt Phosphorylates NQO1 and Triggers its Degradation, Abolishing Its Antioxidative Activities in Parkinson's Disease. J Neurosci. 2019 Sep 11; 39 (37):7291-7305 Epub 2019 July 29 PMID: 31358653 PMCID: PMC6759025 DOI: 10.1523/JNEUROSCI.0625-19.2019

Milanowski LM, Oshinaike O, Broadway BJ, Lindemann JA, Soto-Beasley AI, Walton RL, Hanna Al-Shaikh R, Strongosky AJ, Fiesel FC, Ross OA, **Springer W**, Ogun SA, Wszolek ZK. Early-Onset Parkinson Disease Screening in Patients From Nigeria. Front Neurol. 2020; 11:594927 Epub 2021 Jan 14 PMID: 33519679 PMCID: PMC7841006 DOI: 10.3389/fneur.2020.594927

Park JH, Burgess JD, Faroqi AH, DeMeo NN, Fiesel FC, **Springer W**, Delenclos M, McLean PJ. Alpha-synuclein-induced mitochondrial dysfunction is mediated via a sirtuin 3-dependent pathway. Mol Neurodegener. 2020 Jan 13; 15 (1):5 PMID: 31931835 PMCID: PMC6956494 DOI: 10.1186/s13024-019-0349-x

Soto-Beasley AI, Walton RL, Valentino RR, Hook PW, Labbe C, Heckman MG, Johnson PW, Goff LA, Uitti RJ, McLean PJ, **Springer W**, McCallion AS, Wszolek ZK, Ross OA. Screening non-MAPT genes of the Chr17q21 H1 haplotype in Parkinson's disease. Parkinsonism Relat Disord. 2020 Sep; 78:138-144 Epub 2020 Aug 01 PMID: 32829096 PMCID: PMC7686230 DOI: 10.1016/j.parkreldis.2020.07.022

Hou X, Watzlawik JO, Cook C, Liu CC, Kang SS, Lin WL, DeTure M, Heckman MG, Diehl NN, Al-Shaikh FSH, Walton RL, Ross OA, Melrose HL, Ertekin-Taner N, Bu G, Petrucelli L, Fryer JD, Murray ME, Dickson DW, Fiesel FC, **Springer W**. Mitophagy alterations in Alzheimer's disease are associated with granulovacuolar degeneration and early tau pathology. Alzheimers Dement. 2020 Oct 8; 17 (3):417-30 Epub 2020 Oct 08 PMID: 33090691 PMCID: PMC8048674 DOI: 10.1002/alz.12198

Watzlawik JO, Hou X, Truban D, Fricova D, Ramnarine C, Barodia SK, Gendron TF, Heckman MG, DeTure M, Siuda J, Wszolek ZK, Scherzer CR, Ross OA, Bu G, Dickson DW, Goldberg MS, Fiesel FC, **Springer W**. Sensitive ELISA-based detection method for the mitophagy marker p-S65-Ub in human cells, autopsy brain, and blood samples. Autophagy. 2021 Sep; 17 (9):2613-2628 Epub 2020 Oct 28 PMID: 33112198 PMCID: PMC8496550 DOI: 10.1080/15548627.2020.1834712

Milanowski LM, Hou X, Bredenberg JM, Fiesel FC, Cocker LT, Soto-Beasley AI, Walton RL, Strongosky AJ, Faroqi AH, Barcikowska M, Boczarska-Jedynak M, Dulski J, Fedoryshyn L, Janik P, Potulska-Chromik A, Karpinsky K, Krygowska-Wajs A, Lynch T, Olszewska DA, Opala G, Pulyk A, Rektorova I, Sanotsky Y, Siuda J, Widlak M, Slawek J, Rudzinska-Bar M, Uitti R, Figura M, Szlufik S, Rzonca-Niewczas S, Podgorska E, McLean PJ, Koziorowski D, Ross OA, Hoffman-Zacharska D, **Springer W**, Wszolek ZK. Cathepsin B p.Gly284Val Variant in Parkinson's Disease Pathogenesis. Int J Mol Sci. 2022 Jun 25; 23 (13) Epub 2022 June 25 PMID: 35806091 PMCID: PMC9266886 DOI: 10.3390/ijms23137086

Broadway BJ, Boneski PK, Bredenberg JM, Kolicheski A, Hou X, Soto-Beasley AI, Ross OA, **Springer W**, Fiesel FC. Systematic Functional Analysis of PINK1 and PRKN Coding Variants. Cells. 2022 Aug 5; 11 (15) Epub 2022 Aug 05 PMID: 35954270 PMCID: PMC9367835 DOI: 10.3390/cells11152426

Wu Z, Berlemann LA, Bader V, Sehr DA, Dawin E, Covallero A, Meschede J, Angersbach L, Showkat C, Michaelis JB, Munch C, Rieger B, Namgaladze D, Herrera MG, Fiesel FC, **Springer W**, Mendes M, Stepien J, Barkovits K, Marcus K, Sickmann A, Dittmar G, Busch KB, Riedel D, Brini M, Tatzelt J, Cali T, Winklhofer KF. LUBAC assembles a ubiquitin signaling platform at mitochondria for signal amplification and transport of NF-kappaB to the nucleus. EMBO J. 2022 Dec 15; 41 (24):e112006 Epub 2022 Nov 18 PMID: 36398858 PMCID: PMC9753471 DOI: 10.15252/embj.2022112006

Stevens MU, Croteau N, Eldeeb MA, Antico O, Zeng ZW, Toth R, Durcan TM, **Springer W**, Fon EA, Muqit MM, Trempe JF. Structure-based design and characterization of Parkin-activating mutations. Life Sci Alliance. 2023 Jun; 6 (6) Epub 2023 Mar 20 PMID: 36941054 PMCID: PMC10027901 DOI: 10.26508/lsa.202201419

Fiesel FC, Fricova D, Hayes CS, Coban MA, Hudec R, Bredenberg JM, Broadway BJ, Markham BN, Yan T, Boneski PK, Fiorino G, Watzlawik JO, Hou X, McCarty AM, Lewis-Tuffin LJ, Zhong J, Madden BJ, Ordureau A, An H, Puschmann A, Wszolek ZK, Ross OA, Harper JW, Caulfield TR, **Springer W**. Substitution of PINK1 Gly411 modulates substrate receptivity and turnover. Autophagy. 2023 Jun; 19 (6):1711-1732 Epub 2022 Dec 05 PMID: 36469690 PMCID: PMC10262784 DOI: 10.1080/15548627.2022.2151294

Ligezka AN, Budhraja R, Nishiyama Y, Fiesel FC, Preston G, Edmondson A, Ranatunga W, Van Hove JLK, Watzlawik JO, **Springer W**, Pandey A, Morava E, Kozicz T. Interplay of Impaired Cellular Bioenergetics and Autophagy in PMM2-CDG. Genes (Basel). 2023 Aug 4; 14 (8) Epub 2023 Aug 04 PMID: 37628636 PMCID: PMC10454768 DOI: 10.3390/genes14081585

Hou X, Chen TH, Koga S, Bredenberg JM, Faroqi AH, Delenclos M, Bu G, Wszolek ZK, Carr JA, Ross OA, McLean PJ, Murray ME, Dickson DW, Fiesel FC, **Springer W**. Alpha-synuclein-associated changes in PINK1-PRKN-mediated mitophagy are disease context dependent. Brain Pathol. 2023 Sep; 33 (5):e13175 Epub 2023 May 31 PMID: 37259617 PMCID: PMC10467041 DOI: 10.1111/bpa.13175

Baninameh Z, Watzlawik JO, Hou X, Richardson T, Kurchaba NW, Yan T, Di Florio DN, Fairweather D, Kang L, Nguyen JH, Kanekiyo T, Dickson DW, Noda S, Sato S, Hattori N, Goldberg MS, Ganley IG, Stauch KL, Fiesel FC, **Springer W**. Alterations of PINK1-PRKN signaling in mice during normal aging. Autophagy Rep. 2024; 3 (1) Epub 2024 Dec 07 PMID: 40008113 PMCID: PMC11855339 DOI: 10.1080/27694127.2024.2434379

Piat C, Ross OA, **Springer W**, Benarroch EE, Layne Moore J, Lauer E, Niu Z, Savica R. Valosin-containing-protein pathogenic variant p.R487H in Parkinson's disease. Clin Park Relat Disord. 2024; 10:100236 Epub 2024 Jan 18 PMID: 38283104 PMCID: PMC10818073 DOI: 10.1016/j.prdoa.2024.100236

Walton RL, Koga S, Beasley AI, White LJ, Griesacker T, Murray ME, Kasanuki K, Hou X, Fiesel FC, **Springer W**, Uitti RJ, Fields JA, Botha H, Ramanan VK, Kantarci K, Lowe VJ, Jack CR, Ertekin-Taner N, Savica R, Graff-Radford J, Petersen RC, Parisi JE, Reichard RR, Graff-Radford NR, Ferman TJ, Boeve BF, Wszolek ZK, Dickson DW, Ross OA, Heckman MG. Role of GBA variants in Lewy body disease neuropathology. Acta Neuropathol. 2024 Mar 12; 147 (1):54 PMID: 38472443 PMCID: PMC11049671 DOI: 10.1007/s00401-024-02699-w

Islam NN, Weber CA, Coban M, Cocker LT, Fiesel FC, **Springer W**, Caulfield TR. In Silico Investigation of Parkin-Activating Mutations Using Simulations and Network Modeling. Biomolecules. 2024 Mar 19; 14 (3) PMID: 38540783 PMCID: PMC10968616 DOI: 10.3390/biom14030365

Rasool S, Shomali T, Truong L, Croteau N, Veyron S, Bustillos BA, **Springer W**, Fiesel FC, Trempe JF. Identification and structural characterization of small molecule inhibitors of PINK1. Sci Rep. 2024 Apr 2; 14 (1):7739 Epub 2024 Apr 02 PMID: 38565869 PMCID: PMC10987619 DOI: 10.1038/s41598-024-58285-3

Watzlawik JO, Fiesel FC, Fiorino G, Bustillos BA, Baninameh Z, Markham BN, Hou X, Hayes CS, Bredenberg JM, Kurchaba NW, Fricova D, Siuda J, Wszolek ZK, Noda S, Sato S, Hattori N, Prasad AA, Kirik D, Fox HS, Stauch KL, Goldberg MS, **Springer W**. Basal activity of PINK1 and PRKN in cell models and rodent brain. Autophagy. 2024 May; 20 (5):1147-1158 Epub 2023 Dec 02 PMID: 38041584 PMCID: PMC11135862 DOI: 10.1080/15548627.2023.2286414

Watzlawik JO, Hou X, Richardson T, Lewicki SL, Siuda J, Wszolek ZK, Cook CN, Petrucelli L, DeTure M, Dickson DW, Antico O, Muqit MMK, Fishman JB, Pirani K, Kumaran R, Polinski NK, Fiesel FC, **Springer W**. Development and characterization of phospho-ubiquitin antibodies to monitor PINK1-PRKN signaling in cells and tissue. Autophagy. 2024 Sep; 20 (9):2076-2091 Epub 2024 May 27 PMID: 38802071 PMCID: PMC11346534 DOI: 10.1080/15548627.2024.2356490

Bustillos BA, Cocker LT, Coban MA, Weber CA, Bredenberg JM, Boneski PK, Siuda J, Slawek J, Puschmann A, Narendra DP, Graff-Radford NR, Wszolek ZK, Dickson DW, Ross OA, Caulfield TR, **Springer W**, Fiesel FC. Structural and Functional Characterization of the Most Frequent Pathogenic PRKN Substitution p.R275W. Cells. 2024 Sep 13; 13 (18) Epub 2024 Sept 13 PMID: 39329724 PMCID: PMC11430725 DOI: 10.3390/cells13181540

Zhu X, Wu Y, Li Y, Zhou X, Watzlawik JO, Chen YM, Raybuck AL, Billadeau DD, Shapiro VS, **Springer W**, Sun J, Boothby MR, Zeng H. The nutrient-sensing Rag-GTPase complex in B cells controls humoral immunity via TFEB/TFE3-dependent mitochondrial fitness. Nat Commun. 2024 Nov 23; 15 (1):10163 PMID: 39580479 PMCID: PMC11585635 DOI: 10.1038/s41467-024-54344-5

Yan T, Heckman MG, Craver EC, Liu CC, Rawlinson BD, Wang X, Murray ME, Dickson DW, Ertekin-Taner N, Lou Z, Bu G, **Springer W**, Fiesel FC. The UFMylation pathway is impaired in Alzheimer's disease. Mol Neurodegener. 2024 Dec 18; 19 (1):97 PMID: 39696466 PMCID: PMC11656649 DOI: 10.1186/s13024-024-00784-y

Franz TM, Punathil RK, Soto-Beasley AI, Strongosky A, Walton RL, Kim-Hellmuth S, **Springer W**, Dulski J, Ross OA, Jaramillo-Koupermann G, Alarcon F, Wszolek ZK. Screening for PRKN and PINK1 mutations in Ecuadorian patients with early-onset Parkinson's Disease. Neurol Neurochir Pol. 2025; 59 (1):56-61 PMID: 40013651 DOI: 10.5603/pjnns.104123

Naddaf E, Nguyen TKO, Watzlawik JO, Gao H, Hou X, Fiesel FC, Mandrekar J, Kokesh E, Harmsen WS, Lanza IR, **Springer W**, Trushina E. NLRP3 Inflammasome Activation and Altered Mitophagy Are Key Pathways in Inclusion Body Myositis. J Cachexia Sarcopenia Muscle. 2025 Feb; 16 (1):e13672 PMID: 39723571 PMCID: PMC11669947 DOI: 10.1002/jcsm.13672

Bagnoli E, Lin YE, Burel S, Jaimon E, Antico O, Themistokleous C, Nikoloff JM, Squires S, Morella I, Watzlawik JO, Fiesel FC, **Springer W**, Tonelli F, Lis P, Brooks SP, Dunnett SB, Brambilla R, Alessi DR, Pfeffer SR, Muqit MMK. Endogenous LRRK2 and PINK1 function in a convergent neuroprotective ciliogenesis pathway in the brain. Proc Natl Acad Sci U S A. 2025 Feb 4; 122 (5):e2412029122 Epub 2025 Jan 28 PMID: 39874296 PMCID: PMC11804522 DOI: 10.1073/pnas.2412029122

Baninameh Z, Watzlawik JO, Bustillos BA, Fiorino G, Yan T, Lewicki SL, Zhang H, Dickson DW, Siuda J, Wszolek ZK, **Springer W**, Fiesel FC. Development and validation of a sensitive sandwich ELISA against human PINK1. Autophagy. 2025 Feb 6; 1-16 Epub 2025 Feb 06 PMID: 39912496 DOI: 10.1080/15548627.2025.2457915

Richardson T, Hou X, Fiesel FC, Wszolek ZK, Dickson DW, **Springer W**. Hippocampal mitophagy alterations in MAPT-associated frontotemporal dementia with parkinsonism. Acta Neuropathol Commun. 2025 Feb 24; 13 (1):41 PMID: 39994734 PMCID: PMC11849217 DOI: 10.1186/s40478-025-01955-8

Reviews

Springer W, Kahle PJ. Mechanisms and models of alpha-synuclein-related neurodegeneration. Curr Neurol Neurosci Rep. 2006 Sep; 6(5):432-6. PMID: 16928354

Springer W, Kahle PJ. Regulation of PINK1-Parkin-mediated mitophagy. Autophagy. 2011 Mar; 7(3):266-78. PMID: 21187721

Klionsky DJ, …, **Springer W**, …. Guidelines for the use and interpretation of assays for monitoring autophagy. Autophagy. 2012 Apr; 8 (4):445-544 PMID: 22966490 PMCID: PMC3404883 DOI: 10.4161/auto.19496

Caulfield TR, Fiesel FC, **Springer W**. Activation of the E3 ubiquitin ligase Parkin. Biochem Soc Trans. 2015 Apr; 43 (2):269-74 PMID: 25849928 PMCID: PMC4859148 DOI: 10.1042/BST20140321

Klionsky DJ, … **Springer W**, ….. Guidelines for the use and interpretation of assays for monitoring autophagy (3rd edition). Autophagy. 2016; 12 (1):1-222 PMID: 26799652 PMCID: PMC4835977 DOI: 10.1080/15548627.2015.1100356

Truban D, Hou X, Caulfield TR, Fiesel FC, **Springer W**. PINK1, Parkin, and Mitochondrial Quality Control: What can we Learn about Parkinson's Disease Pathobiology? J Parkinsons Dis. 2017; 7 (1):13-29 PMID: 27911343 PMCID: PMC5302033 DOI: 10.3233/JPD-160989

Hou X, Watzlawik JO, Fiesel FC, **Springer W**. Autophagy in Parkinson's Disease. J Mol Biol. 2020 Apr 3; 432 (8):2651-2672 Epub 2020 Feb 13 PMID: 32061929 PMCID: PMC7211126 DOI: 10.1016/j.jmb.2020.01.037

Kolicheski A, Turcano P, Tamvaka N, McLean PJ, **Springer W**, Savica R, Ross OA. Early-Onset Parkinson's Disease: Creating the Right Environment for a Genetic Disorder. J Parkinsons Dis. 2022; 12(8):2353-2367. PMID: 36502340 PMCID: PMC9837689 DOI: 10.3233/JPD-223380

Book Chapters

Fiesel FC, Caulfield TR, Ross OA, **Springer W**. Parkin. In: Sangdun Choi. Encyclopedia of Signaling Molecules.2nd ed. Springer; 2017.p. 1-9

Watzlawik JO, Hou X, Fiesel FC, **Springer W**. Mitochondrial Autophagy in Brain. In: Gendelman HE, Ikezu T, editor(s). Neuroimmune Pharmacology and Therapeutics. 3rd ed. Springer; 2024. p. 131-146.

Editorials

Fiesel FC, **Springer W**. Disease relevance of phosphorylated ubiquitin (p-S65-Ub). Autophagy. 2015 Nov 2; 11 (11):2125-2126

Charleen T. Chu - relevant works

Peer-reviewed primary publications

JH Zhu, F Guo, J Shelburne, S Watkins & **CT Chu.** (2003) Localization of phosphorylated ERK/MAP kinases to mitochondria and autophagosomes in Lewy body diseases. *Brain Pathol,* **13:** 473-481.

CT Chu & JH Zhu (2003) Subcellular compartmentalization of P-ERK in the Lewy body disease substantia nigra. *Ann NY Acad Sci* **991:** 288-290.

ML McClure, DA Linseman, **CT Chu**, PA Barker, RJ Bouchard, SS Le, TA Laessig and KA Heidenreich. (2004) The p75 neurotrophin receptor mediates autophagy and death of cerebellar Purkinje neurons. *J. Neurosci,* **24(**19): 4498-4509.

JH Zhu, C Horbinski, F Guo, S Watkins, Y Uchiyama & **CT Chu** (2007). Regulation of autophagy by extracellular signal regulated protein kinases during 1-methyl-4-phenylpyridinium injury. *Am J. Pathol,* **170:** 75-86.

E Thiels, NN Urban, GR Gonzalez-Burgos, BI Kanterewicz, G Barrionuevo, **CT Chu**, TD Oury & E Klann. (2000) Impairment of long-term potentiation and associative memory in mice that overexpress extracellular superoxide dismutase. *J Neurosci* **20:** 7631-7639.

D Hu, P Cao, E Thiels, **CT Chu**, G Wu, TD Oury & E Klann. (2007) Hippocampal long-term potentiation, memory and longevity in mice that overexpress mitochondrial superoxide dismutase. *Neurobiol Learning Mem*, **87:** 372-384.

Y Lai, RW Hickey, Y Chen, H Bayir, M Sullivan, **CT Chu**, PM Kochanek, CE Dixon, LW Jenkins, SH Graham, SC Watkins, RSB Clark. (2008) Autophagy is increased after traumatic brain injury in mice and is partially inhibited by the antioxidant γ-glutamylcysteinyl ethyl ester. *J Cereb Blood Flow Metab*, **28:** 540-550.

ED Plowey, SJ Cherra III, Y-J Liu & **CT Chu** (2008) Role of autophagy in G2019S-LRRK2-associated neurite shortening in differentiated SH-SY5Y cells. *J Neurochem* **105:** 1048-1056.

RK Dagda, J Zhu, SM Kulich & **CT Chu.** (2008) Mitochondrially localized ERK2 regulates mitophagy and autophagic cell stress. *Autophagy,* **4:** 770-782.

L Du, RW Hickey, H Bayir, SC Watkins, VA Tyurin, F Guo, PM Kochanek, LW Jenkins, J Ren, G Gibson, **CT Chu**, VE Kagan & RSB Clark. (2009) Starving neurons show sex difference in autophagy. *J Biol Chem*, **284:** 2383-2396.

RK Dagda, SJ Cherra III, SM Kulich, A Tandon, D Park & **CT Chu**. (2009) Loss of PINK1 function promotes mitophagy through effects on oxidative stress and mitochondrial fission. *J Biol Chem* **284:** 13843-13855.

H Bayir, A Kapralov, J Jiang, Z Huang, YY Tyurina, VA Tyurin, Q Zhao, NA Belikova, II Vlasova, A Maeda, J Zhu, HM Na, PG Mastroberardino, LJ Sparvero, AA Amoscato, **CT Chu**, JT Greenamyre & VE Kagan (2009) Peroxidase mechanism of lipid dependent cross-linking of synuclein with cytochrome c: Protection against apoptosis versus delayed oxidative stress in Parkinson disease. *J Biol Chem* **284:** 15951-15969.

SJ Cherra III, SM Kulich, G Uechi, M Balasubramani, J Mountzouris, BW Day & **CT Chu**. (2010) Regulation of the autophagy protein LC3 by phosphorylation. *J. Cell. Biol*. **190:** 533-539.

VS Van Laar, BA Arnold, SJ Cassady, **CT Chu**, EA Burton & SB Berman. (2011) Bioenergetics of neurons inhibit the translocation response of Parkin following rapid mitochondrial depolarization. *Hum Mol Genet* **20:** 927-940.

RK Dagda, A Gusdon, I Pien, S Strack, S Green, B Van Houten, SJ Cherra III & **CT Chu**. (2011) Mitochondrially localized PKA reverses mitochondrial pathology and dysfunction in a cellular model of Parkinson's disease. *Cell Death Differ* **18:** 1914-1923.

AM Gusdon, J Zhu, B Van Houten & **CT Chu**. (2012) ATP13A2 regulates mitochondrial bioenergetics through macroautophagy. *Neurobiol Dis* **45:** 962-972.

J Zhu, A Gusdon, H Cimen, B Van Houten, E Koc & **CT Chu.** (2012) Impaired mitochondrial biogenesis contributes to depletion of functional mitochondria in chronic MPP+ toxicity. *Cell Death Dis* **3:** e312, 1-10.

SJ Cherra III, E Steer, AM Gusdon, K Kiselyov & **CT Chu**. (2013) Mutant LRRK2 elicits calcium imbalance and depletion of dendritic mitochondria in neurons. *Am J Pathol,* **182:** 474-484.

CT Chu*, J Ji, RK Dagda, JF Jiang, YY Tyurina, AA Kapralov, VA Tyurin, N Yanamala, IH Shrivastava, D Mohammadyani, KZQ Wang, J Zhu, J Klein-Seetharaman, K Balasubramanian, AA Amoscato, G Borisenko, Z Huang, AM Gusdon, A Cheikhi, EK Steer, R Wang, C Baty, S Watkins, I Bahar, H Bayır* & VE Kagan* (2013) Cardiolipin externalization to the outer mitochondrial membrane acts as an elimination signal for mitophagy in neuronal cells. *Nature Cell Biol* **15:**1197-1205.

C Kamga-Pride, L Mo, K Quesnelle, RK Dagda, D Murillo, L Geary, C Corey, R Portella, S Zharikov, C St Croix, S Maniar, **CT Chu**, N Khoo & S Shiva (2014) Nitrite activates protein kinase A in normoxia to mediate mitochondrial fusion and tolerance to ischemia reperfusion. *Cardiovascular Res*, **101:** 57-68.

RK Dagda, I Pien, R Wang, J Zhu, KZQ Wang, J Callio, TD Banerjee, RY Dagda & **CT Chu** (2014) Beyond the mitochondrion: cytosolic PINK1 remodels dendrites through Protein Kinase A. *J Neurochem* **128:** 864-877.

BB Chen, TA Coon, JR Glasser, C Zou, B Ellis, T Das, AC McKelvey, S Rajbhandari, T Lear, C Kamga, S Shiva, CJ Li, JM Pilewski, J Callio, **CT Chu**, A Ray, P Ray, YY Tyurina, VE Kagan, and RK Mallampalli. (2014) E3 ligase subunit Fbxo15 and PINK1 kinase regulate cardiolipin synthase 1 stability and mitochondrial function in pneumonia. *Cell Reports* **7:** 476-487.

KZQ Wang, J Zhu, RK Dagda, G Uechi, SJ Cherra III, AM Gusdon, M Balasubramani & **CT Chu**. (2014) ERK-mediated phosphorylation of TFAM downregulates mitochondrial transcription. *Mitochondrion*, **17:** 132-140.

E Plowey, JW Johnson, D Eisenberg, NM Valentino, YJ Liu & **CT Chu**. (2014) Mutant LRRK2 enhances glutamatergic synapse activity and evokes excitotoxic dendrite degeneration. *Biochim. Biophys. Acta (Molecular Basis of Disease)* **1842:** 1596-1603.

M Bueno, M Mosher, C Kamga, C Corey, D Stolz, C St. Croix, M Rojas, S Shiva, **CT Chu** & AL Mora. (2015) PINK1 deficiency impairs mitochondrial homeostasis and promotes lung fibrosis. *J Clin Invest* **125:** 521-538.

AK Au, Y Chen, L Du, CM Smith, MD Manole, SA Baltagi, **CT Chu**, RK Aneja, H Bayır, PM Kochanek & RSB Clark. (2015) Ischemia-induced autophagy contributes to neurodegeneration in cerebellar Purkinje cells in the developing brain and in primary cortical neurons in vitro. *Biochim. Biophys. Acta (Molecular Basis of Disease)* **1852:** 1902-1911.

M Kostic, MHR Ludtmann, H Bading, M Hershfinkel, E Steer, **CT Chu**, AY Abramov & I Sekler (2015) PKA phosphorylation of NCLX reverses mitochondrial calcium overload and depolarization, promoting survival of PINK1-deficient dopaminergic neurons. *Cell Reports* **13:** 376-386.

VE Kagan, J Jiang, Z Huang, YY Tyrurina, C Desbourdes, C Cottet-Rousselle, H Dar, M Verma, VA Tyurin, AA Kapralov, A Cheikhi, G Mao, D Stolz, CM S. Croix, S Watkins, Z Shen, Y , ML Greenberg, M Tokarska-Schlattner, M Boissan, M-L Lacombe, RM Epand, **CT Chu**, R Mallampalli, H Bayır, U Schlattner. (2016) NDPK-D (NM23-H4)-dependent externalization of cardiolipin during elimination of depolarized mitochondria. *Cell Death Differ* **23:** 1140-1151.

K Banerjee, S Munshi, H Xu, DE Frank, HL Chen, **CT Chu**, J Yang, S Cho, VE Kagan, TT Denton, YY Tyurina, JF Jiang, GE Gibson. (2016) Mild mitochondrial metabolic deficits by a-ketoglutarate dehydrogenase inhibition cause prominent changes in intracellular autophagic signaling: Potential role in the pathobiology of Alzheimer's disease. *Neurochem Int* 96:32-45.

S Ravi, KA Pena, **CT Chu**, K Kiselyov. (2016) Biphasic regulation of lysosomal exocytosis by oxidative stress. *Cell Calcium* **60:** 356-362.

T Das Banerjee, RY Dagda, M Dagda, **CT Chu**, E Vasquez-Mayorga, M Rice, RK Dagda. (2017) PINK1 regulates mitochondrial trafficking in dendrites of cortical neurons through mitochondrial PKA *J Neurochem* **142:** 545-559.

M Verma, J Callio, PA Otero, I Sekler, ZP Wills & **CT Chu**. (2017) Mitochondrial calcium dysregulation contributes to dendrite degeneration mediated by PD/LBD-associated LRRK2 mutants. *J. Neurosci* **37:** 11151-11165.

M Yang, Y Wang, G Liang, Z Xu, **CT Chu**, H Wei. (2018) Alzheimer's presenilin-1 mutation render neurons vulnerable to propofol neurotoxicity by calcium dysregulation and impaired autophagy. *J Alzheimers Dis* **67:** 137-147.

KZQ Wang*, E Steer*, PA Otero*, NW Bateman, MH Cheng, AL Scott, C Wu, I Bahar, YT Shih, YP Hsueh & **CT Chu**. (2018) PINK1 interacts with VCP/p97 and activates PKA to promote NSFL1C/p47 phosphorylation and dendritic arborization in neurons. *eNeuro*, **5:** ENEURO.0466-18.2018, 1-16.*co-first authors, equal contributions.

Z Xu, Y Wang, G Liang, Z Liu, W Ma, CT Chu, H Wei. (2020) Propofol affects cell survival via regulation of autophagy in ATG5 and calcium dependent manner. *Acta Phamacol Sin*, **41**(3): 303-310.

M Verma, J Zhu, KZQ Wang & **CT Chu**. 2020. Chronic treatment with the complex I inhibitor MPP+ depletes endogenous PTEN-induced kinase 1 (PINK1) via upregulation of Bcl-2-associated athanogene 6 (BAG6). *J Biol Chem* **295:** 7865-7876.

Y Liu, TB Lear, M Verma, KZQ Wang, PA Otero, AC McKelvey, SR Dunn, E Steer, NW Bateman, C Wu, Y Jiang, NM Weathington, M Rojas, **CT Chu***, BB Chen*, RK Mallampalli*. 2020. Chemical inhibition of Fbxo7 reduces inflammation and confers neuroprotection by stabilizing the mitochondrial kinase PINK1. *JCI Insight* **5:** e131834. *co-senior authors, equal contributions

PA Otero, G Fricklas, A Nigam, BN Lizama, ZP Wills, JW Johnson & **CT Chu**. 2022. Endogenous PTEN-induced kinase 1 regulates dendritic architecture and spinogenesis. *J Neurosci* **42:** 7848-7860.

M Verma*, L Francis*, BN Lizama*, J Callio, G Fricklas, KZQ Wang, BA Kaufman, L D'Aiuto, DB Stolz, SC Watkins, VL Nimgaonkar, A Soto-Gutierrez, A Goldstein & **CT Chu**. 2023. iPSC-derived neurons from patients with *POLG* mutations exhibit decreased mitochondrial content and dendrite simplification. *Am J Pathol* **193:** 201-212. *Co-first authors

Reviews

CT Chu. (2006) Autophagic stress in neuronal injury and disease. *J Neuropath Exp Neurol,* **65:** 423-432.

K Kiselyov, JJ Jennings, Y Rbaibi & **CT Chu** (2007) Autophagy, mitochondria and cell death in lysosomal storage diseases. *Autophagy* **3:** 259-262.

CT Chu, J Zhu & R Dagda (2007) Beclin 1-independent pathway of damage-induced mitophagy and autophagic stress: Implications for neurodegeneration and cell death. *Autophagy,* **3:** 663-666.

RS Clark, H Bayir, **CT Chu**, SM Alber, PM Kochanek & SC Watkins (2008) Autophagy is increased in mice after traumatic brain injury and is detectable in human brain after trauma and critical illness. *Autophagy,* **4:** 88-90.

CT Chu (2008) Eaten alive: autophagy and neuronal cell death after hypoxia-ischemia. *Am J Pathol,* **172:** 284-287.

DJ Klionsky, H Abeliovich, P Agostinis ... **CT Chu** *et al*. (2008) Guidelines for the use and interpretation of assays for monitoring autophagy in higher eukaryotes. *Autophagy*, **4:** 151-175.

SJ Cherra III & **CT Chu**. (2008) Autophagy in neuroprotection and neurodegeneration: a question of balance. *Future Neurol,* **3:** 309-323.

CT Chu, Ed Plowey, RK Dagda, RW Hickey, SJ Cherra III & RSB Clark. (2009) Autophagy in neurite injury and neurodegeneration: in vitro and in vivo models. *Meth Enzymol,* **453:** 217-249.

SJ Cherra III, RK Dagda, A Tandon & **CT Chu**. (2009) Mitochondrial autophagy as a compensatory response to PINK1 deficiency. *Autophagy*, 5: 1213-1214.

CT Chu. (2010) Tickled PINK1: Mitochondrial homeostasis and autophagy in recessive parkinsonism. *Biochim Biophys Acta (Molecular Basis of Disease)* **1802:** 20-28.

RK Dagda & CT Chu (2009) Mitochondrial quality control: Insights on how Parkinson's disease related genes PINK1, Parkin, and Omi/HtrA2 interact to maintain mitochondrial homeostasis. *J Bioenerg Biomembr*, **41:** 473-479.

SJ Cherra III, RK Dagda & **CT Chu** (2010) Autophagy and Neurodegeneration: Survival at a cost? *Neuropathology and Applied Neurobiology* **36:** 125-132.

CT Chu (2010) A pivotal role for PINK1 and autophagy in mitochondrial quality control: Implications for Parkinson disease. *Human Mol Genetics* **19:** R28-R37.

ED Plowey & **CT Chu** (2011) Synaptic dysfunction in genetic models of Parkinson's disease: A Role for Autophagy? *Neurobiol Dis* **43:** 60-67.

AR Gusdon & **CT Chu** (2011) To eat or not to eat: Neuronal metabolism, mitophagy and Parkinson's disease. *Antioxidant Redox Signaling* **14:** 1979-1987.

CT Chu (2011) Diversity in the regulation of autophagy & mitophagy: Lessons from Parkinson's disease. *Parkinsons Dis*, Vol **2011**: Article ID 789431, 8 pages. doi:10.4061/2011/789431

J Zhu, RK Dagda & **CT Chu** (2011) Ch. 21. Monitoring mitophagy in neuronal cell cultures. In *Neurodegeneration: Methods and Protocols*. (G Manfredi & JK Fujita, Editors) Humana Press, New York, NY. ISBN: ISBN 978-1-61779-328-8; *Methods in Molecular Biology*, **793**: 325-339.

DJ Klionsky, FC Abdalla, H Abeliovich, RT Abraham… **CT Chu** … et al. (2012) Guidelines for the use and interpretation of assays for monitoring autophagy (2nd edition). *Autophagy* **8:** 445-544.

E Oczypok, TD Oury & **CT Chu**. (2013) It's a cell eat cell world: Autophagy and phagocytosis. *Am J Pathol* **182:** 612-622.

J Zhu, KZQ Wang & **CT Chu**. (2013) After the banquet: Mitochondrial biogenesis, mitophagy and cell survival. *Autophagy* **9:** 1663-76.

M Verma, EK Steer & **CT Chu**. (2013) ERKed by LRRK2: A cell biological perspective on hereditary and sporadic Parkinson's disease. *Biochim Biophys Acta (Molecular Basis of Disease)* **1842:** 1273-1281.

VE Kagan, **CT Chu**, YY Tyurina, A Cheikhi & H Bayir. (2014) Cardiolipin asymmetry, oxidation and signaling. *Chemistry and Physics of Lipids* **179:** 64-69.

CT Chu, H Bayir & VE Kagan. (2014) LC3 binds externalized cardiolipin on injured mitochondria to signal mitophagy in neurons: Implications for Parkinson disease. *Autophagy* **10:** 376-378.

EK Steer, MK Dail & **CT Chu**. (2015) Beyond mitophagy: cytosolic PINK1 as a messenger of mitochondrial health. *Antioxid Redox Signal* **22:** 1047-1059.

DJ Klionsky… **CT Chu** … et al. (2016) Guidelines for the use and interpretation of assays for monitoring autophagy (3rd edition). *Autophagy* **12:** 1-222.

CT Chu. (2018) Multiple pathways for mitophagy: A neurodegenerative conundrum for Parkinson's disease. *Neurosci Lett* **697:** 66-71.

M Verma, Z Wills & **CT Chu**. (2018) Excitatory dendritic mitochondrial calcium toxicity: Implications for Parkinson's and other neurodegenerative diseases. *Front Neurosci* **12:** 523 (12 pages).

CT Chu. (2019) Mechanisms of selective autophagy and mitophagy: Implications for neurodegenerative diseases. *Neurobiol Dis* **122:** 23-34.

DJ Klionsky… **CT Chu** … et al. (2021) Guidelines for the use and interpretation of assays for monitoring autophagy (4th edition). *Autophagy* **17:** 1-382.

BN Lizama & **CT Chu**. (2021) Neuronal Autophagy and Mitophagy in Parkinson's Disease. *Mol Aspects Med* **82:** 100972.

DJ Klionsky, G Petroni, RK Amaravadi…**CT Chu**…F Pietrocola. (2021) Autophagy in major human diseases. *The EMBO J* (2021) e108863.

M Verma, BN Lizama & **CT Chu**. (2021) Excitotoxicity, calcium and mitochondria: a triad in synaptic neurodegeneration. *Transl Neurodegener* **11:3**

CT Chu. (2022) Mitochondria in neurodegeneration. *Curr Opin Physiol.* **26:** 100532.

X Chen...**CT Chu**...D Tang. (2024) International consensus guidelines for the definition, detection, and interpretation of autophagy-dependent ferroptosis. *Autophagy.*

M McCabe, P Boya, RH Chen, **CT Chu**, MI Colombo, L Delgui, EL Eskelinen, M Hamasaski, M Hansen, C He, M Jaatela, A Kimchi, C Kraft, M Kundu, A Melendez, S Pattigre, T Proikas-Cezanne, S Sebti, K Simon, A Simonsen, SA Tooze, MI Vaccaro, X Wang, E White, Y Zhao, AM Cuervo. (2024) Women in Autophagy, an initiative to promote gender parity in science. *Nature Cell Biol.* **26:** 2009-2012

CT Chu. The role of autophagy in excitotoxicity, synaptic mitochondrial stress and neurodegeneration. *Autophagy Rep.* **In press.**

Books/book chapters

Autophagy of the Nervous System: Cellular Self-Digestion in Neurons and Neurological Diseases (Editors: Yue and Chu). World Scientific Press, Singapore, 2012. 440 pp. ISBN: 978-981-4350-44-0.

SJ Cherra III & **CT Chu**. (2012) Chapter 5. Maintaining autophagic balance: a role for brakes. In: Autophagy of the Nervous System: Cellular Self-Digestion in Neurons and Neurological Diseases (Editors: Yue and Chu). World Scientific Press, Singapore, pp. 105-125. ISBN: 978-981-4350-44-0.

ZH Sheng & **CT Chu.** (2012) Chapter 16. Neuronal mitochondrial transport and turnover via mitophagy. In: Autophagy of the Nervous System: Cellular Self-Digestion in Neurons and Neurological Diseases (Editors: Yue and Chu). World Scientific Press, Singapore, pp. 375-405. ISBN: 978-981-4350-44-0.

EK Steer, MK Dail & **CT Chu**. (2016) PINK1 as a sensor for mitochondrial function: Dual roles. In *The Functions, Disease-Related Dysfunctions and Therapeutic Targeting of Neuronal Mitochondria*. (VK Gribkoff, EA Jonas, JM Hardwick, Editors). John Wiley and Sons, Inc., New Jersey, pp. 240-253. ISBN: 978-1-118-70923-8

BN Lizama, PA Otero & **CT Chu** (2021) Ch. 4. PINK1: Multiple mechanisms of neuroprotection. In *Mechanisms of Cell Death and Approaches to Neuroprotection/Disease Modification in Parkinson's Disease*. (B Dehay & E Bezard, Editors) *Academic* Press, Elsevier, Cambridge, MA. ISBN: 978-0-323-89943-7; ISSN: 2666-7878. *International Review of Movement Disorders* **2:** 193-219.
